# Pin1 WW Domain Ligand Library Synthesized with an Easy Solid-Phase Phosphorylating Reagent

**DOI:** 10.1021/acs.biochem.4c00231

**Published:** 2024-10-08

**Authors:** Xingguo R. Chen, Ana Y. Mercedes-Camacho, Kimberly A. Wilson, Jill J. Bouchard, Jeffrey W. Peng, Felicia A. Etzkorn

**Affiliations:** † Department of Chemistry, Virginia Tech, Blacksburg, Virginia 24061, United States; ‡ Department of Chemistry and Biochemistry, University of Notre Dame, Notre Dame, Indiana 46556, United States

## Abstract

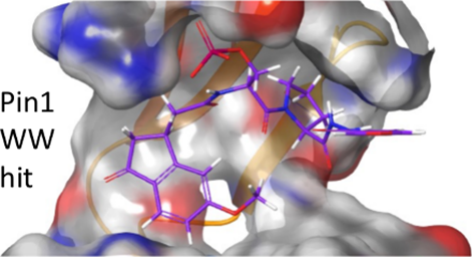

Cell cycle regulatory enzyme Pin1 both catalyzes pSer/Thr-*cis/trans*-Pro isomerization and binds the same motif separately in its WW domain. To better understand the function of Pin1, a way to separate these activities is needed. An unnatural peptide library, R^1^CO–pSer–Pro–NHR^2^, was designed to identify ligands specific for the Pin1 WW domain. A new solid-phase phosphorylating reagent (SPPR) containing a phosphoramidite functional group was synthesized in one step from Wang resin. The SPPR was used in the preparation of the library by parallel synthesis. The final 315-member library was screened with our WW-domain-specific, enzyme-linked enzyme-binding assay (ELEBA). Four of the best hits were resynthesized, and the competitive dissociation constants were measured by ELEBA. NMR chemical-shift perturbations (CSP) of ligands with ^15^N-labeled Pin1 were used to measure *K*_d_ for the best four ligands directly, demonstrating that they were specific Pin1 WW domain ligands. Models of the ligands bound to the Pin1 WW domain were used to visualize the mode of binding in the WW domain.

## Introduction

Pin1 is a phosphorylation-dependent enzyme that catalyzes the cis–trans isomerization of prolyl amide bonds,^1−4^ playing an important role in regulating the cell cycle,^1,5^ and many diseases, including cancer, Alzheimer’s, immune disorders, and infections.^6−8^ Pin1 contains two domains, the catalytic peptidyl-prolyl isomerase (PPIase) domain and WW (Trp-Trp) domain.^9−11^ The Pin1 WW domain and the PPIase domain both recognize and bind the pSer/pThr–Pro motif.^2,9,10^ Pin1 interacts with a large number of cell cycle and transcriptional regulatory proteins via both PPIase and WW domains.^2,9,12−14^

Ligands that block the Pin1 WW domain may be useful in elucidating the different roles of the PPIase and WW domains in regulatory processes in the cell.^13,15^ Numerous PPIase inhibitors have been reported,^16−28^ whereas only a few Pin1 WW domain selective ligands have been identified.^9,29−31^ Crystallography and NMR studies revealed the preference of the WW domain for binding ligands in the trans conformation.^9,32−35^ Molecular modeling of peptides in the cis conformation resulted in ligands with no binding affinity.^9^ The fact that the Pin1 PPIase domain is capable of binding both the cis and the trans substrates, while the Pin1 WW domain is specific for the trans conformation, suggests a possible role for the WW domain in the stabilization of trans ligands for downstream dephosphorylation by protein phosphatases.^36,37^

The WW domain of Pin1 likely serves a different regulatory purpose than the catalytic domain.^36,37^ Pin1 is the only PPIase in humans that has a type IV WW domain.^12^ Binding of these ligands to the WW domain can increase the fraction of substrates available for PPIase catalysis, both by blocking the WW domain, and by allosteric activation.^12,35,38^ Our objective is to identify ligands that selectively bind the Pin1 WW domain from a combinatorial pSer-Pro dipeptide library. The identification of ligands that block the Pin1 WW domain association with phosphoproteins represents a promising approach for the study of Pin1 in cell cycle regulation.^36,38,39^ The Pin1 WW domain recognizes the pSer/Thr–*trans*-Pro binding motif. However, the dipeptide, Ac–pThr–Pro–NH_2_ (*K*_d_ = 150 μM), is not a very good ligand of the Pin1 WW domain.^29^ Verdecia et al. reported that residues at the *N*- and *C*-termini of the binding motif affect the binding affinity.^9^

Solid-phase synthesis is a powerful tool to build libraries. However, in most solid-phase peptide synthesis, either the *C*- or the *N*-terminus of the peptide is bound to the resin, and coupling reactions only take place at the other end. Since our designed library has variations at both ends, and coupling reactions at both ends were needed, a solid-phase phosphorylating agent was the most efficient solution. The most commonly used phosphorylating reagents in solution-phase chemistry are phosphoramidites.^40,41^ By attaching the 2-cyanoethyl-*N*,*N*-diisopropyl phosphoramidite group to solid supports, Parang and co-workers synthesized three types of SPPRs, and successfully used them to phosphorylate carbohydrates and nucleosides.^42,43^ However, the reported SPPRs took four or five steps to prepare, and complex solid-phase chemistry was involved.

Since Pin1 has two domains that both bind pSer/Thr-Pro ligands, we set out to demonstrate selectivity for the WW domain over the catalytic domain of Pin1. Because both the catalytic and WW domain binding sites of Pin1 recognize the pSer/Thr–Pro motif, it was necessary to develop an assay specific for WW domain ligands. We previously developed a Pin1 WW domain-specific assay, called an Enzyme-Linked-Enzyme-Binding Assay (ELEBA) that was used to screen this small library of tetrapeptide ligands.^44^ In addition, ^15^N,^1^H HSQC NMR assays that are very specific in determining the binding site for Pin1 were used to determine location and affinities of binding in the WW domain. We now report the design, synthesis, and assay of a small combinatorial library targeted to the Pin1 WW domain, and the binding affinity and specificity of four hits from the library.

## Results and Discussion

### Synthesis of the Library

**Scheme 1:**
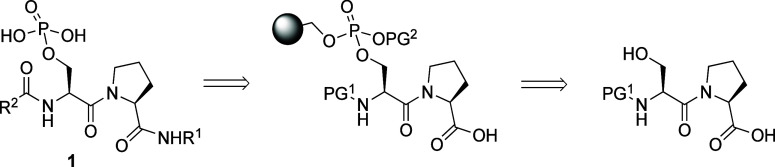
Designed Library and Its Retrosynthesis PG^1^ = Protecting Group

We designed a peptidomimetic library with pSer–Pro as the core, and a variety of unnatural amine and acid groups at *C*- and *N*-termini to screen for WW domain specific ligands (Scheme 1). We biased the amines and acids chosen based on the amino acid preferences in the natural substrates investigated by Verdecia, i.e. hydrophobic and aromatic acids at the *N*-terminus, and smaller cyclic and basic amines at the *C*-terminus.^9^ The library members are similar in size to tetrapeptides. The use of unnatural residues without exposed *N-* or *C*-termini is likely to prevent proteolytic degradation of hit compounds, particularly by exoproteases. In the future, hits could be further stabilized by replacement of the pSer–Pro moiety with our trans-locked mimic.^17,45^ We wanted to link the hydroxyl group of the pSer–Pro scaffold to the resin through a phosphate group to allow solid-phase coupling with amines and acids at the *C*- and *N*-termini, respectively (Scheme 1). Therefore, a solid-phase phosphorylating reagent (SPPR) was required.

**Scheme 2:**
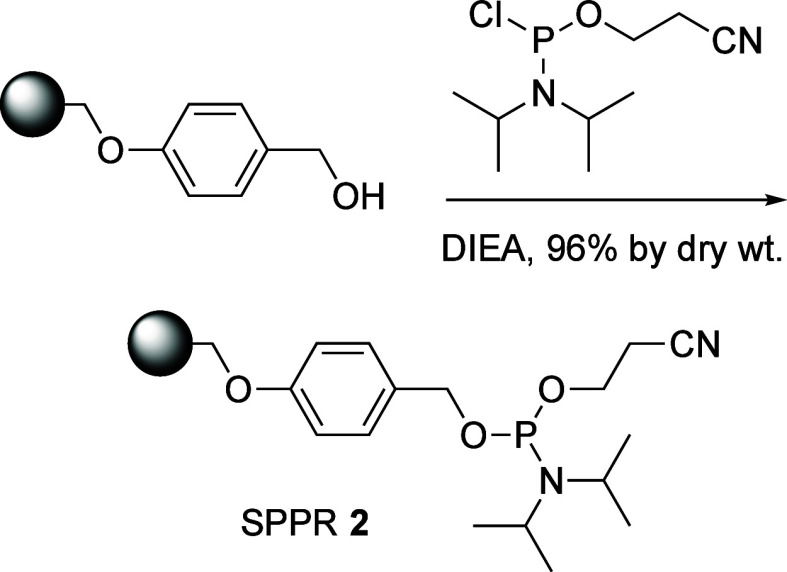
Synthesis of SPPR **2**

We developed SPPR **2** that is easily accessible in one step from commercially available Wang resin (Scheme 2). SPPR **2** was synthesized by mixing Wang polystyrene resin with commercially available 2-cyanoethyl *N*,*N*-diisopropyl chlorophosphoramidite in the presence of DIEA, with a yield of 96% by dry weight (Scheme 2). SPPR **2** was used in the same way as solution-phase phosphoramidite reagents.^40,41^

**Scheme 3:**
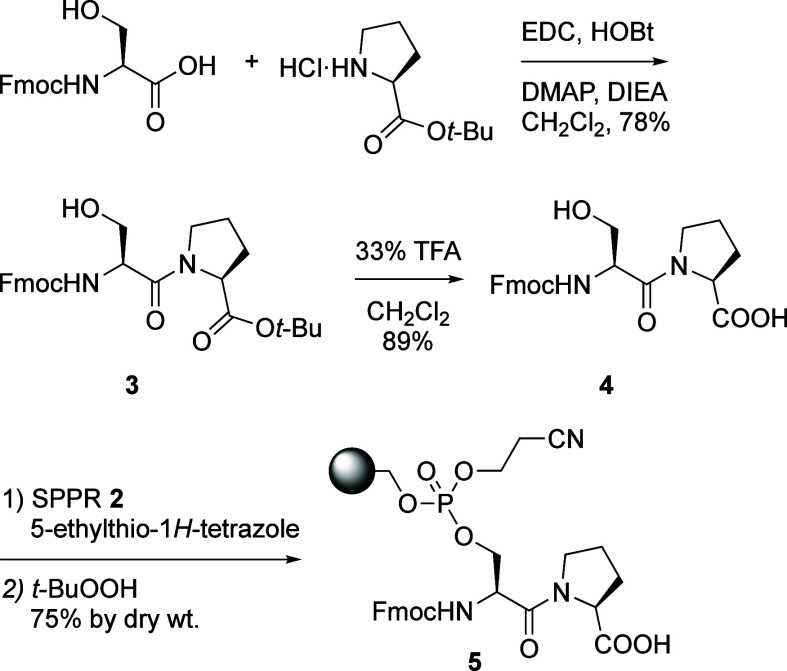
Synthesis and Phosphorylation of Fmoc–Ser–Pro–OH with SPPR **2**

Fmoc–Ser–Pro–OH **4** was synthesized in two steps as the core substrate for the designed library (Scheme 3). Commercially available Fmoc–Ser–OH and HCl·H–Pro–O*t*Bu were coupled with EDC/HOBt in solution to give Fmoc–Ser–Pro–O*t*Bu **3**, followed by deprotection of the *t*-butyl ester with TFA:CH_2_Cl_2_ 1:2 to afford dipeptide **4**. The Fmoc–Ser–Pro–OH **4** was attached to SPPR **2** using 5-ethylthiotetrazole, then the phosphite obtained was oxidized with *t*-BuOOH to give **5** (Scheme 3). The yield was determined to be 75% by dry weight.

**Figure 1:**
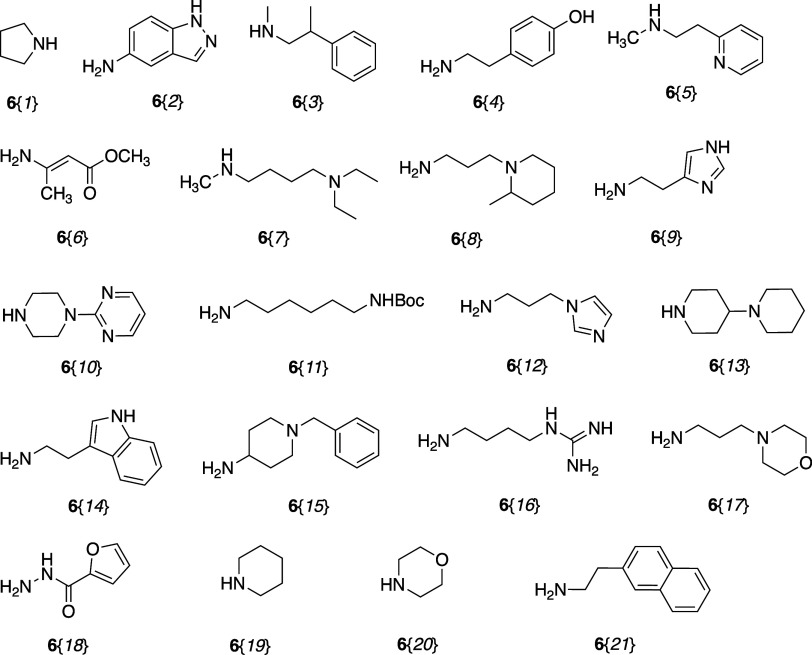
Reagent amines **6**{*1–21*}.

**Figure 2:**
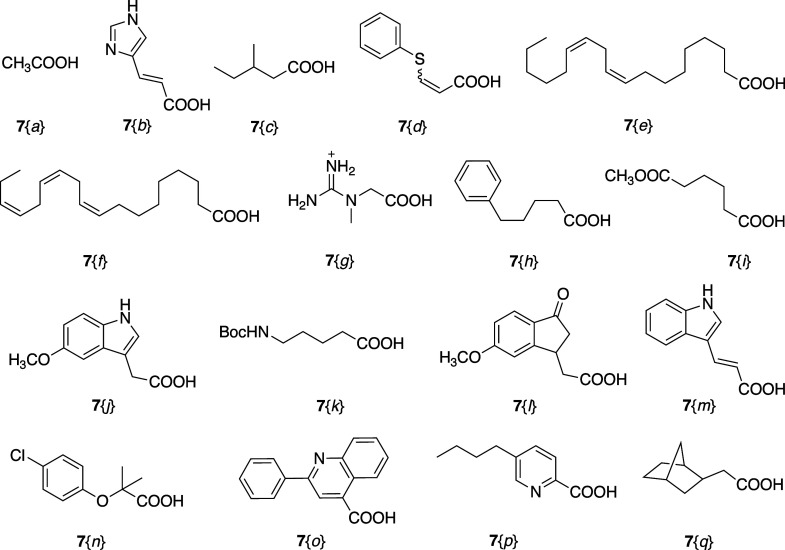
Reagent acids **7**{*a-q*}.

The library was designed with 21 amines at the *C*-terminus and 17 carboxylic acids at the *N*-terminus of the pSer–Pro core to give a combinatorial library of 357 ligands. Commercially available amines **6** (Figure 1) and carboxylic acids **7** (Figure 2) were chosen to build the library. The unnatural amines and carboxylic acids were selected with diverse structural and chemical properties to determine Pin1 WW domain binding preferences. At the *C*-terminal side of the pSer/Thr–Pro motif in peptides that bind to the WW-domain of Pin1, Val, Ser, and Pro are most commonly found, while Arg and Ile also appear.^9^ Thus, the amines were biased toward branched aliphatic primary amines, secondary cyclic amines, and basic side chains. Amino acids at the *N*-terminal side of the pSer/Thr–Pro motif in peptides that bind to the WW domain include: Arg, Thr, Gly, Ser, and Ala. Thus, in the set of acids (Figure 2), most are hydrophilic groups. Hydrophobic acids, such as **7**{*e*} and **7**{*f*} were also selected to increase the cell permeability. For chiral reagents, **6**{3}, **6**{8}, **7**{c}, **7**{*l*}, and **7**{q}, racemic mixtures were used for the library. The commercially available acid **7**{*d*} exists as a mixture of cis- and trans-isomers in a ratio of approximately 2.5:1, which was used without separation.

**Scheme 4:**
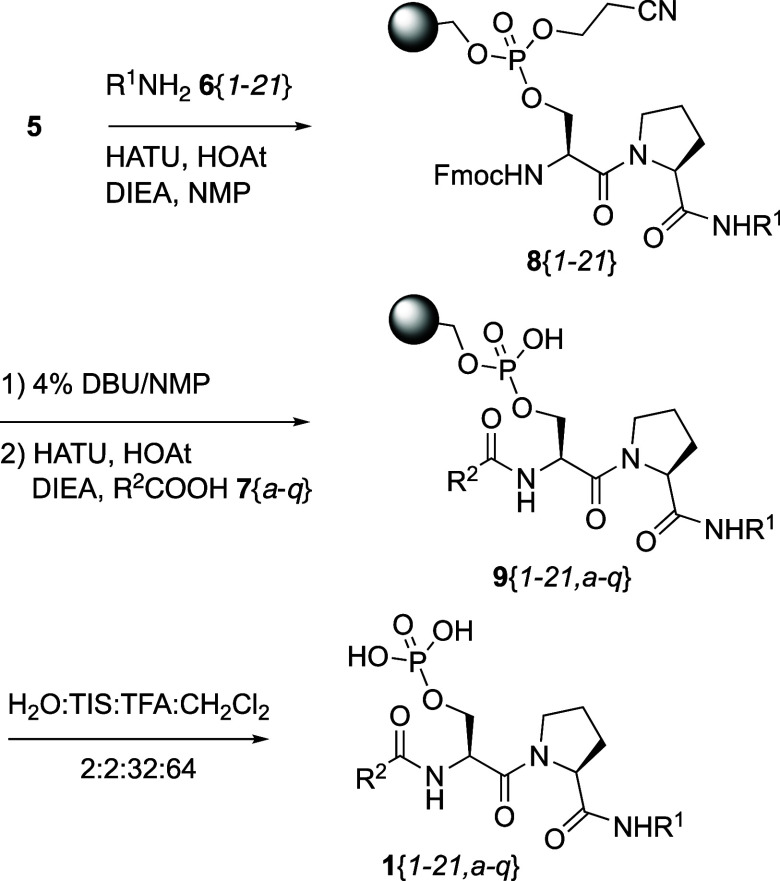
Synthesis of the Pin1 WW Domain Library

Library **1** was prepared with the dipeptide bound phospho-resin **5** in 96-well (8 × 12) plates on a Vanguard parallel synthesizer (Scheme 4). The free carboxylic acid of Fmoc–Ser(phosphoresin)–Pro–OH **5** was first coupled with amines **6**{*1–21*} using HATU/HOAt in the presence of DIEA. The two protecting groups, the 2-cyanoethyl of the SPPR and the *N*-terminal Fmoc of Ser, were removed simultaneously with 4% DBU in NMP (Scheme 4). Each of the deprotected amines on the resin was then coupled with 17 acids **7**{*a-q*}, using HATU/HOAt in the presence of DIEA to give the phosphorylated products attached to the resin. The final products **1** were cleaved from the resin with a mixture of 32% TFA in CH_2_Cl_2_ with 2% each H_2_O and triisopropylsilane (TIS) as cation scavengers to avoid side reactions with the aromatic groups in the cleavage step (Scheme 4). During TFA cleavage, the Boc protecting groups of **6**{11} and **7**{*k*}were also removed. After cleavage from the resin, the filtrates containing products were concentrated *in vacuo*, and cold diethyl ether was added to precipitate products. The mixtures were centrifuged, and the solutions of side-products were removed by decanting. The products were dried *in vacuo* and assayed without further purification.

The library as designed contained 357 members. However, the ^1^H NMR spectra of **1**{*1*,*m*}, **1**{*15*,*m*}, **1**{*1*,*o*}, and **1**{*15*,*o*} showed that two acids, **7**{*m*} and **7**{*o*}, did not undergo the coupling reactions with amines, probably due to low reactivity. Excluding these two groups, our library contained 315 (21 × 15) members. ^1^H NMR analysis of a sample of the library showed that compounds **1**{*1*,*a*}, **1**{*3*,*a*}, **1**{*6*,*b*}, **1**{*15*,*b*}, **1**{*17*,*b*}, **1**{*21*,*b*}, **1**{*13*,*c*}, **1**{*7*,*d*}, **1**{*4*,*e*}, **1**{*5*,*f*}, **1**{*1*,*h*}, **1**{*9*,*h*}, **1**{*10*,*h*}, **1**{*11*,*i*}, **1**{*12*,*l*}, **1**{*13*,*l*}, **1**{*15*,*n*}, **1**{*16*,*n*}, **1**{*20*,*n*}, **1**{*15*,*p*}, **1**{*17*,*p*} had estimated purity >85%, and compounds **1**{*2*,*b*}, **1**{*4*,*b*}, **1**{*7*,*k*} had estimated purity >50% (data not shown).

### Library Screening by ELEBA

**Figure 3:**
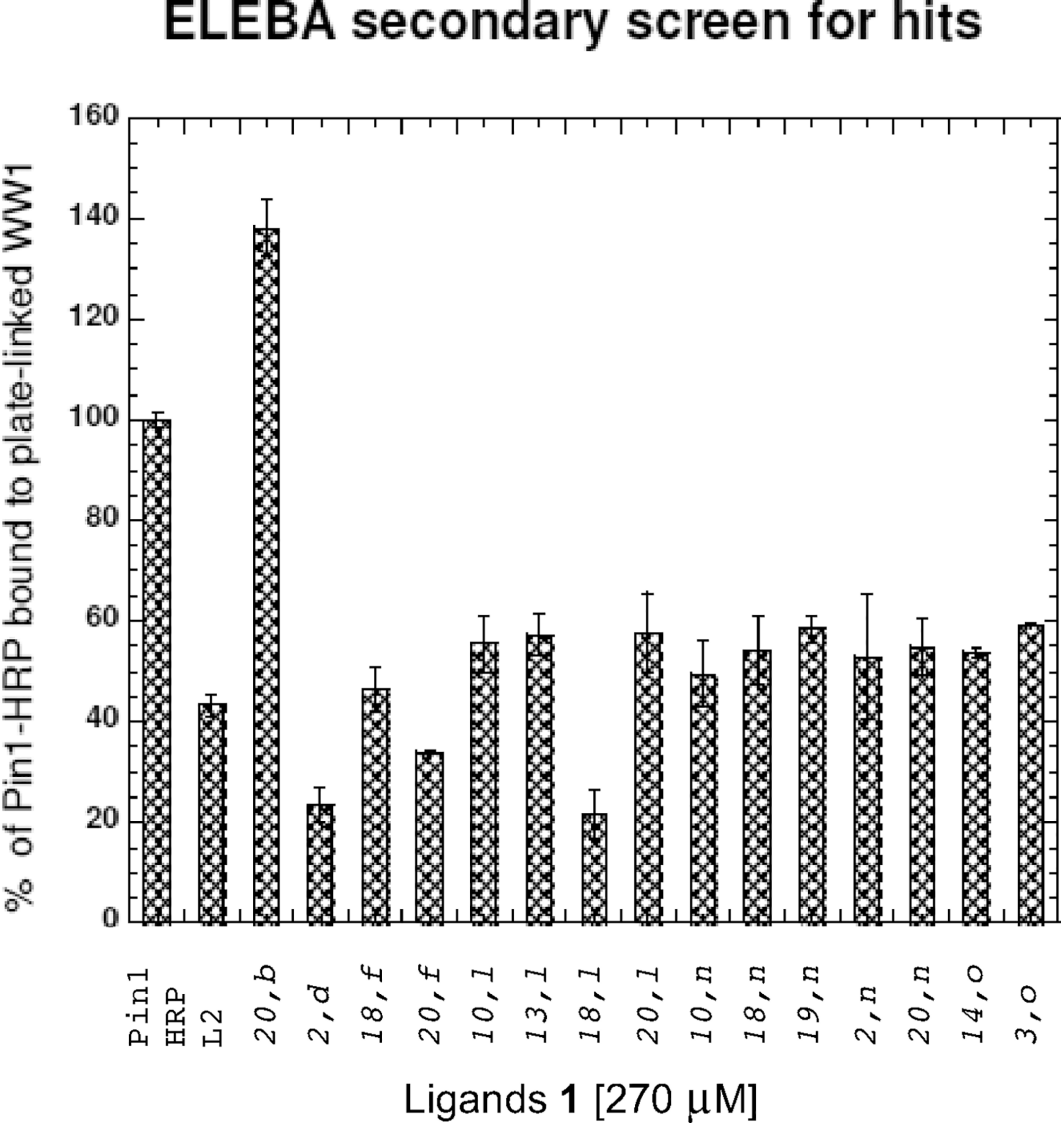
ELEBA secondary screen for the best compounds in the primary assay, plus compound **1**{*20,b*} with ca. 140% of baseline activity.

The library was screened by the ELEBA we reported previously.^44^ The crude products resulting from diethyl ether precipitation were used for the primary and secondary ELEBA without additional purification. The concentrations of the crude products were estimated based on quantitative yields; actual concentrations were thus lower, depending on yield and purity. The library products were dissolved in DMSO as stock solutions for assays. Fmoc–VPRpTPVGGGK–NH_2_**L1**, as the plate-linked ligand, has an affinity of 7.7 μM for the WW domain.^9^ Recombinant human Pin1 conjugated to horseradish peroxidase (Pin1–HRP) was used in the binding assays.^44^ Fmoc-VPRpTPVGGGK-NH_2_ L2 was used as the positive control and DMSO was used as the negative control on each plate. The percent of Pin1–HRP bound to the plate was calculated by normalizing the binding with DMSO to be 100%. In the primary assays (Figure S1), 14 of the best library members were identified: **1**{*2,d*}, **1**{*18,f*}, **1**{*20,f*}, **1**{*10, l*}, **1**{*13, l*}, **1**{*18, l*}, **1**{*20, l*}, **1**{*10, n*}, **1**{*18, n*}, **1**{*19, n*}, **1**{*2, n*}, **1**{*20, n*}, **1**{*14, o*}, **1**{*3, o*}. In the secondary assay, these ligands were measured again at 270 μM, and three hits, **1**{*2*, *d*}, **1**{*18*, *l*} and **1**{*2*, *n*}, were chosen for further analysis (Figure 3).

A few compounds showed enhanced binding of Pin1-HRP to the WW domain ligand on the plate, for example **1**{*20*, *b*} (Figure 3). Although this could indicate that **1**{*20, b*} is an agonist, we did not investigate this further. It is possible that some ligands bind in the PPIase catalytic site since all ligands contain the pSer–Pro motif, which is also recognized by the PPIase domain. If ligands are bound to the PPIase domain, the free WW domain may become more stably folded and bind more tightly to the plate-linked ligand **L1**. Verdecia et al. showed that full-length Pin1 binds phosphorylated ligands more tightly in the WW domain than the isolated PPIase or WW domains; these findings can be attributed to the folding stabilization of the whole protein.^9^

**Figure 4:**
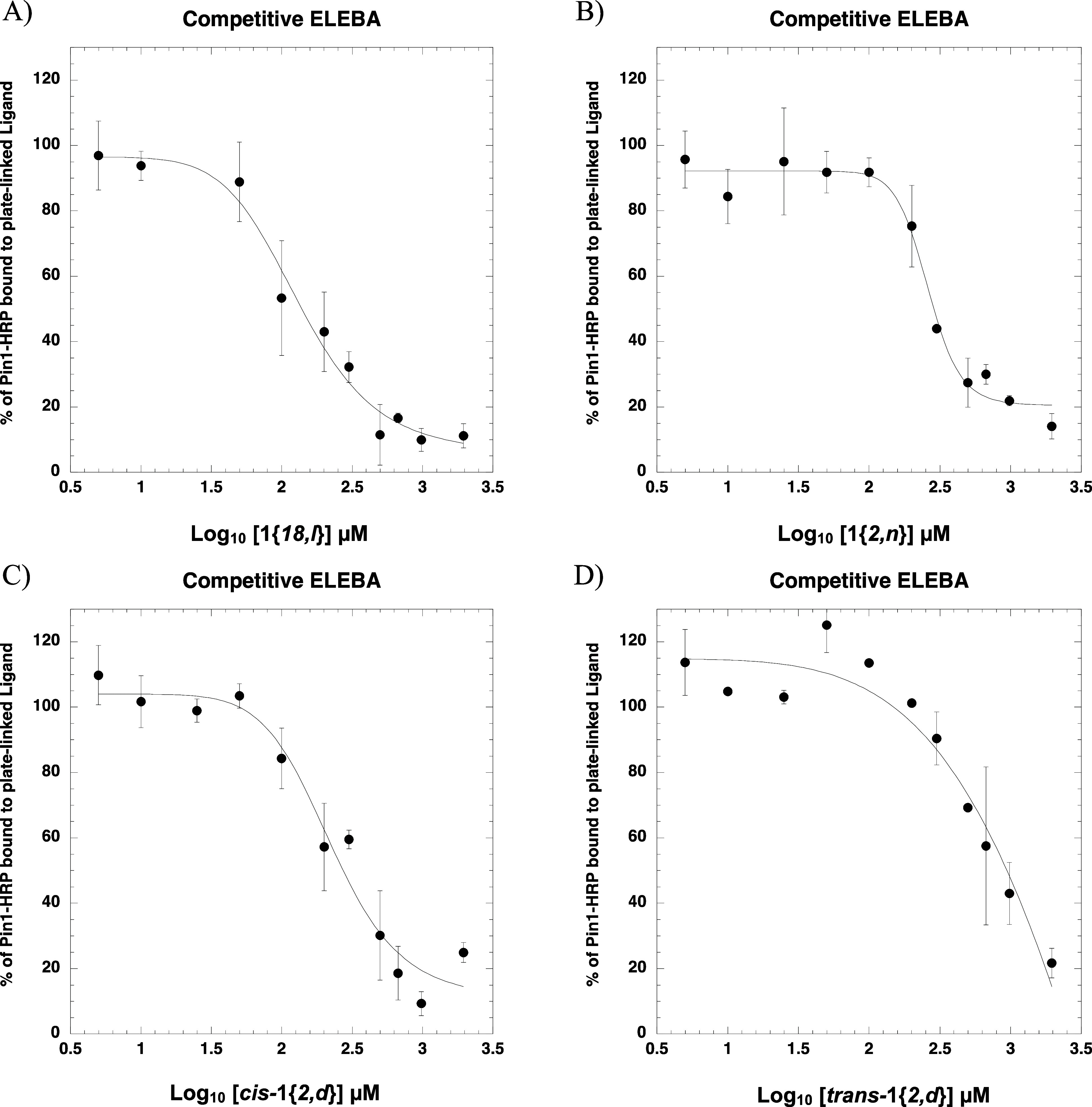
*K*_d-obs_ of hit compounds. Curves plotted as % Pin1-HRP bound vs Log_10_ ligand concentrations. (A) **1**{*18,l*}, (B) **1**{*2,n*}, (C) *cis*-**1**{*2*, *d*}, and (D) *trans*-**1**{*2*, *d*}.

Concentration-dependent ELEBAs were performed for ligands **1**{*2*, *d*}, **1**{*18*, *l*}, *cis*-**1**{*2*, *n*}, and *trans*-**1**{*2*, *n*} obtained by large-scale manual solid-phase synthesis and purification. The cis- and trans-isomers of **1**{*2*, *d*} were separated by HPLC. The two diastereomers (∼ 1:1) of **1**{*18*, *l*} were used as a mixture because they were inseparable in our systems. Competitive dissociation constants (*K*_d-obs_) were measured by ELEBA (Figure 4). *K*_d-obs_ values were normalized to percentage Pin1–HRP bound to the plate-linked ligand by setting the lowest concentration of competitive ligand in solution to 100% binding.^32^*K*_d-obs_ is defined as the concentration of a ligand where the % Pin1-HRP bound to the plate is 50%. The *K*_d-obs_ values of ligands **1**{*18*, *l*}, **1**{*2*, *n*}, *cis*-**1**{*2*, *d*}, and *trans*-**1**{*2*, *d*} are given in Table 1.

**Table 1:**
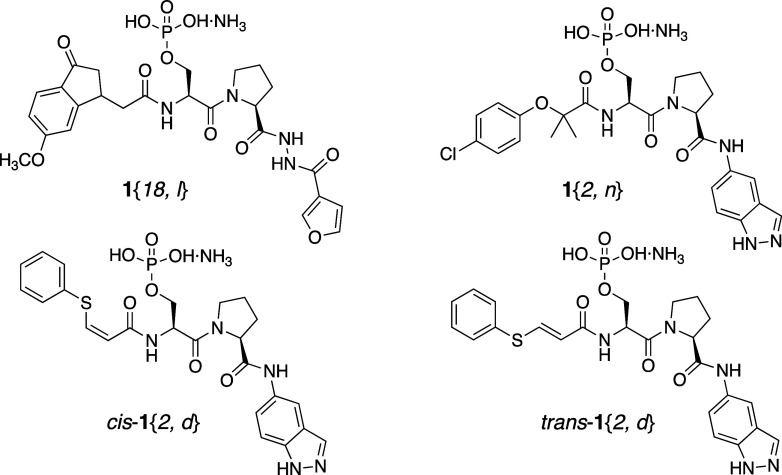
Hit Structures, ELEBA Competitive Dissociation Constants, and NMR HSQC Chemical Shift Perturbation (CSP) Titrations for Hit Compounds

Ligand	ELEBA *K*_d-obs_ (μM)	NMR CSP *K*_d_ (μM)
**1**{18, *l*}	130 ± 3	105 ± 9
**1**{2, *n*}	206 ± 3	208 ± 6
*cis*-**1**{2, *d*}	263 ± 6	95 ± 6
*trans*-**1**{2, *d*}	820 ± 6	214 ± 25

The best ligands had polar heteroaromatic rings (**6**{*2*} and **6**{*18*}) at the *C*-terminus (Table 1). All hit compounds from the primary assay had an aromatic acid (**7**{*b*}, **7**{*d*}, **7**{*l*}, and **7**{*n*}) at the *N*-terminus. This indicates a Pin1 WW domain binding preference for aromatic structures at both positions, which is surprising considering the preference for small, polar residues before and after pSerPro in native sequences that bind to the WW domain.^9^ The *cis*-**1**{*2*, *d*} isomer was about three times as potent as *trans*-**1**{*2*, *d*}, showing the importance of the geometry of the double bond. Acid **7**{*n*}, gave the most hits in the primary assays, which implied a general preference for this structure. However, the best ligand in the library identified by ELEBA was **1**{*18*, *l*}, indicating that the effects of the *C*- and *N*-terminal flanking structures are not simply additive (Table 1). We hypothesize that the WW domain binding-pocket could not simultaneously accommodate two large hydrophobic substituents at both ends of the pSer-Pro motif (Table 1). The polar aromatic acid and the polar hydrophobic amine might disturb the favorable pSer-*trans*-Pro conformation for binding to the WW domain.

Ligands containing the Ac group 7{a} were not competitive in the with the highly selective WW domain peptide ligand bound to the plate. Thus, the reported affinity of the parent Ac-pThr-Pro-NH_2_ ligand, that was measured by NMR for the isolated Pin1 WW domain, does not represent the affinity for the WW domain *in competition* with the catalytic domain, as does the ELEBA. The Pin1 WW domain has intradomain flexibility that is sensitive to the presence versus absence of the catalytic domain, and the WW domain folds to accommodate the ligands that bind.^49^ The ELEBA measures the affinity of the full-length Pin1 for a highly WW-domain-selective ligand bound to the plate, and thus represents a more biologically relevant assay.

### Modeling of Hit Compounds

**Figure 5:**
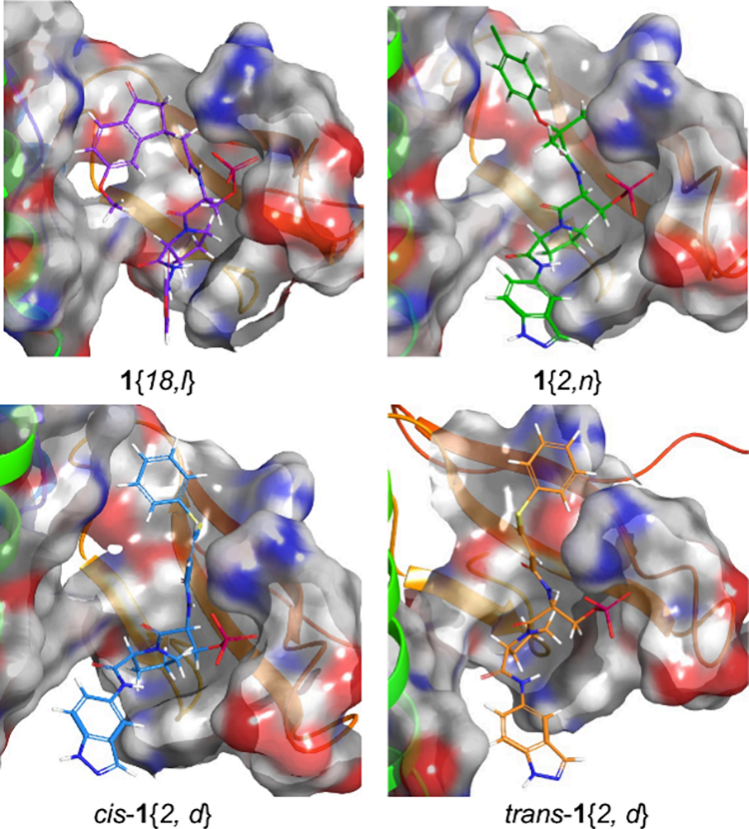
Models of ligands (sticks) bound to the WW domain of Pin1 (electrostatic surfaces) using Macromodel (Schrödinger, Inc.). (A) **1**{18, *l*} with carbons in purple, (B) **1**{2, *n*} in green, (C) *cis*-**1**{2, *d*} in light blue, and (D) *trans*-**1**{2, *d*} in orange.

To visualize binding, models of the four tightest-binding ligands in the Pin1 WW domain were created (Figure 5). The pSer–Pro residues of each ligand were mapped onto the pSer5-Pro6 of the *C*-terminal domain of RNA-polymerase II peptide ligand YpSPTpSPS (CTD-S2/S5) bound in the WW domain of Pin1 (PDB 1F8A).^9^ The ligand and Pin1 were minimized with OPLSe and continuous water solvation, and surfaces were calculated for the protein (Figure 5).

As expected, in each case the phosphate was bound in the pSer5 binding site, forming the same salt bridges with Pin1 WW Arg17, and hydrogen bonds with Ser16 and Tyr23, as in the crystal structure of YpSPTpSPS bound in the WW domain (Figure 5).^9^ The hydrophobic residues of each hit ligand fit into the WW domain binding channel, making contact with hydrophobic residues of the protein to differing extents. Compounds **1**{*18, l*} and *cis-***1**{*2, d*} appeared to make the best contacts, with the methoxyphenylcyclopentanone and phenyl groups folding back and filling the channel rather than extending out into solution (Figure 5). The phenyl rings of the other two ligands did not appear to fill the channel as well (Figure 5). On the contrary, the thiophenyl group of *trans*-**1**{*2, d*} appears to have a steric interaction with Arg17 that pulls the phosphate away from hydrogen bonding with Ser16 and Tyr23, and its thiophenyl ring does not make contact with the PPIase domain on the left, which may explain its weaker affinity (Figure 5).

### Binding Location and Affinities of Hits by NMR

**Figure 6:**
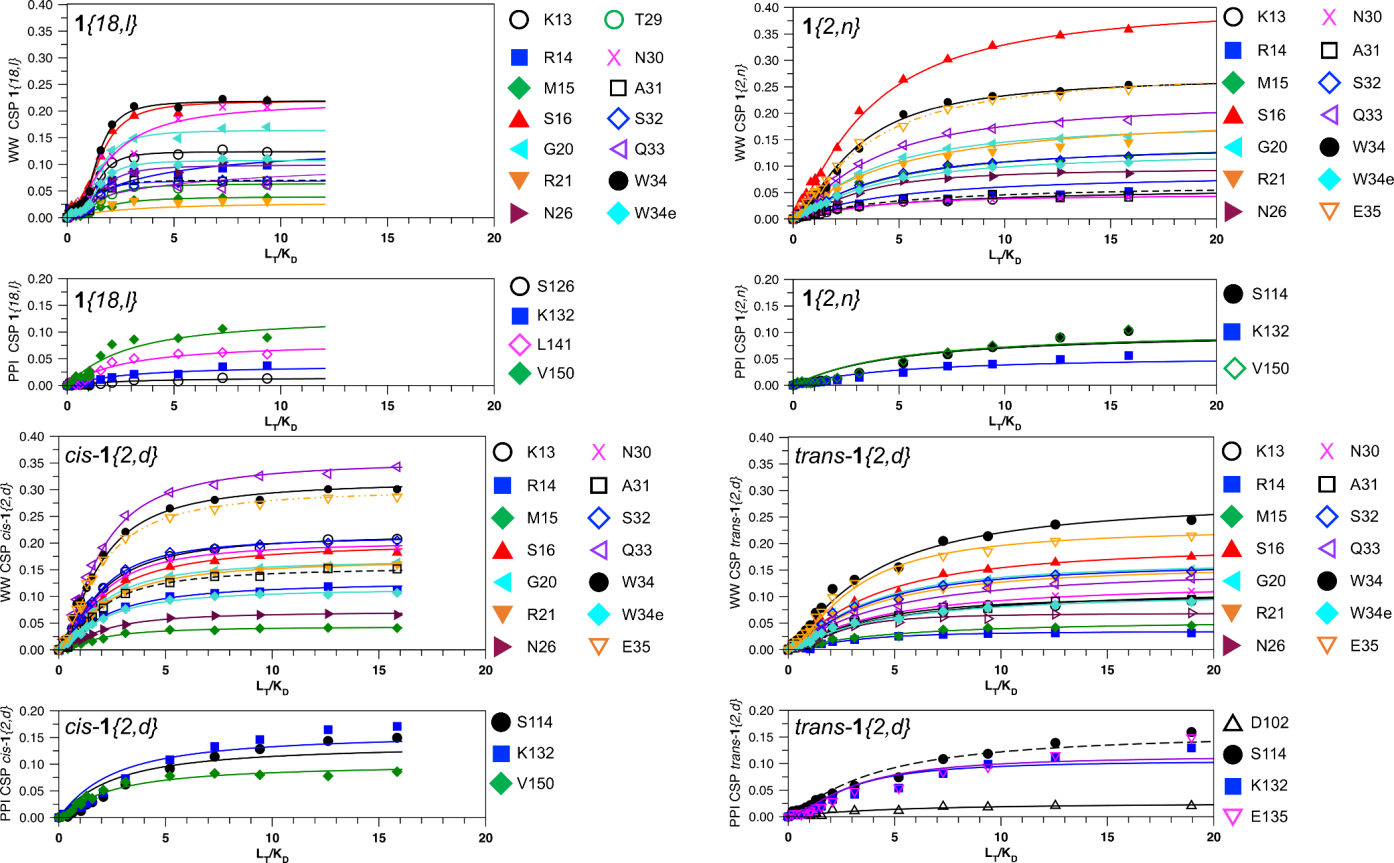
NMR titration of the Pin1 α-amino ^15^N HSQC CSPs in the WW domain and the PPIase domain plotted separately against total ligand concentration (L_T_) of **1**{*18, l*}, **1**{*2, n*}, *cis*-**1**{*2, d*}, and *trans*-**1**{*2, d*}, divided by *K*_d_.

The ELEBA was developed as a method for high-throughput screening, rather than as an absolute measure of binding affinity. The binding is competitive with the potent Pin1 ligand, Fmoc–VPRpTPVGGGK(plate)–NH_2_**L1** that is highly selective for the WW domain (4.9 μM for full-length Pin1, 7.7 μM for the WW domain, and no binding to the catalytic domain). Since **L1** is linked to the plate, ELEBA is not to be taken as a direct measurement of binding affinity.^32^ We undertook NMR measurements to determine the affinity and location of binding directly by NMR to distinguish between binding to the catalytic and WW domains of Pin1. Standard NMR backbone ^15^N–^1^H chemical shifts were mapped onto existing assignments of Pin1 by recording 2-D ^15^N–^1^H HSQC spectra of full-length ^15^N-Pin1 at different ligand concentrations (Figures S2–S5). NH cross-peaks showing ligand-induced chemical shift perturbations (CSPs) identified residues affected by binding. The largest magnitude CSPs were localized to the WW domain. Twelve residues from the WW domain (K13, R14, M15, S16, G20, R21, N30, A31, S32, Q33, W34, and E35) consistently displayed ligand-induced chemical shift perturbations (CSPs), confirming the location of binding for each of the four ligands (Figure 6).

Simultaneous fits of these CSPs to a two-state binding equilibrium^35,46^ gave K_*d*_ values indicating that the compounds were good ligands for the Pin1 WW domain (Table 1, Figure 6). The binding affinities of **1**{*18, l*} and **1**{*2, n*} were similar to those measured by ELEBA (Table 1). Ligand *cis-***1**{*2, d*}, rather than **1**{*18, l*}, showed the tightest binding by NMR, although the **1**{*18, l*} binding curves were sigmoidal (Figure 6). Compound *cis-***1**{*2, d*} still had higher affinity than *trans-***1**{*2, d*} by NMR, as in the ELEBA, although the absolute magnitudes were tighter by NMR (Table 1). The discrepancies between the *K*_d_ values measured by ELEBA and by NMR, particularly for *cis*- and *trans*-**1**{*2, d*}, are unsurprising for such very different assays.

We note that at the higher ligand concentrations, weak CSPs appeared for PPIase domain residues at the domain interface (L141, V150), or near the PPIase active site (C113, S114, S116).^46−48^ The domain interface CSPs are diagnostic of the decreased interdomain contact caused by ligands binding to the WW domain Loop I (residues 16–21).^49^ The weak CSPs in the PPIase active site showed linear increases at concentrations for which WW domain CSPs had already plateaued; the linear behavior indicates nonspecific binding, or a site of far weaker affinity compared to the WW domain. Both sets of Pin1 CSPs thus point to preferential binding to the WW domain, consistent with the ELEBA results.

### Modeling NMR Data

**Figure 7:**
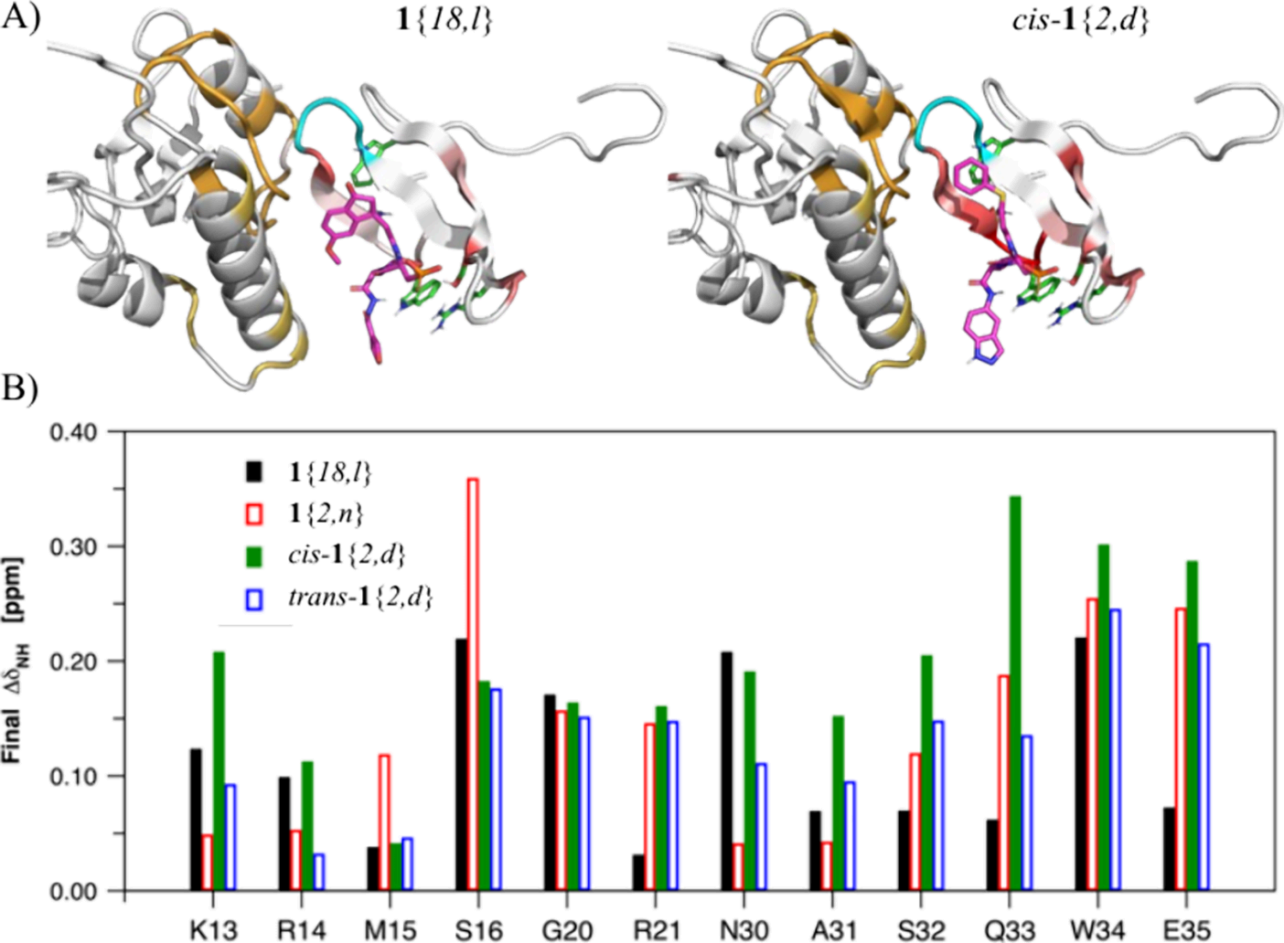
(A) Models of ligands **1**{*18, l*} and *cis*-**1**{*2, d*} (pink carbons) bound to Pin1, with W11, S16, R21, and W34 side chains (green carbons) using PyMol, show residues with CSPs on a gradient from highest to lowest, red to orange. (B) Composite CSP intensities (Δ*p*pm) at the highest concentrations for all four ligands: **1**{*18, l*}, **1**{*2, n*}, *cis*-**1**{*2, d*}, and *trans*-**1**{*2, d*} vs residues number for only interacting residues that are common among all four.

Pin1 residues interacting with ligands **1**{*18,l*} and *cis*-**1**{*2,d*} were colored on a gradient from red to orange based on the strength of the ^15^N CSP at the highest concentrations of ligand measured, representing mostly bound ligand (Figure 7). Residues in the WW domain showed the greatest CSPs in red, and residues in the PPIase domain at the interface showed somewhat weaker CSPs in orange, consistent with specific binding in the WW domain (Figure 7A). A plot of the composite CSPs vs all residues in common between the four ligand titrations shows consistent binding at the same residues in the WW domain (Figure 7B). The CSPs are stronger toward the *C*-terminal residues for ligand *cis*-**1**{*2,d*} than for the other three ligands, which indicates that the *cis*-thiophenyl group, *cis*-**7**{*d*}, fits best in the WW domain.

## Conclusions

SPPR **2** was successfully applied to synthesize our designed library with pSerPro in the core targeted to the Pin1 WW domain. Novel SPPR **2** was synthesized efficiently in one step. The synthesized library, **1**, was screened by ELEBA, and several hits were identified. Four hit compounds were resynthesized, and the competitive dissociation constants, *K*_d-obs_, were measured by ELEBA.^44^ The results showed that the Pin1 WW domain has a binding preference for aromatic structures at the *N*-terminus of its binding motif, and a binding preference for polar aromatic structures at the *C*-terminus. The *K*_d-obs_ measured by ELEBA is dependent on the binding affinity of the plate-linked ligand, and consequently is a relative value. Modeling of the four hit ligands docked into the WW domain help visualize the differences in binding affinity.

The location and binding affinities of the four hit ligands were also measured by HSQC NMR chemical shift perturbation. NMR titrations and data modeling showed that all four of the resynthesized hit compounds specifically bound in the WW domain, consistent with the ELEBA results. Modeling of the NMR data provides structural insights that could serve in the rational design of a specific tight-binding WW domain ligand. Such a ligand could block association of Pin1 with its physiological partner proteins separately from its PPIase activity.

## Experimental Section

### General Methods

The library synthesis was carried out on Vanguard Synthesizer from Advanced ChemTech. Polypropylene columns purchased from Thermo Scientific were used for manual solid-phase synthesis. Brine (NaCl) and NaHCO_3_ refer to saturated aqueous solutions, and HCl refers to 1N aqueous solution unless otherwise noted. Commercially available protected amino acids and reagents were used without further purification unless otherwise stated. Anhydrous peptide-synthesis grade NMP, and HPLC-grade CH_3_OH, CH_2_Cl_2_, and TFA were used in solid-phase syntheses. Anhydrous THF was distilled from Na-benzophenone, and CH_2_Cl_2_ was dried by passage through dry alumina. Dry DIEA was used directly from sealed bottles. *p*-Benzyloxy benzyl alcohol resin (Wang resin) was purchased from Novabiochem. Unless otherwise indicated, all reactions were carried out at ambient temperature. Flash chromatography was performed on 230–400 mesh ASTM silica gel with reagent grade solvents. The yield of SPPR **2** was based on the initial loading of the commercially available Wang resin, and the yields of library compounds **1** were based on the dry weight of resin **6**. NMR spectra were obtained at ambient temperature in CDCl_3_ unless otherwise noted. Proton, carbon-13, and phosphorus-31 NMR spectra were obtained at 500, 125, and 200 MHz, respectively, unless otherwise noted. In carbon-13 spectra, minor rotamer chemical shifts, if applicable, are indicated by (m). Coupling constants, *J*, are given in Hz. Optical rotations are reported as concentration in g/100 mL and solvent in parentheses. Preparative HPLC were obtained on a Xbridge C18 19 × 100 mm column with a 0% to 100% CH_3_CN/H_2_O gradient over 12 min, and 100% CH_3_CN for 4 min, flow rate 12 mL/min, λ = 254 nm, with exceptions noted. The HRMS analyses were carried out on Agilent Technologies 6220 Accurate-Mass TOF LC/MS. The MS ionization methods were ESI^–^, unless otherwise noted. Pin1–HRP was purchased from R&D Systems. Microtiter plates were purchased from Corning Life Sciences.

### Solid-Phase Phosphorylating Resin (SPPR) **2**

In a one-necked flask, p-benzyloxy benzyl alcohol resin (3.80 g, batch No. A11085, loading: 0.72 mmol/g, 2.74 mmol,) was dried under high vacuum for 0.5 h, and the flask was filled with dry N_2_. Dry THF (120 mL) was added into the flask, and the mixture was shaken for 15 min to swell the resin. Dry DIEA (1.1 g, 8.1 mmol) was added, immediately followed by addition of 2-cyanoethyl *N*,*N*-diisopropyl chlorophosphoramidite (1.9 g, 8.1 mmol). The mixture was shaken on an orbital shaker for 3 h at room temperature. The mixture was filtered and washed with THF (2 × 120 mL) and CH_2_Cl_2_ (2 × 120 mL). The resin was dried under high vacuum overnight to give 4.33 g of SPPR **2** with a yield of 96% by weight. A small amount of resin (∼25 mg) was cleaved with 2 mL of TFA/CH_2_Cl_2_ (1/2, v/v) for 45 min. The mixture was filtered, and the filtrate was concentrated to give the mixture of 2-cyanoethyl phosphonate and diisopropyl amine TFA salt with a ratio of 1:1. ^1^H NMR (400 MHz): δ 8.89 (br s, 2H), 4.3 (br, 2H), 4.12 (dt, *J* = 6.3, 8.7, 2H), 3.33 (sept, *J* = 6.5, 2H), 2.72 (dt, *J* = 6.3, 8.7, 2H) 1.35 (d, *J* = 6.5, 12H).

### Fmoc–Ser–Pro–O*t*Bu, **3**

To a one-necked flask containing CH_2_Cl_2_ (250 mL), Fmoc–Ser–OH (2.8 g, 8.5 mmol) and HCl·H–Pro–*t*Bu (1.8 g, 8.7 mmol) was added. The mixture was stirred for 5 min. EDC (2.5 g, 12.8 mmol), HOBt (1.96 g, 12.8 mmol), DMAP (∼50 mg), and DIEA (3.3 g, 25.5 mmol) were added and the mixture was stirred for 22 h. The resulting solution was washed with 1 N HCl (2 × 160 mL), NaHCO_3_ (2 × 160 mL), H_2_O (2 × 160 mL), and brine (160 mL). The organic solution was dried with Na_2_SO_4_ and evaporated. The residue was purified by recrystallization (CH_2_Cl_2_/hexanes 1/8, v/v, 250 mL) to give 3.2 g of **3** as a white solid with a yield of 78%. ^1^H NMR: δ 7.75 (d, *J* = 7.5, 2H), 7.58 (d, *J* = 7.1, 2H), 7.39 (t, *J* = 7.4, 2H), 7.30 (dt, *J* = 0.8, 7.4, 2H), 5.91 (d, *J* = 8.2, 1H), 4.70 (q, *J* = 6.3, 0.85H), 4.61 (m, 0.15H), 4.48 (dd, *J* = 2, 8.7, 1H), 4.36 (d, *J* = 7.2, 2H), 4.20 (t, *J* = 7.2, 1H), 3.94 (m, 0.85H), 3.82 (m, 0.85H), 3.75 (m, 0.85H), 3.69 (m, 1H), 3.56 (t, *J* = 7.2, 1H), 3.45 (m, 0.15H), 3.38 (m, 0.15H), 3.18 (m, 0.15H), 2.22 (m, 1H), 1.97 (m, 3H), 1.47 (s, 7.65H), 1.42 (s, 1.35H). ^13^C NMR: δ 171.8, 169.8, 156.2, 143.9, 141.4, 127.8, 127.2, 125.2, 120.1, 82.5, 67.3, 62, 60.0, 50, 47.5, 47.2, 29.0, 28.0, 27.9 (m), 24.9.

### Fmoc–Ser–Pro–OH, **4**

(Fmoc–Ser–Pro–OH, **4**, was previously synthesized by the Raushel group as a mixture of 19 Fmoc–Xaa–Pro–OH dipeptides bound to Wang resin at the *C*-terminus without cleavage.^50^) In a one-necked flask, Fmoc–Ser–Pro–O*t*Bu, **3**, (2.8 g, 5.8 mmol) was dissolved in CH_2_Cl_2_ (80 mL). TFA (40 mL) was added, and the mixture was stirred for 1.5 h. The solution was concentrated, and the resulting yellow oil was purified with flash chromatography (EtOAc:hexanes 1:1 with 3% acetic acid, then EtOAc with 3% acetic acid) to give 2.2 g (yield 89%) of **4** as a white foam. ^1^H NMR (DMSO): δ 7.88 (d, *J* = 7.5, 2H), 7.73 (t, *J* = 6.0, 2H), 7.60 (d, *J* = 7.9, 0.8H), 7.41 (t, *J* = 7.4, 2H), 7.32 (t, *J* = 7.0, 2H), 7.25 (d, *J* = 8.5, 0.2H), 4.74 (d, *J* = 8.1, 0.2H), 4.38 (q, *J* = 6.8, 0.8H), 4.34–4.20 (m, 3.8H), 4.12 (br, 0.2H), 3.66 (m, 2H), 3.55–3.37 (m, 1.8H), 3.14 (m, 0.2H), 2.16–2.07 (m, 1H), 1.92–1.78 (m, 3H). ^13^C NMR (DMSO): δ 173.5 (m), 173.4, 169.4 (m), 169.0, 156.2, 155.4 (m), 143.9, 140.8, 127.7, 127.1, 125.6 (m), 125.4, 120.1, 65.9 (m), 65.7, 62.6 (m), 61.1, 58.6, 55.4, 54 (m), 46.7, 46.6, 46.1 (m), 30.8 (m), 28.6, 25, 21.9 (m).

### Fmoc-Ser-Pro-OH Bound Resin, **5**

To a dry flask, SPPR **2** (1.53 g, 0.964 mmol) and THF (80 mL) were added. A solution of Fmoc–Ser–Pro–OH **4** (1.02 g, 2.41 mmol) in THF (40 mL) was dried with activated 4 Å molecular sieves for 2h, then transferred into a flask containing dry **2**. 5-(Ethylthio)-1*H*-tetrazole (0.314 g, 2.41 mmol) was added in one portion, and the mixture was shaken for 26 h. The mixture was then cooled with a dry ice/acetone bath. A solution of *t*-butyl peroxide in decane (5.0–6.0 M, 1.54 mL, 7.7–9.2 mmol) was added. The dry ice/acetone bath was removed, and the mixture was shaken for 45 min. The resin was collected by filtration, then washed with NMP (120 mL x 3), and CH_2_Cl_2_ (120 mL x 3). The resin was dried under high vacuum to give 1.76 g (74% based on weight) of dipeptide bound resin **6**. A small sample was cleaved to give **5a**, which was characterized with ^1^H NMR. ^1^H NMR: δ 7.74 (d, *J* = 7.6, 2H), 7.58 (t, *J* = 7.6, 2H), 7.37 (t, *J* = 7.3, 2H), 7.27 (m, 2H), 4.83–4.17 (m, 12H), 3.79 (br, 1H), 3.72 (br, 1H), 2.70 (br, 2H), 2.17 (br, 1H), 2.07 (br, 2H), 1.93 (br, 1H).

### General Procedure to Synthesize **8**{*1*-*21*}

Fmoc–Ser(phosphoresin)–Pro–OH **5** (2.77g, 1.02 mmol) was suspended into a mixed solvent, NMP:CH_2_Cl_2_ 4:1 v/v (117 mL). The mixture was very gently stirred. With an automatic pipet, the suspended mixture containing the resin (0.5 mL) was dispensed into each well of the reaction block. Then reaction wells were drained, and thus each well contained 12 mg of resin **5** (∼0.0044 mmol). An NMP solution (0.9 mL) containing HATU (9 mg, 0.013 mmol), HOAt (1.7 mg, 0.013 mmol) and DIEA (3.3 mg, 0.026 mmol) was added into each reaction vessel, followed by addition of amines **6**{*1–21*}. The mixture was shaken for 15 min. The reaction vessels were emptied, and the remaining resin was washed with NMP twice. The coupling reaction with amines **6**{*1–21*} was repeated, and the resin was washed with NMP (2 × 1 mL) and CH_2_Cl_2_ (2 × 1 mL).

### General Procedure to Synthesize **1**{*1*-*21*, *a*-*q*}

A solution of 4% DBU in NMP (0.8 mL) was added into each well containing the Fmoc–Ser(phosphoresin)–Pro–NHR^1^**8**{*1–21*}. The reaction block was shaken for 5 min, emptied, and then washed with NMP (2 × 1 mL). The deprotection procedure was repeated for 15 min, and the reaction block was washed with NMP (2 × 1 mL) and CH_2_Cl_2_ (2 × 1 mL) to give deprotected H-Ser(phosphoresin)–Pro–NHR^1^. HATU (0.23 g, 0.61 mmol), HOAt (83 mg, 0.61 mmol), and DIEA (0.16 g, 1.2 mmol) were added into NMP (7.2 mL). To each solution, an acid **7**{*a–q*} (0.61 mmol) was added, then allowed to stand for 15 min to make the activated ester solutions. NMP (0.75 mL) was dispensed into each well containing H–Ser(phosphoresin)–Pro–NHR^1^, followed by the addition of the activated ester solutions (0.15 mL) prepared above. The reaction block was shaken for 15 min, emptied, and washed with NMP (2 × 1 mL). The coupling reaction procedure was repeated, and the reaction block was washed with NMP (2 × 1 mL), CH_2_Cl_2_ (2 × 1 mL), CH_3_OH (2 × 1 mL), and CH_2_Cl_2_ (2 × 1 mL) to give resin **9**{1-21, *a*-*q*}. A mixture of H_2_O:TIS:TFA:CH_2_Cl_2_ 2:2:32:64 (0.8 mL) was added into each well containing resin **9**{1-21, *a*-*q*}. The reaction block was shaken for 45 min. The heating/cooling module was replaced with the cleavage block. The reaction block was emptied, and the filtrate was collected into the cleavage block. The filtrate was concentrated to give a white to yellow oil. Each vial in the cleavage block contains a product **1**{1-21, *a*-*q*}. CH_3_OH (∼80 μL) was added to the crude product, followed by slow addition of diethyl ether (∼2 mL). The vial was swirled while ether was added. The mixture was centrifuged, and the ether was decanted. The residue was washed with diethyl ether, and dried. DMSO (0.12 mL) was added into the vial to make the stock solution for ELEBA. The yields of the library were assumed to be quantitative, and consequently the concentrations of the stock solutions were 37 mM.

### Data for Individual Library Members

Large-scale synthesis of *cis*- and *trans*-**1**{2,*d*}, **1**{2, *n*}, and **1**{18, *l*} were performed in a similar way to the library, except in a polypropylene tube, from commercially available acids and amines.

### Compound **1**{*2*, *d*}

Amine **6**{2} and acid **7**{*d*}, as a mixture of cis- and trans-isomers, in a ratio of approximately 2.5:1, were coupled. The crude product was obtained as a mixture of cis- and trans-isomers in a ratio of about 1:1.4, indicating trans-**7**{*d*} was more reactive than the cis-acid in the coupling reaction. The ratio was determined by the NMR of the crude product in CD_3_OD. In the spectrum, both cis- and trans-isomers exist as two rotamers. The alkene proton of cis-isomer, δ 6.10 (d, *J* = 10.0, 0.75H), 6.00 (d, *J* = 11.2, 0.25H); the alkene proton of trans-isomer, δ 5.90 (d, *J* = 18.0, 1.2H), 5.78 (d, *J* = 15.0, 0.2H). Yield of crude product: 25 mg, 96%. The crude product (21 mg) was purified by preparative HPLC with a 0% to 100% CH_3_OH/H_2_O containing 20 mM NH_4_OAc gradient over 21 min, flow rate 15 mL/min, λ = 254 nm. Retention times: cis-**1**{2,*d*} at 12.3 min, trans-**1**{2,*d*} at 12.8 min.

*Cis*-**1**{2, *d*}, purified as monoammonium salt gave 5.6 mg, 25% yield. ^1^H NMR (DMSO): δ 12.95 (br, 0.8H), 10.78 (br, 0.2H), 9.92 (s, 0.8H), 8.76 (br, 0.8H), 8.45 (br, 0.2H), 8.17 (s, 0.2H), 8.15 (s, 0.8H), 7.99 (s, 1H), 7.90 (s, 0.2H), 7.62 (d, *J* = 8.0, 0.2H), 7.51–7.30 (m, 6.8H), 7.19 (d, *J* = 9.7, 0.8H), 7.10 (d, *J* = 9.2, 0.2H), 6.15 (m, 1H), 5.04 (br, 0.2H), 4.83 (m, 1H), 4.45 (dd, *J* = 0, 8.4, 0.8H), 4.43 (m, 0.2H), 4.08 (br, 0.8H), 3.84 (m, 2.4H), 3.48 (m, 0.6H), 2.23–2.15 (m, 1H), 2.02–1.84 (m, 3H). ^13^C NMR (DMSO): δ 170.1, 168.4, 165.3, 142.2, 137.0, 133.4, 132.0, 129.6, 129.4, 127.4, 122.6, 121.6, 120.6, 116.6, 110.02, 109.95, 63.6, 60.6, 52.3 (d, *J* = 6.1), 47.2, 46.7 (m), 29.6, 26. ^31^P NMR (DMSO): δ 1.07, 0.46 (m). H_3_PO_4_ was used as the external standard and was referenced with a chemical shift of 0.0 ppm. The chemical shift of the peak for the major rotamer was 1.07, while the peak for the minor rotamer overlapped with the broad peak for H_3_PO_4_. To show the chemical shift of the minor rotamer, in the spectrum without an external standard, the major peak was reference as 1.07 ppm, and the chemical shift of the minor peak was 0.46 ppm. HRMS calcd. for C_24_H_25_N_5_O_7_PS (M-H^–^) *m*/*z* = 558.1218, found *m*/*z* = 558.1173. [α]^22^_D_ = −69.4 (*c* 0.657, DMSO).

*Trans*-**1**{2, *d*}, purified as monoammonium salt 6.8 mg, 30% yield. ^1^H NMR (DMSO): δ 12.91 (br, 0.8H), 10.70 (br, 0.2H), 9.88 (s, 0.8H), 8.59 (m, 0.8H), 8.26 (d, *J* = 7.0, 0.2H), 8.12 (s, 0.8H), 8.09 (s, 0.2H), 7.98 (s, 1H), 7.95 (s, 0.2H), 7.53–7.32 (m, 8H), 5.98 (d, *J* = 15.0, 1H), 4.97 (m, 0.2H), 4.84 (m, 1H), 4.40 (dd, *J* = 3.9, 8.4, 1H), 4.04 (m, 0.8H), 3.86–3.74 (m, 2.6H), 2.23–1.82 (m, 4H). ^13^C NMR (DMSO): δ 170.0, 168.2, 163.5, 163.0 (m), 139.6, 139.3 (m), 137.0, 133.3, 132.0, 131.8 (m), 131.0 (m), 130.9, 129.9, 129.7, 128.8, 128.6 (m), 122.6, 121.0 (m), 120.6, 119.7 (m), 119.6, 110.4 (m), 110.0, 109.9, 109.8 (m), 63.7, 60.6, 52.3 (d, *J* = 3.1), 47.1, 46.7 (m), 29.6, 25. ^31^P NMR (DMSO): 0.97, 0.32 (m). HRMS calcd. for C_24_H_25_N_5_O_7_PS (M-H^–^) *m*/*z* = 558.1218, found *m*/*z* = 558.1171. [α]^22^_D_ = −69.5 (*c* 0.783, DMSO).

### Compound **1**{*2*, *n*}

Amine **6**{2} and acid **7**{*o*} were coupled. Yield of crude product: 47 mg, 85%. The crude product was purified by preparative HPLC with 1% HCO_2_H added in the mobile phases, 32 mg, 58% yield. ^1^H NMR (DMSO): δ 10.42 (s, 0.15H), 9.94 (s, 0.85H), 8.44 (d, *J* = 7.3, 0.85H), 8.11 (s, 0.85H), 8.09 (s, 0.15H), 8.00 (s, 0.85H), 7.97 (s, 0.15H), 7.80 (d, *J* = 7.3, 0.15H), 7.45 (m, 2H), 7.29 (m, 2H), 6.99 (m, 2H), 4.92 (d, *J* = 6.9, 0.15H), 4.86 (m, 0.85H), 4.74 (q, *J* = 6.6, 0.15H), 4.46 (dd, *J* = 2, 8.4, 0.85H), 4.16 (m, 0.85H), 4.04 (m, 1H), 3.94 (q, *J* = 8.5, 0.15H), 3.74 (m, 1.7H), 3.51 (m, 0.3H), 2.31–2.16 (m, 1H), 2.03–1.85 (m, 3H), 1.42 (s, 2.6H), 1.41 (s, 2.6H), 1.36 (s, 0.4H), 1.30 (s, 0.4H). ^13^C NMR (DMSO): δ 173.4, 170.1, 167.4, 153.6, 137.0, 133.3, 131.9, 129.0, 126.6, 123.1, 122.8, 120.4, 110.1, 109.9, 80.8, 63.6, 60.6, 52.4, 47.0, 29.5, 25.0, 28, 26. ^31^P NMR (DMSO): δ 0.502, 0.118 (m). HRMS calcd. for C_25_H_28_ ClN_5_O_8_P (M-H^–^) *m*/*z* = 592.1370, found *m*/*z* = 592.1345. [α]^22^_D_ = −40.1 (*c* 0.551, CH_3_OH).

### Compound **1**{*18*, *l*}

Amine **6**{18} and racemic acid **7**{*l*} were coupled. Compound **1**{18, *l*} exists as a pair of diastereomers, in a ratio of ∼1:1 measured by ^1^H NMR. ^1^H NMR also showed the presence of rotamers. Yield of crude product: 73 mg, 89%. The crude product (32 mg) was purified by preparative HPLC with a 0% to 67% CH_3_CN/H_2_O with 1% HCO_2_H gradient over 14 min, and 100% CH_3_CN for 2 min. The two diastereomers were not separated, 17 mg, 47% yield. ^1^H NMR (CD_3_OD): δ 7.77 (m, 0.12H), 7.70 (s, 0.88H), 7.61 (d, *J* = 8.5, 0.75H), 7.59 (m, 0.25H), 7.27 (m, 0.12H), 7.19 (d, *J* = 3.2, 0.88H), 7.11 (m, 1H), 6.97 (d, *J* = 8.5, 0.75H), 6.94 (m, 0.25H), 6.64 (m, 0.12H), 6.60 (m, 0.88H), 4.99 (br, 1H), 4.93 (m, 1H), 4.52 (m, 0.75H), 4.26 (br, 0.75H), 4.11 (m, 1.25H), 3.92–3.75 (m, 6.75H), 3.65 (m, 0.25H), 3.57 (m, 0.25H), 2.85 (m, 1H), 2.52–1.98 (m, 5H). ^13^C NMR (CD_3_OD): δ 206.7, 170, 173.85 (m), 173.76, 170.5 (m), 170.0, 169.9, 167.6, 167.5, 162.5, 162.4, 159.6, 147.4, 147.0, 130.8, 130.7, 126.1, 117.4 (m), 117.24, 117.21, 116.8 (m), 116.6, 113.1 (m), 113.0, 110.2, 110.1, 110.0 (m), 66.6 (m), 65.9, 65.8, 60.92 (m), 60.86 (m), 60.65, 60.58, 56.51, 56.46, 53.5, 53.3, 43.7, 42.2, 42.0, 36.3, 36.2 (m), 36.14, 36.06 (m), 32.8 (m), 32.7 (m), 30.7, 25.91, 25.87, 23.2. ^31^P NMR (CD_3_OD): δ 0.00 ppm (Only one peak was observed in either the ^31^P spectrum of **1**{18, *l*} with or without H_3_PO_4_ as the external standard, which implies that the peak for **1**{18, *l*} overlapped with H_3_PO_4_). HRMS calcd. for C_25_H_28_N_4_O_11_P (M-H^–^) *m*/*z* = 591.1498, found *m*/*z* = 591.1522. [α]^22^_D_ = −95.6 (*c* 0.884, CH_3_OH).

### Assay of the Library by ELEBA

#### Primary Screening Assays

The Pin1 WW domain ligand, Fmoc–VPRpTPVGGGK–NH_2_, (100 μL/well, 245 μM stock solution) was covalently attached to a 96-well plate through the ε-amino group of the Lys side chain at pH 11.0, as published.^44^ Ligands were dissolved in DMSO at stock concentrations of ca. 50 mM by weight. Competitive binding with plate-linked Fmoc–VPRpTPVGGGK–NH_2_**L1** of pSer–Pro dipeptides was measured at ca. 600 μM for primary screening. In the primary assays, all library members were screened with the crude products. **1**{1-21, *d*} were measured as mixtures of cis- and trans-isomers. Library members synthesized from racemic reagents, such as **1**{1-21, *c*}, **1**{1-21, *l*}, **1**{1-21, *q*}, **1**{3, *a*-*q*} and **1**{8, *a*-*q*} were measured as diastereomeric mixtures. The library members were dissolved in DMSO as stock solutions with a concentration of about 37 mM based on estimated yields. The DMSO stock solution of each compound (4 μL) was added into a solution of recombinant Pin1–HRP (R&D Systems) in a PBS buffer with 2% BSA (pH = 7.3). DMSO (4 μL) was used as a negative control. The final volume of the solution was 250 μL, the final concentration of Pin1–HRP was 200 pM, and the estimated final compound concentration was 590 μM. The resulting solution was preincubated for 30 min at ambient temperature, and was added into two wells (100 μL each, for assay in duplicate) of the ligand-linked plate. The solutions were incubated for another 1 h. The solutions were removed by inverting and shaking the plate, and the plate was washed with buffer (10 nM imidazole, and 0.05% Tween 20 in PBS at pH 7.3) three times. A 1:1 solution of hydrogen peroxide and 3,3,5,5′-tetramethylbenzidine (100 μL) was added into each plate well, and incubated in the dark for 20 min. A 2N H_2_SO_4_ solution (50 μL) was added to each well to quench the reaction, and the absorbance of was determined at 450 nm. The percentage of Pin1–HRP bound to the plate (% binding) was calculated by eq 1. To calculate the absorbance of a plate well in eq 1, the blank ligand-linked plate was predetermined at the same wavelength, and the absorbance of the blank well was subtracted from the absorbance of the same well containing the final solution.
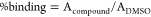
1

#### Secondary Binding Assay

The best ligands from each group that blocked Pin1 WW domain binding of HRP-Pin1 bound to plate were reassayed at 270 μM to identify the best ligands through the same procedures above.

### Competitive Binding *K*_d-obs_ Determination

The competitive dissociation constants, *K*_d-obs_, of cis-**1**{2, *d*}, trans-**1**{2, *d*}, **1**{2,*o*}, and **1**{18, *m*} were determined by ELEBA after the large-scale synthesis, HPLC purification, and quantification. The final concentrations of each compound were: 2.0, 10, 30, 50, 100, 200, 300, 500, 700, 1000, and 2000 μM. Ligands stock concentrations were 88 mM by weight, and the final concentrations for competitive dissociation constants (*K*_d_) were 2000, 1000, 700, 500, 300, 200, 100, 50, 30, 10, and 5.0 μM. Recombinant human Pin1–HRP (R&D Systems) was diluted to a final concentration of 200 pM in a PBS buffer containing 2% BSA at a pH of 7.3. The Pin1–HRP in the ligand solution was then preincubated with ligand for 30 min with orbital shaking. The resulting solutions containing Pin1–HRP and ligand were added to the 96-well plate 100 μL/well and incubated for one additional hour. The solutions were decanted, and the wells were washed with wash buffer containing 10 nM imidazole, and 0.05% Tween 20 in PBS at pH 7.3 (3 × 150 μL/well). The substrates hydrogen peroxide and 3,3′,5,5′-tetramethylbenzidine (R&D Systems) were added to the plate in a 1:1 ratio (100 μL/well) and incubated at room temperature for 20 min in the dark. The stop solution, 2 N H_2_SO_4_ (50 μL/well), was then added to the 96-well plate in order to ensure similar kinetics among the samples. The optical density (OD) of each well was immediately determined using a plate reader (Spectra Max 340 PC) with UV absorbance detection at 450 nm. The concentrations and the corresponding % bound were fit to eq 2, where [L] is the concentration of a ligand, and a, b, and c were fitted constants for each curve.
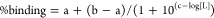
2

### Modeling Methods

The RNA polymerase domain *C*-terminal domain peptide, YpSPTpSPS, bound to the Pin1 WW domain in the crystal structure, 1F8A.pdb, was modified to create a model of each ligand, *cis*- and *trans*-**1**{2,*d*}, **1**{2, *n*}, and **1**{18, *l*} bound in the WW domain (Figure 6). The Pin1 protein was prepared by removing all water molecules with less than one hydrogen bond to nonwaters, adding and minimizing all hydrogens, then minimizing the protein with the OPLSe force field. Then the ligand and protein were minimized by OPLSe using Macromodel software (Schrödinger, Inc.).

### NMR Chemical Shift Perturbations (CSPs) and *K*_d_ Calculations

Full-length Pin1 was overexpressed and purified as described previously.^47^ The final NMR buffer conditions were 30 mM imidazole-D_4_, pH 6.6, with 30 mM NaCl, 0.03% NaN_3_, 5 mM DTT-*d*_10_, and 90%H_2_O/10%D_2_O. The ligand stock solutions included 2.4 and 24 mM for **1**{*18,l*}; 2.4 and 16 mM for *trans*-**1**{*2,d*}; 2.4 and 12 mM for *cis*-**1**{*2,d*}; 2.4 mM and 12 mM for **1**{*2,n*}. The stock concentrations were confirmed by standard 1-D ^1^H NMR of the samples diluted in the buffer described above.

The NMR titration spectra were recorded at a nominal temperature of 295 K, on a Bruker Avance 700 MHz (16.4 T) spectrometer equipped with a cryogenically cooled TCI probe. Sequential resonance assignments for wild type Pin1 were from our prior publications.^34,35,46−48^ Data processing and cross-peak analysis used Topspin 1.3 and 2.1 (Bruker Biospin, Inc.), and Sparky (T. D. Goddard and D. G. Kneller, SPARKY 3, University of California, San Francisco). The initial Pin1 concentration (apo-Pin1) was 48.6 μM. We recorded 2-D ^15^N–^1^H HSQC spectra per Mori et al.^51^ after each addition of ligand to get NH binding isotherms (NH chemical shift perturbations as a function of the total ligand concentration). Each 2-D data set consisted of 128 FIDs (64 complex points), with 16 scans per FID and a recycle time of 1s. The ^1^H^N^ spectral width was 15 ppm, centered at 4.703 ppm; the ^15^N spectral width was 35.24 ppm, centered at 118 ppm. The binding-induced ^15^N–^1^H chemical shift perturbations (CSPs) were calculated by eq 3.
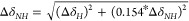
3^35^

The Δδ_H_ and Δδ_N_ terms are the amide ^1^H and ^15^N chemical shift differences relative to isolated Pin1. To estimate the ligand *K*_d_ values for WW domain binding, we globally fitted WW domain NH binding isotherms to the expression for the bound protein fraction in a standard two-state binding equilibrium, P + L ⇌ PL, as described in our previous Pin1 studies.^35^ The monitored WW domain residues included K13, R14, M15, S16, G20, R21, A31, S32, Q33, W34, and E35. The estimated statistical uncertainties in *K*_d_ are the changes yielding a 1% increase in the chi-squared error.

## Supporting Information Available

Primary assay results; ^1^H, ^13^C, ^31^P NMR spectra for synthetic intermediates and products **1**{*18,l*}, **1**{*2,n*}, *cis*-**1**{*2,d*}, *trans*-**1**{*2,d*}; and HSQC spectra for Pin1 bound to **1**{*18,l*}, **1**{*2,n*}, *cis*-**1**{*2,d*}, *trans*-**1**{*2,d*} (PDF)
Raw ELEBA data (XLSX)
