# Supplementary material for: Pin1 WW Domain Ligand Library Synthesized with an Easy Solid-Phase Phosphorylating Reagent
Source: Biochemistry. 2024 Oct 8;63(21):2803–15. doi: 10.1021/acs.biochem.4c00231 (PMC11542186; doi:10.1021/acs.biochem.4c00231)
Supplement: Supplementary file 1 — bi4c00231_si_001.pdf [file bi4c00231_si_001.pdf]

Supporting Information

Pin1 WW Domain Ligand Library Synthesized with an Easy Solid-Phase Phosphorylating Reagent

Xingguo R. Chen,[†1] Ana Y. Mercedes-Camacho,[†1] Kimberly A. Wilson,[2] Jill J. Bouchard,[2] Jeffrey W. Peng,[2*] Felicia A. Etzkorn[1*]
[1]Department of Chemistry, Virginia Tech, Blacksburg, VA 24061, [2]Department of Chemistry and Biochemistry, University of Notre Dame, Notre Dame, IN 46556
[†]Co-first authors.
*Email: fetzkorn@vt.edu, jpeng@nd.edu

Synthetic method development. A solution of TFA:CH_2Cl_2 1:2 was used to cleave the phosphoramidite group from the resin SPPR-**2**, and ^{1}H NMR of the resulting residue showed the presence of the *N*,*N*-diisopropyl and 2-cyanoethyl group (data not shown). Fmoc–Ser–OH was used as a model substrate to test our SPPR. In the presence of 5-ethylthio-1*H*-tetrazole, the substrate was phosphitylated with SPPR **2**, followed by oxidation with *t*-butyl hydroperoxide to give the phosphorylated Fmoc-pSer-OH attached to the resin. The Fmoc-Ser(2-cyanoethylphosphate)-OH product was cleaved from the resin with TFA. ^{1}H NMR indicated the presence of the Fmoc group, implying that the substrate was successfully attached to the resin (data not shown). To develop the coupling methods, 2-(2-naphthyl)ethylamine and propionic acid were reacted with the pSer–Pro resin. The resulting propanoyl-pSer–Pro–(2-(2-naphthyl)ethyl)-amide (^{1}H NMR below) was excluded from the library assays.

Figure S1 (following pages). Ligands (group A – O) were screening using ELEBA at ca. 266 µM. Fmoc-VPRpTPVGGGK-NH_2 **L2** was used as positive control at 76-87 µM from stock solutions of 4.3-4.8 mM. NSB for some plates was measured in the absence of plate-linked peptide **2**. DMSO was used as negative control. % of Pin1-HRP bound to plate-linked ligand L2 was normalized relative to DMSO as 100% binding. Mean of N = 2 was used for data analysis with standard deviations shown as error bars. (Prepared with Kaleidagraph 4.3.).

pSer-Pro ligand (group A)

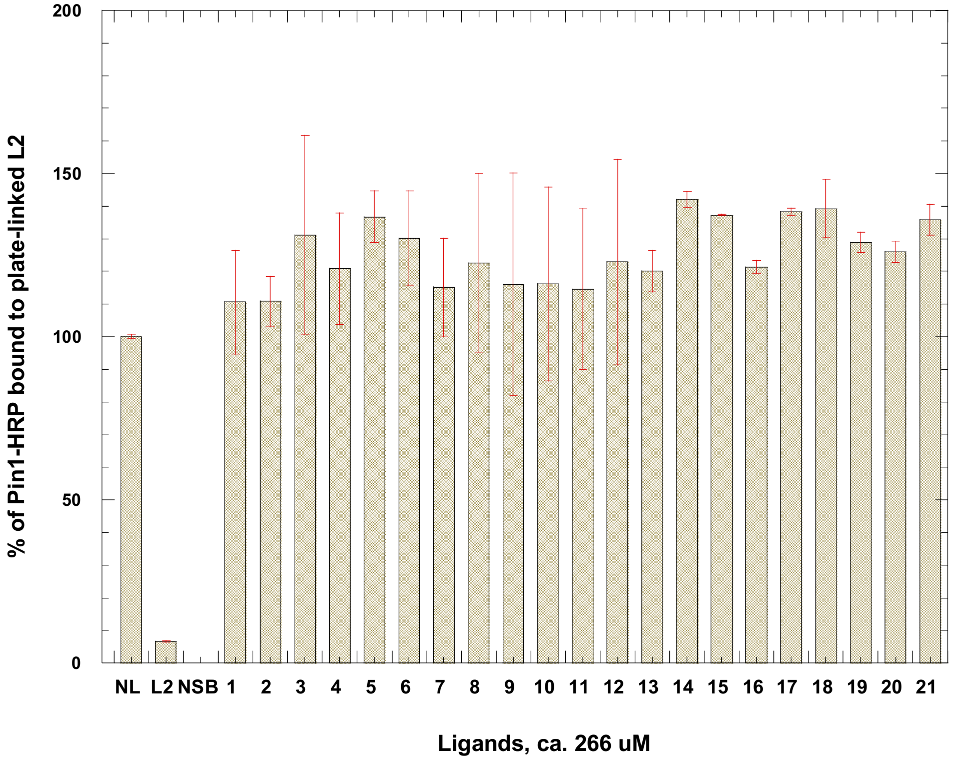

pSer-Pro ligand (group B)

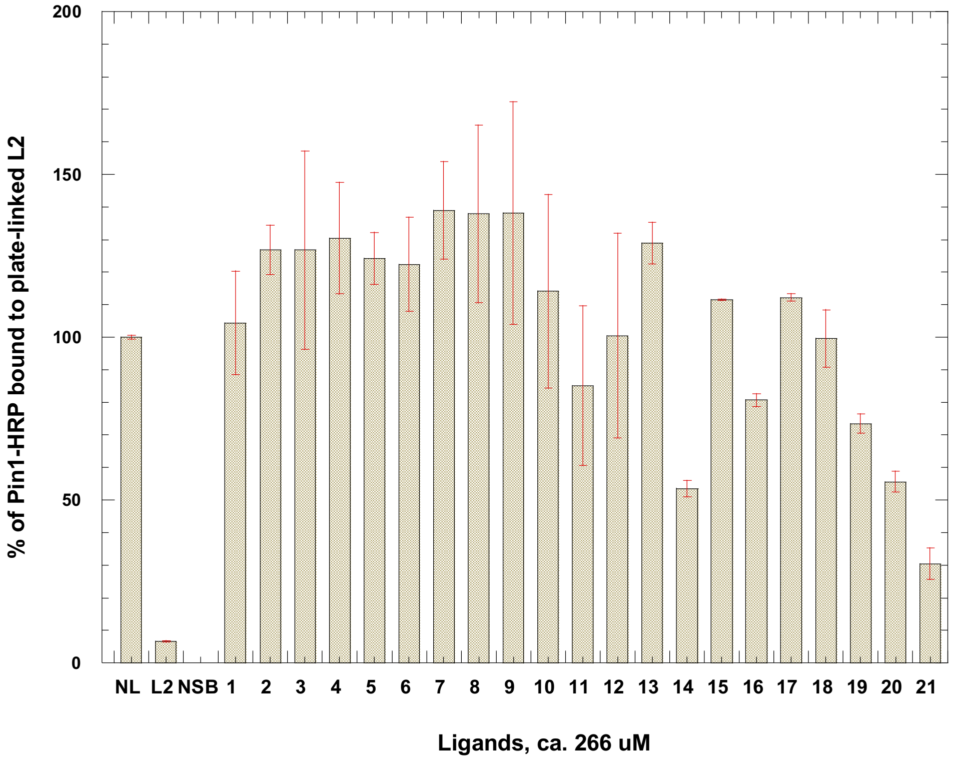

pSer-Pro ligand (group C)

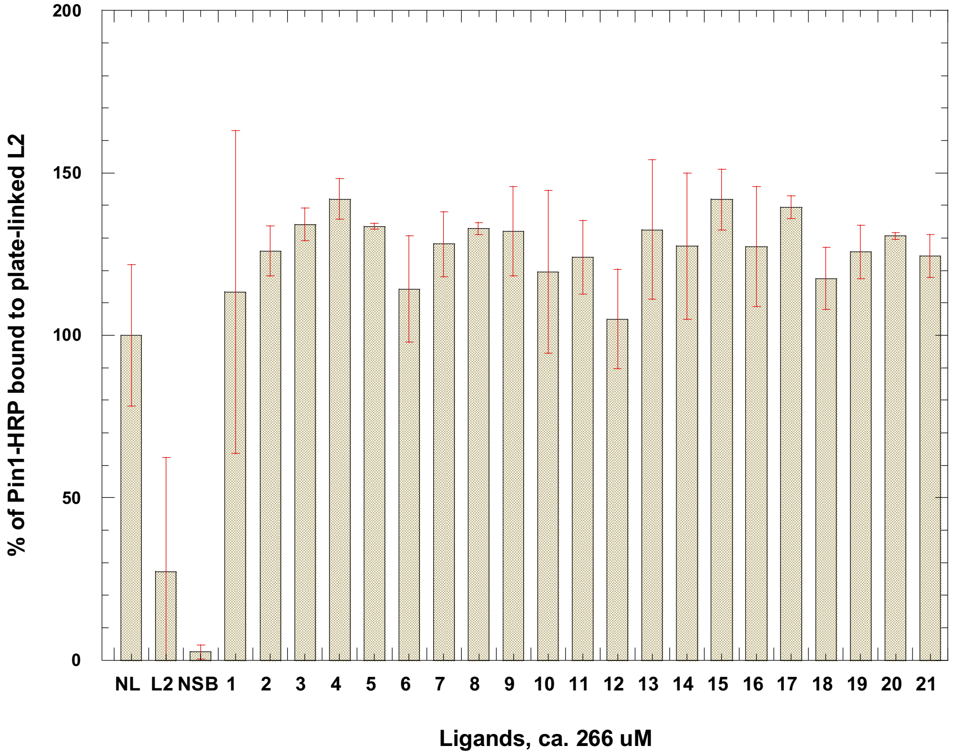

pSer-Pro ligand (group D)

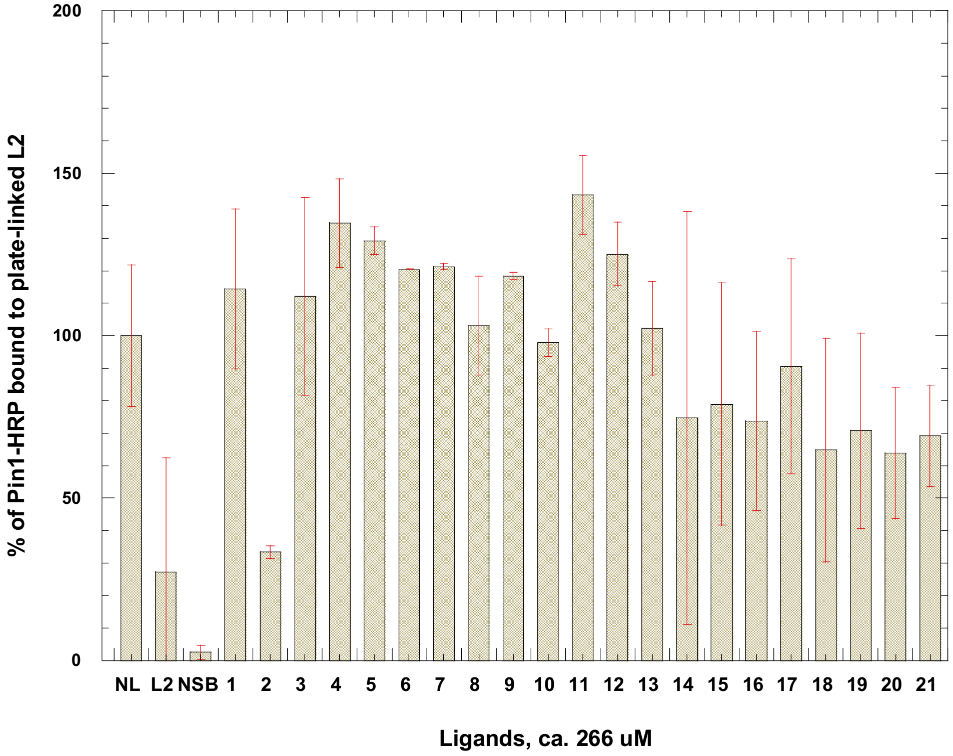

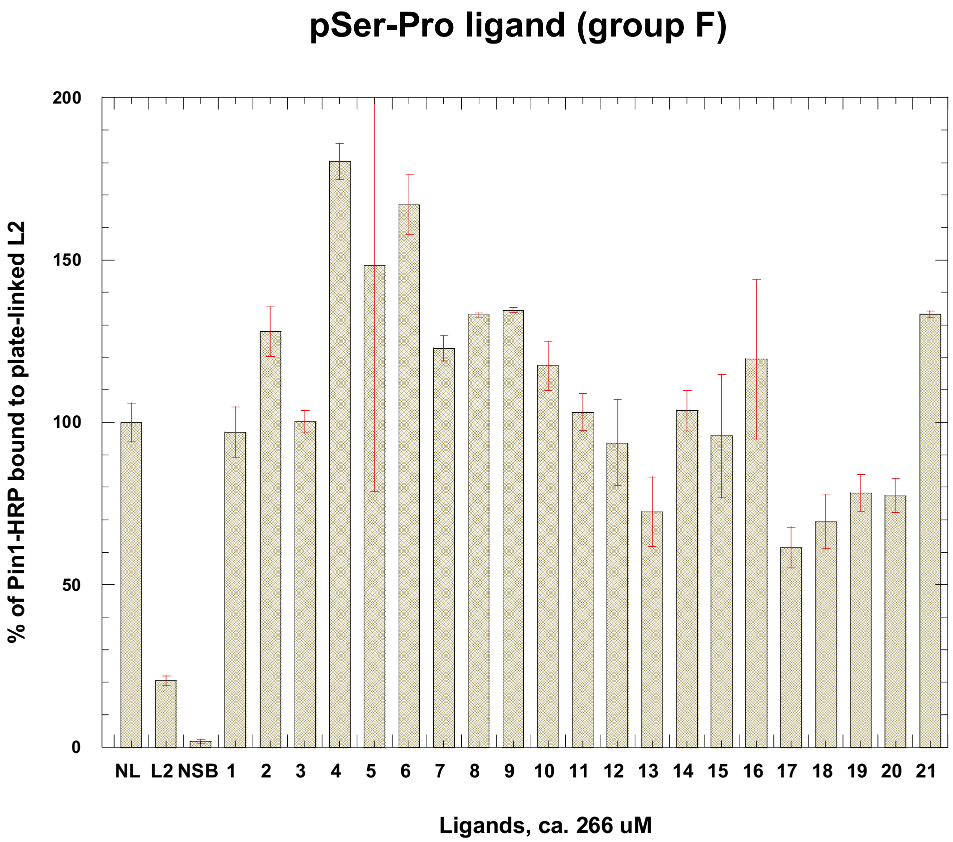
pSer-Pro ligand (group F)
200
150
100
50
0
% of Pin1-HRP bound to plate-linked L2
NL L2 NSB 1 2 3 4 5 6 7 8 9 10 11 12 13 14 15 16 17 18 19 20 21
Ligands, ca. 266 uM

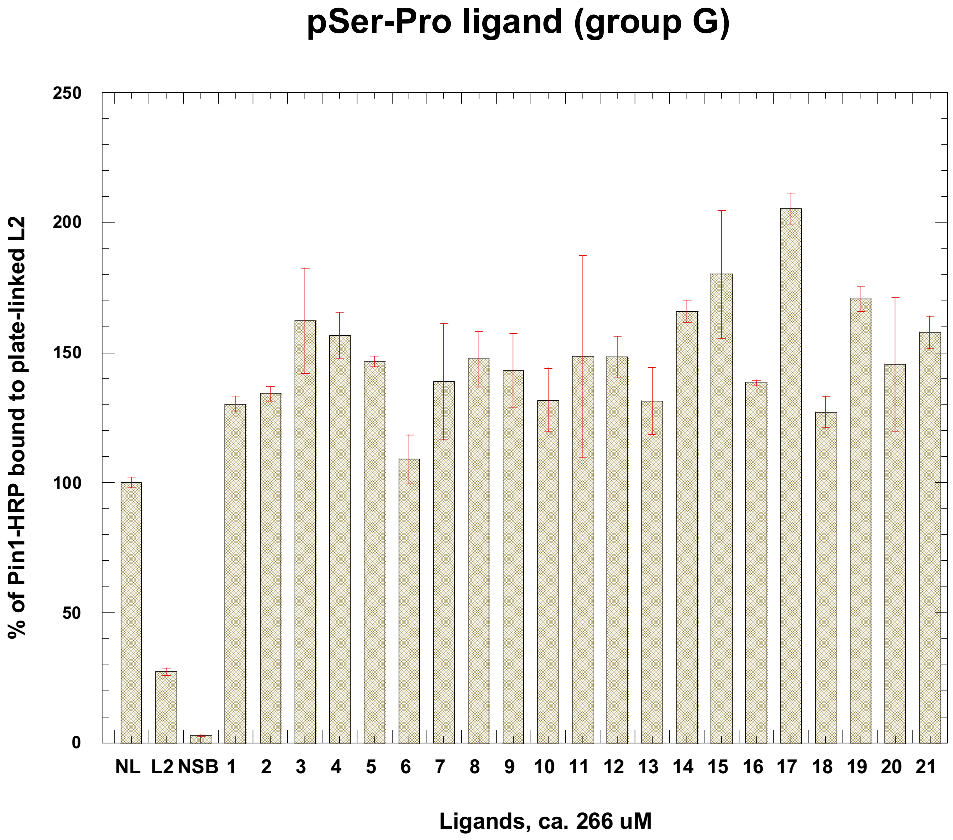
pSer-Pro ligand (group G)
250
200
150
100
50
0
% of Pin1-HRP bound to plate-linked L2
NL L2 NSB 1 2 3 4 5 6 7 8 9 10 11 12 13 14 15 16 17 18 19 20 21
Ligands, ca. 266 uM

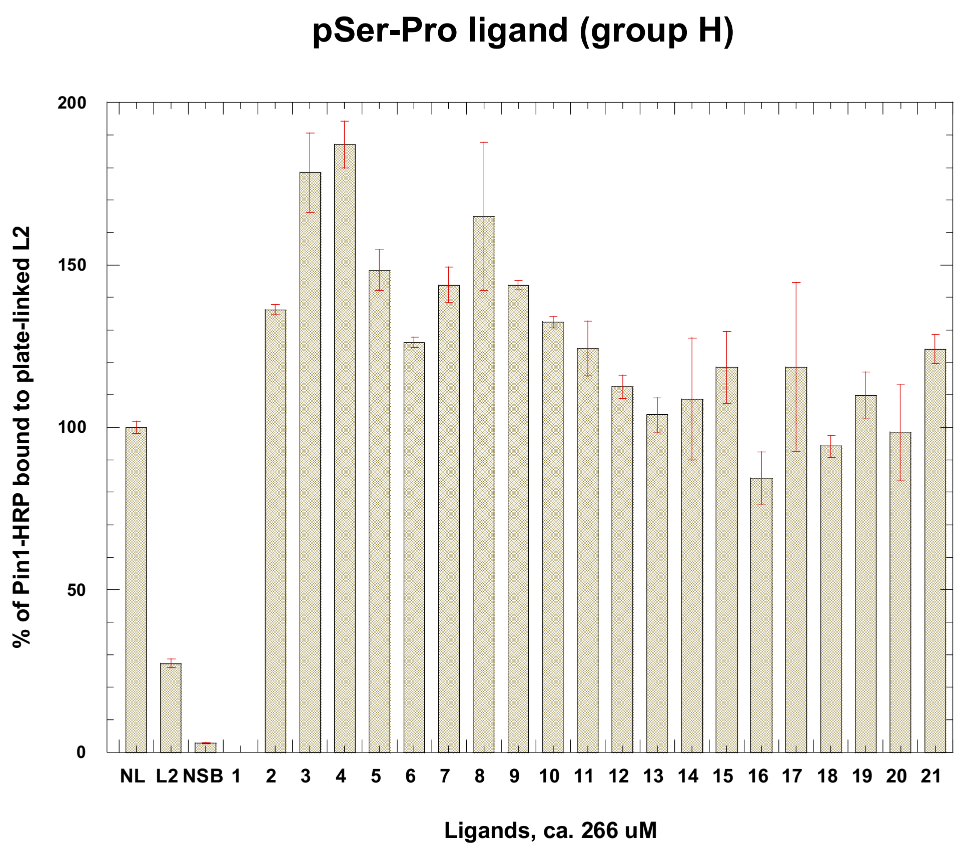
pSer-Pro ligand (group H)
% of Pin1-HRP bound to plate-linked L2
0
50
100
150
200
NL L2 NSB 1 2 3 4 5 6 7 8 9 10 11 12 13 14 15 16 17 18 19 20 21
Ligands, ca. 266 uM

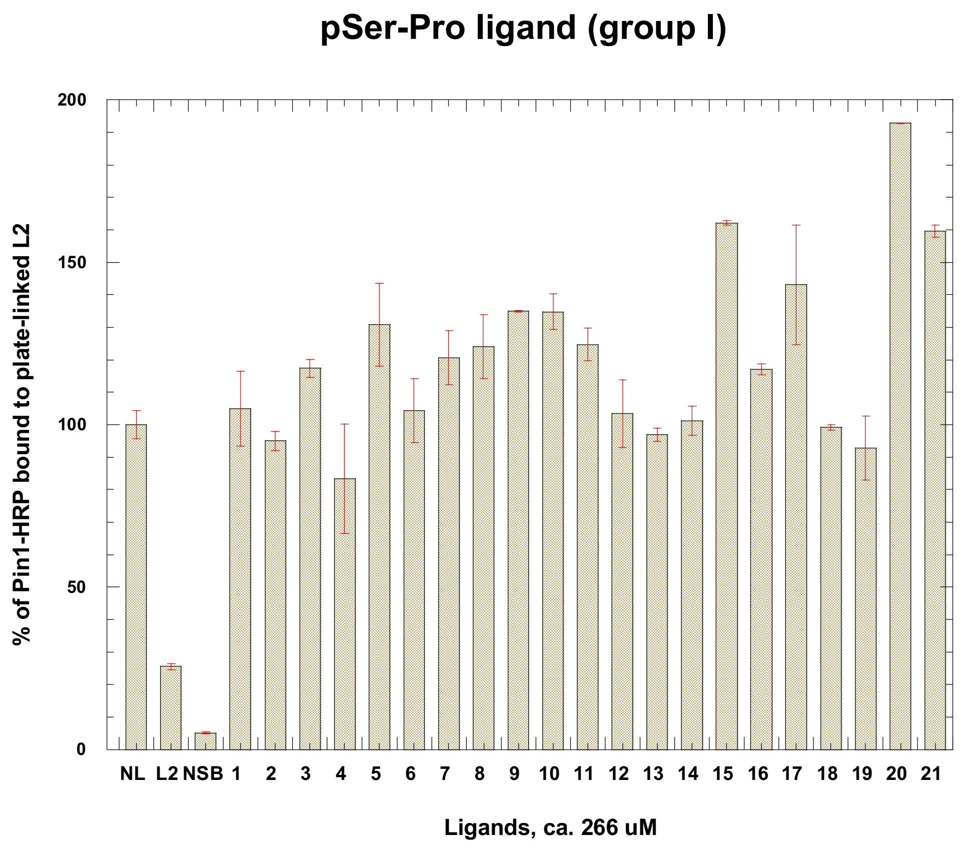
pSer-Pro ligand (group I)
% of Pin1-HRP bound to plate-linked L2
0
50
100
150
200
NL L2 NSB 1 2 3 4 5 6 7 8 9 10 11 12 13 14 15 16 17 18 19 20 21
Ligands, ca. 266 uM

pSer-Pro ligand (group J)

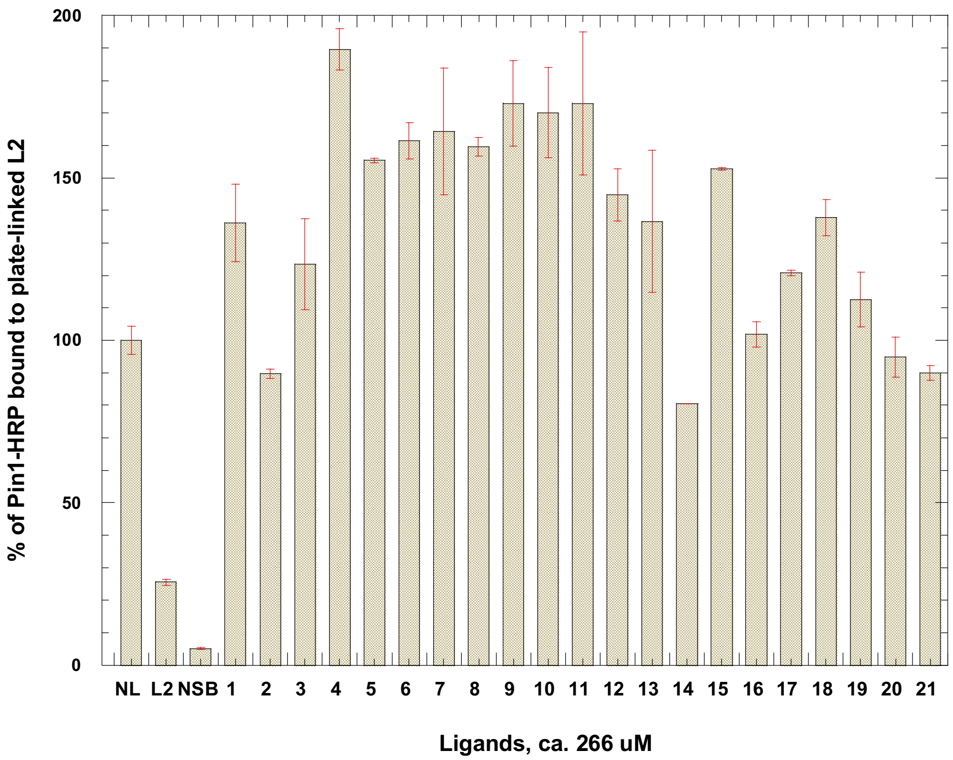

pSer-Pro ligand (group K)

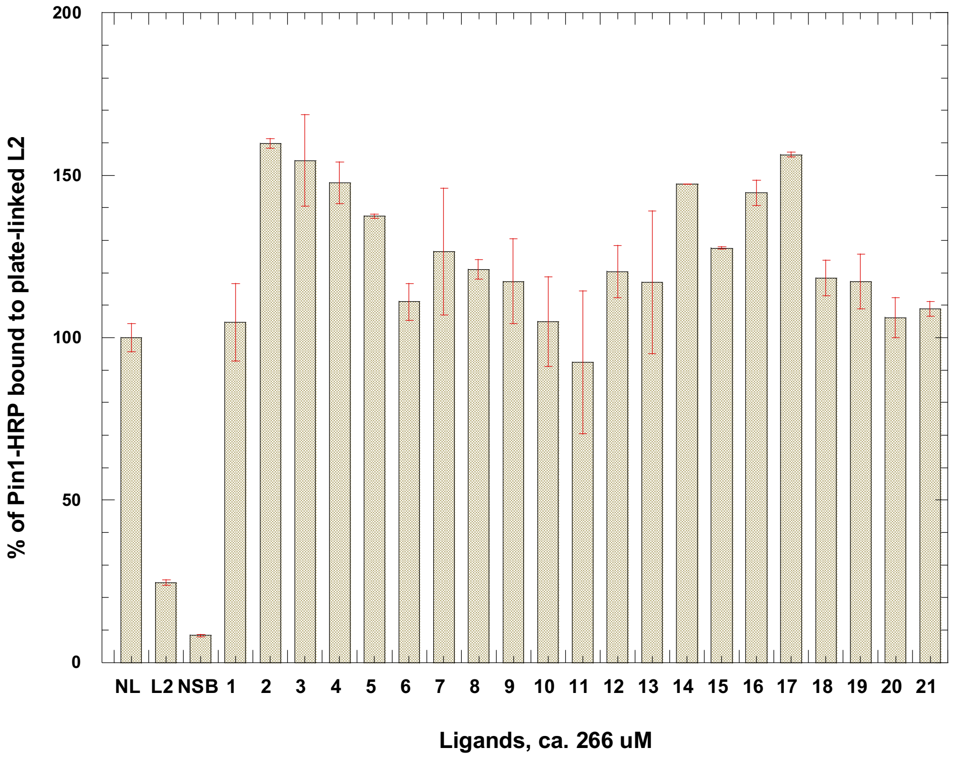

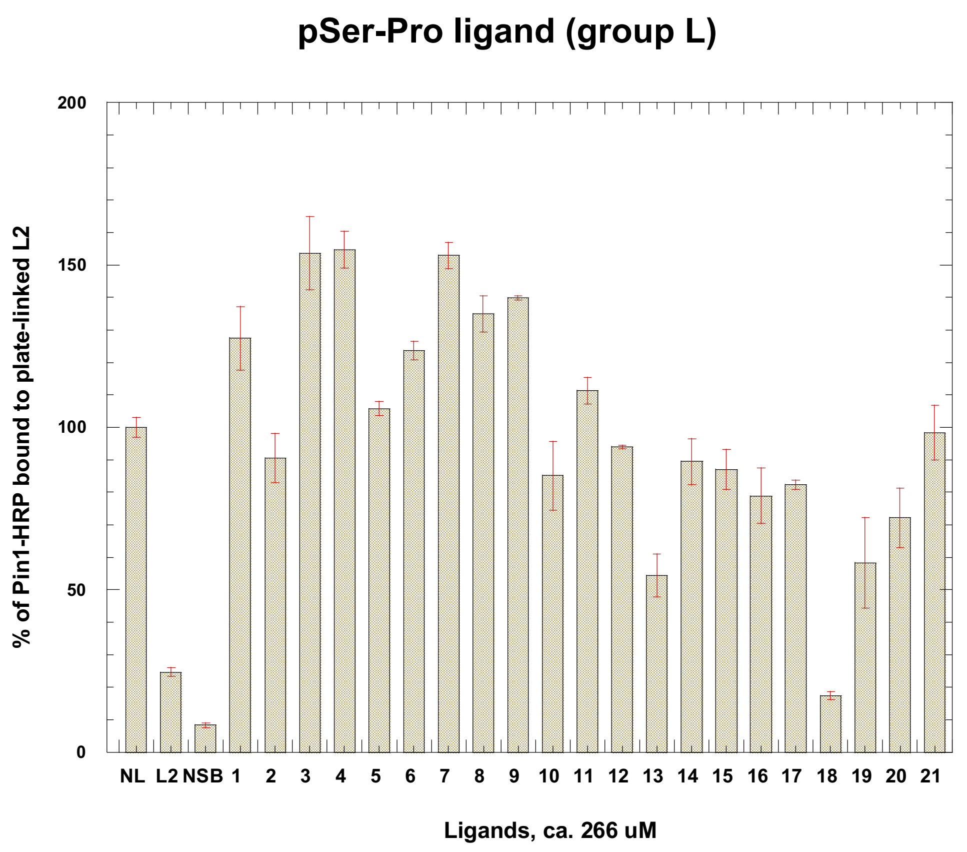
pSer-Pro ligand (group L)
200
150
100
50
0
% of Pin1-HRP bound to plate-linked L2
NL L2 NSB 1 2 3 4 5 6 7 8 9 10 11 12 13 14 15 16 17 18 19 20 21
Ligands, ca. 266 uM

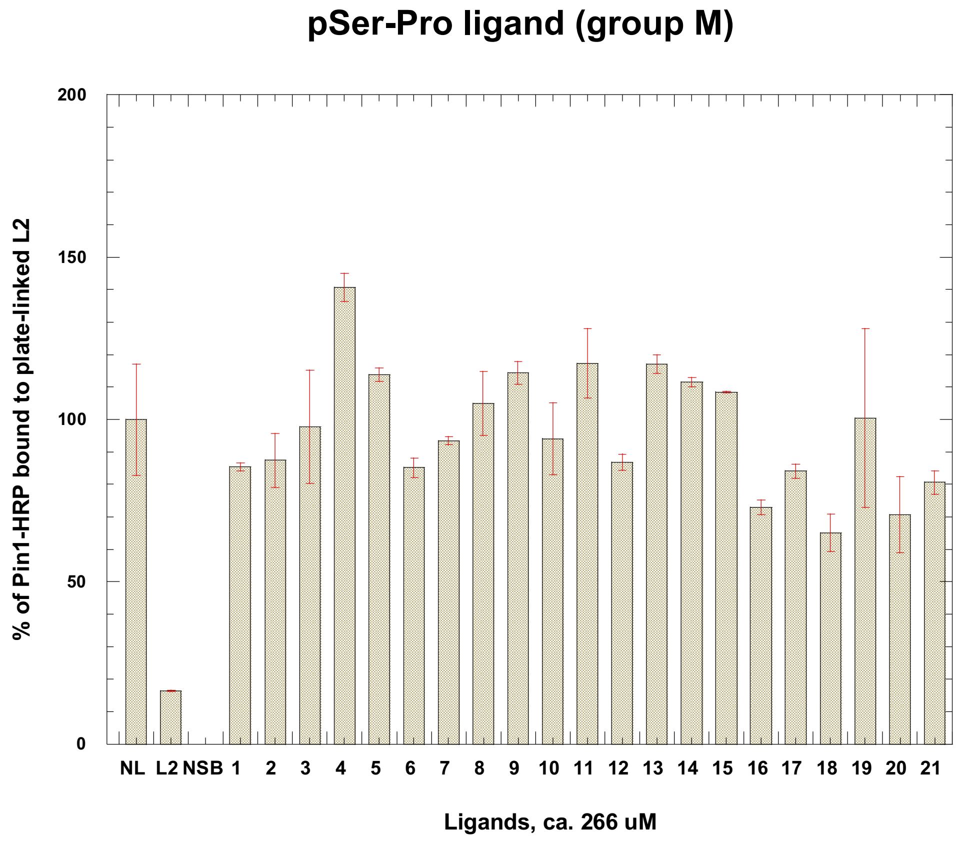
pSer-Pro ligand (group M)
200
150
100
50
0
% of Pin1-HRP bound to plate-linked L2
NL L2 NSB 1 2 3 4 5 6 7 8 9 10 11 12 13 14 15 16 17 18 19 20 21
Ligands, ca. 266 uM

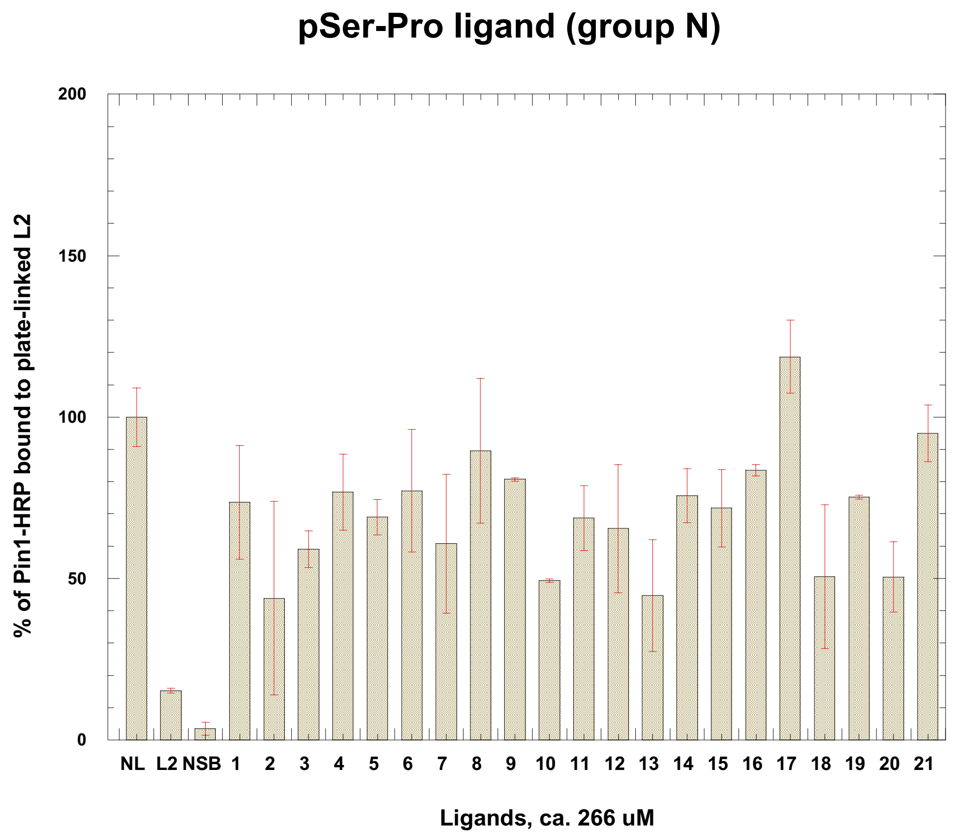
pSer-Pro ligand (group N)
% of Pin1-HRP bound to plate-linked L2
0
50
100
150
200
NL L2 NSB 1 2 3 4 5 6 7 8 9 10 11 12 13 14 15 16 17 18 19 20 21
Ligands, ca. 266 uM

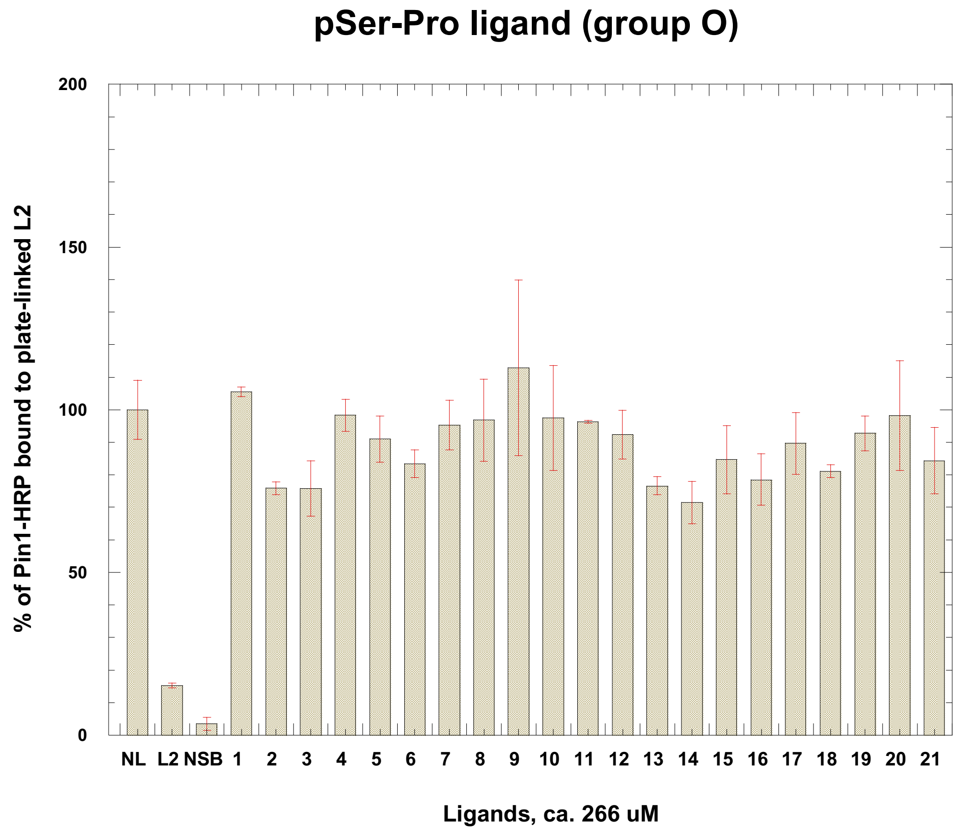
pSer-Pro ligand (group O)
% of Pin1-HRP bound to plate-linked L2
0
50
100
150
200
NL L2 NSB 1 2 3 4 5 6 7 8 9 10 11 12 13 14 15 16 17 18 19 20 21
Ligands, ca. 266 uM

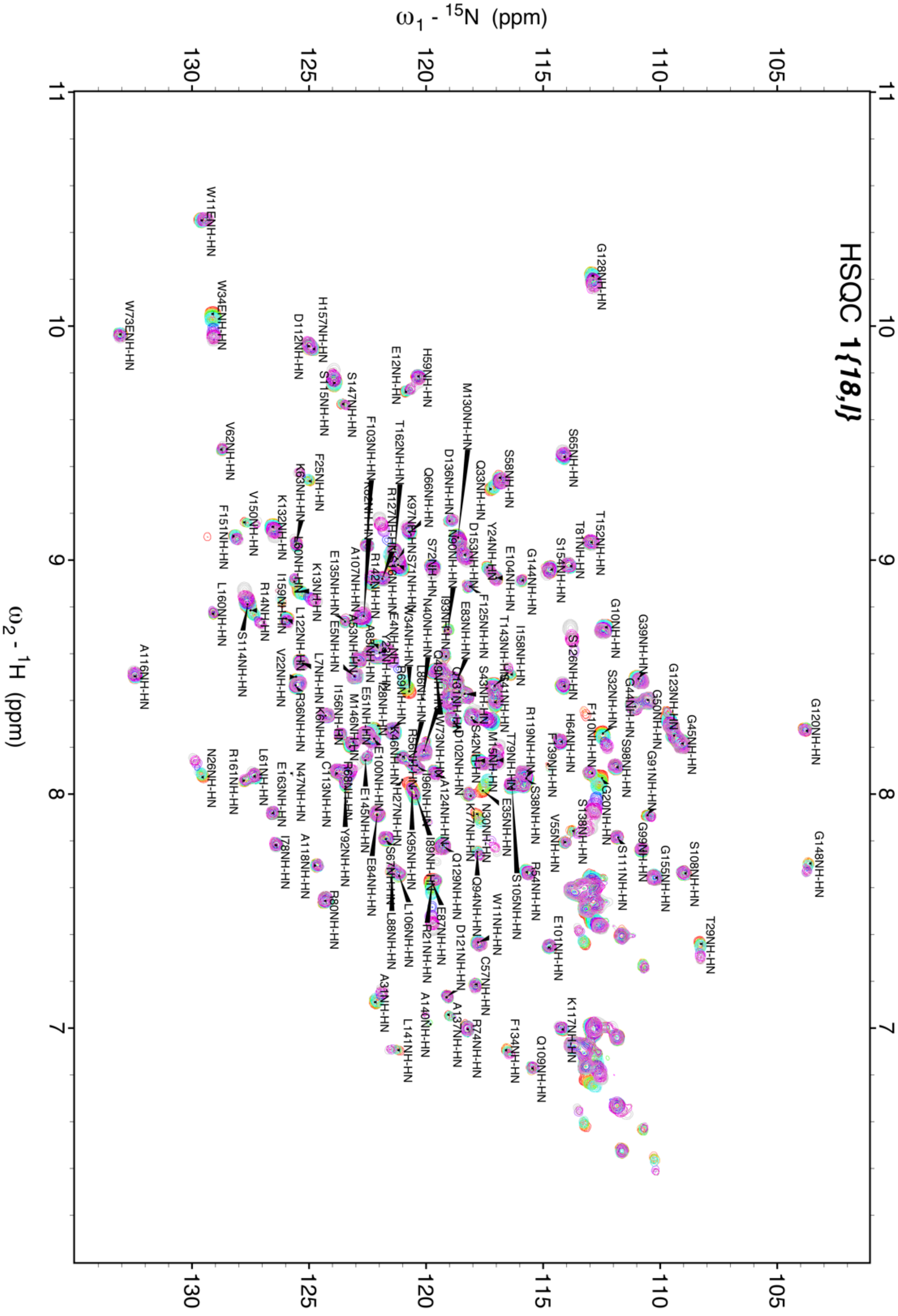

Figure S2. ^{15}N,^{1}H HSQC (700 MHz) overlayed titration spectra for **1{*18,l*}**.

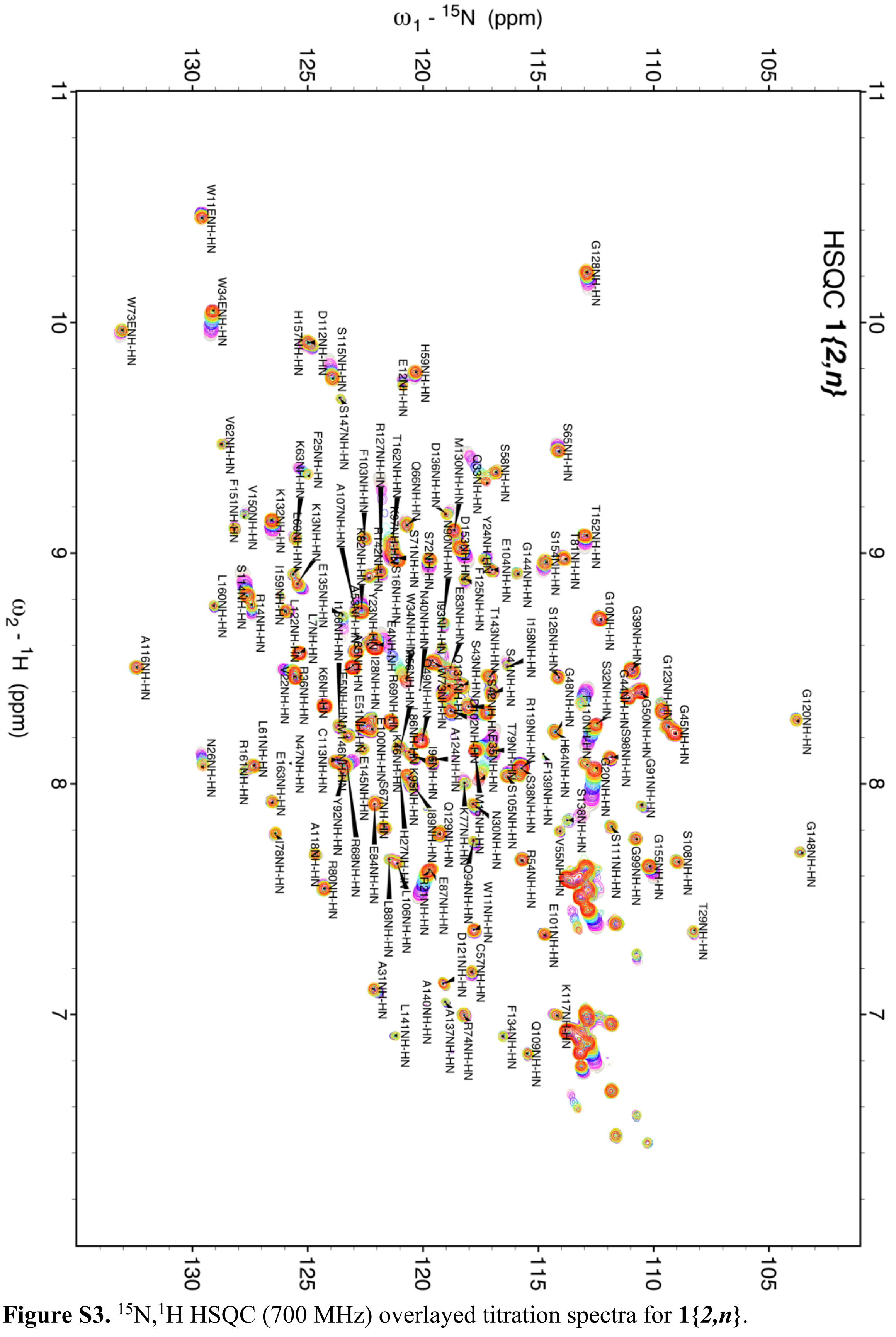

Figure S3. ^{15}N,^{1}H HSQC (700 MHz) overlayed titration spectra for **1{*2,n*}**.

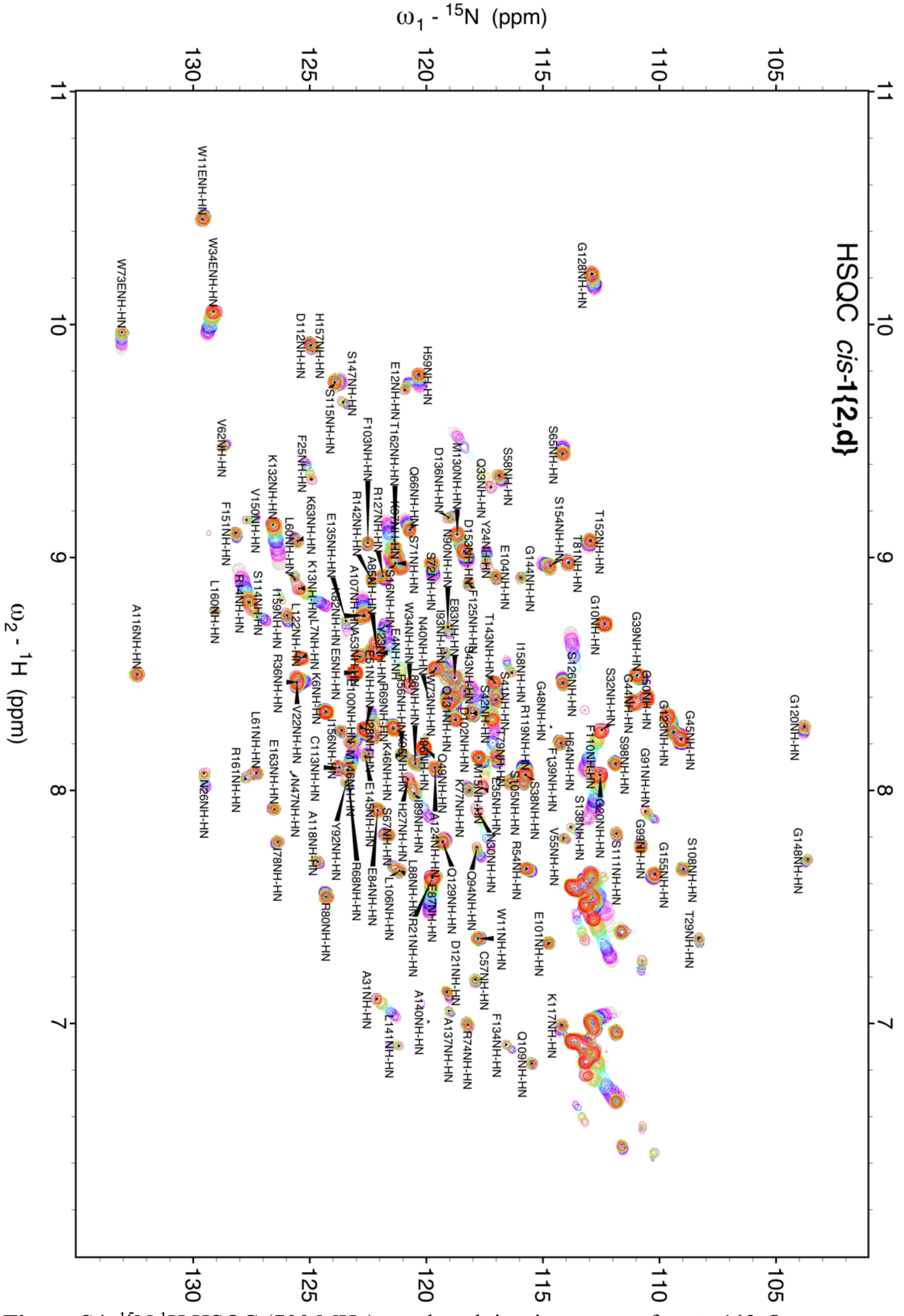

Figure S4. $^{15}N,^{1}H$ HSQC (700 MHz) overlayed titration spectra for *cis*-**1{*2,d*}**.

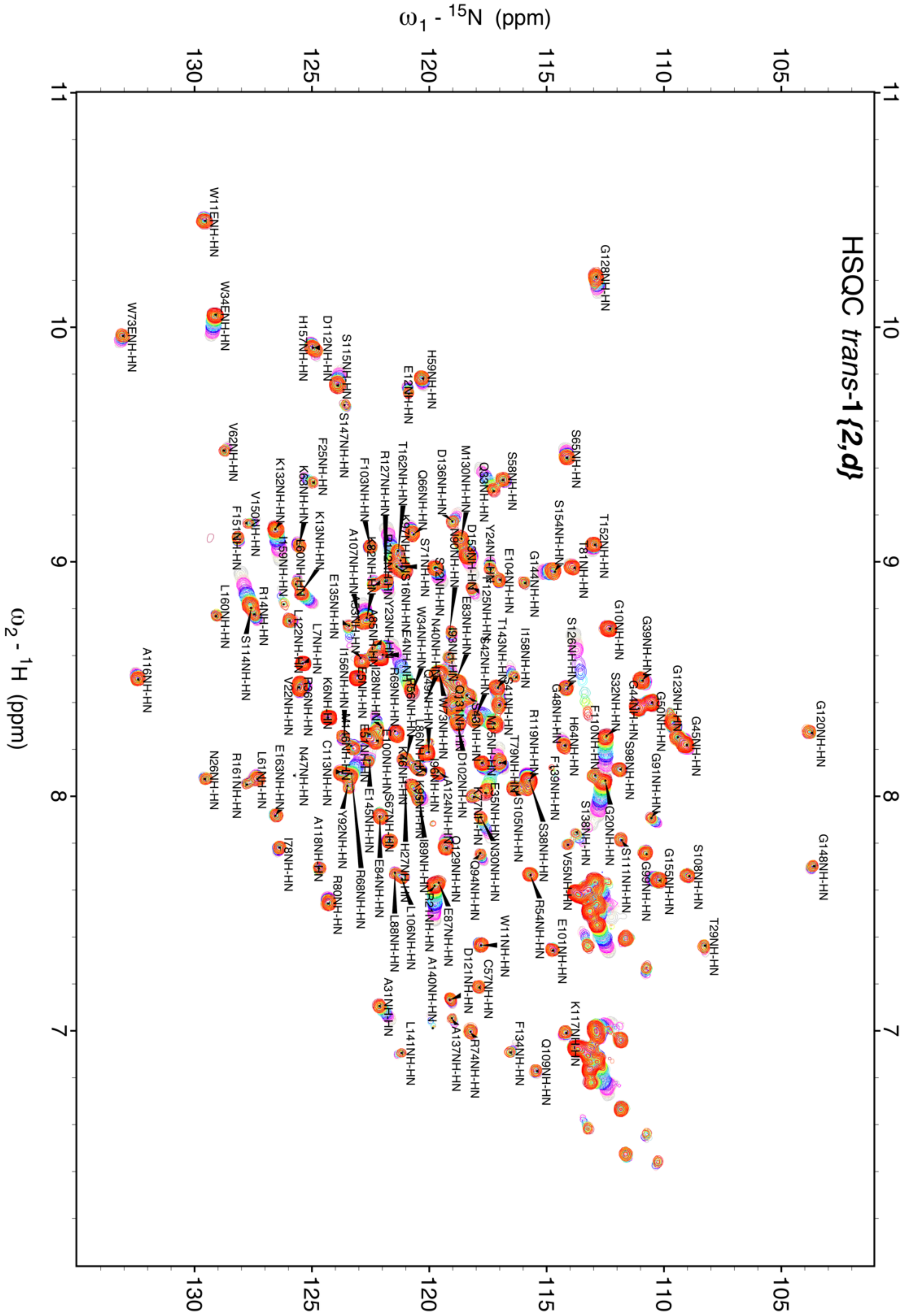

Figure S5. ^{15}N,^{1}H HSQC (700 MHz) overlayed titration spectra for *trans*-**1{*2,d*}**.

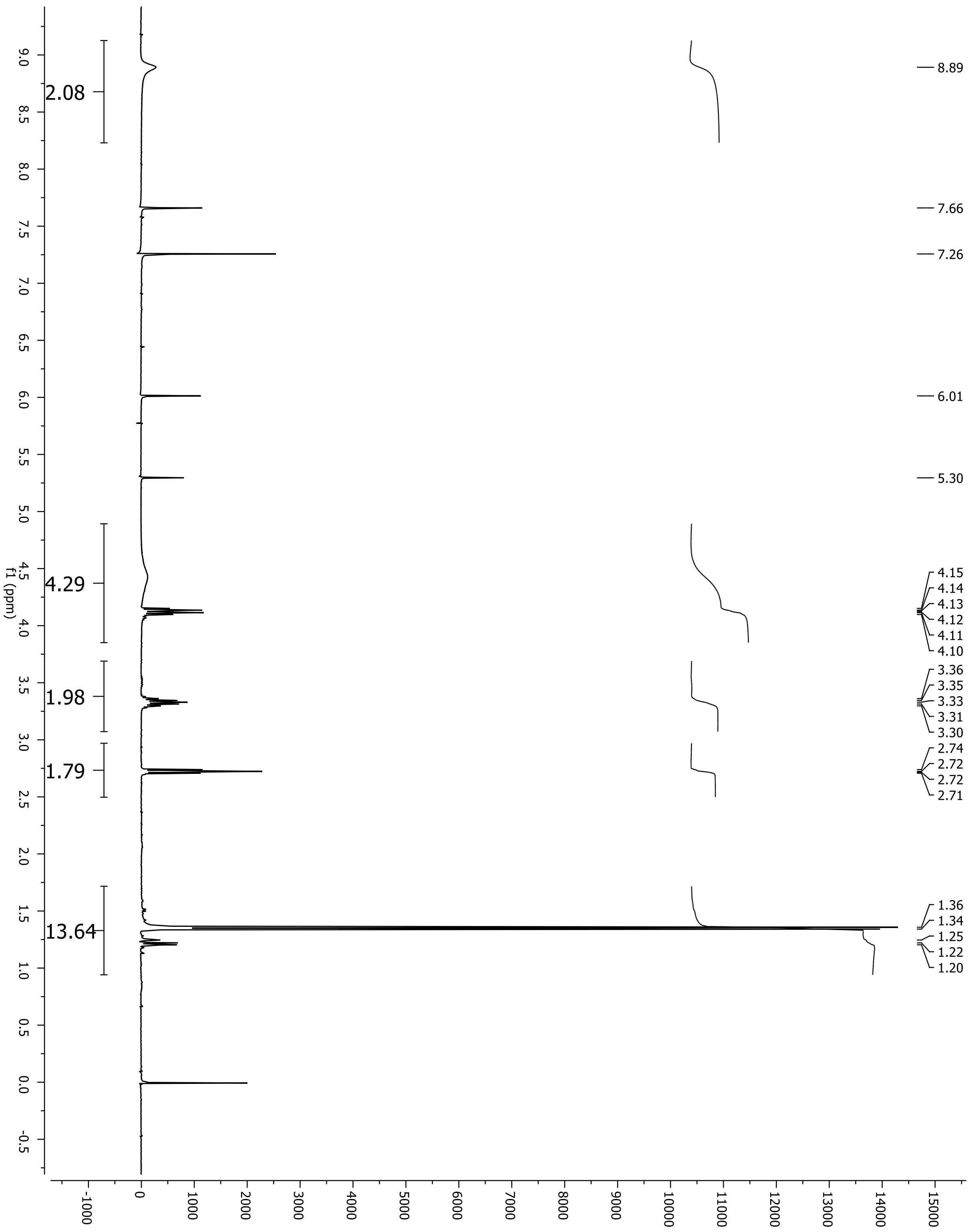

^{1}H NMR of the mixture of 2-cyanoethyl phosphite and *i*Pr_2NH · TFA salt cleaved from SPPR-**2** in $CDCl_3$ (400 MHz)

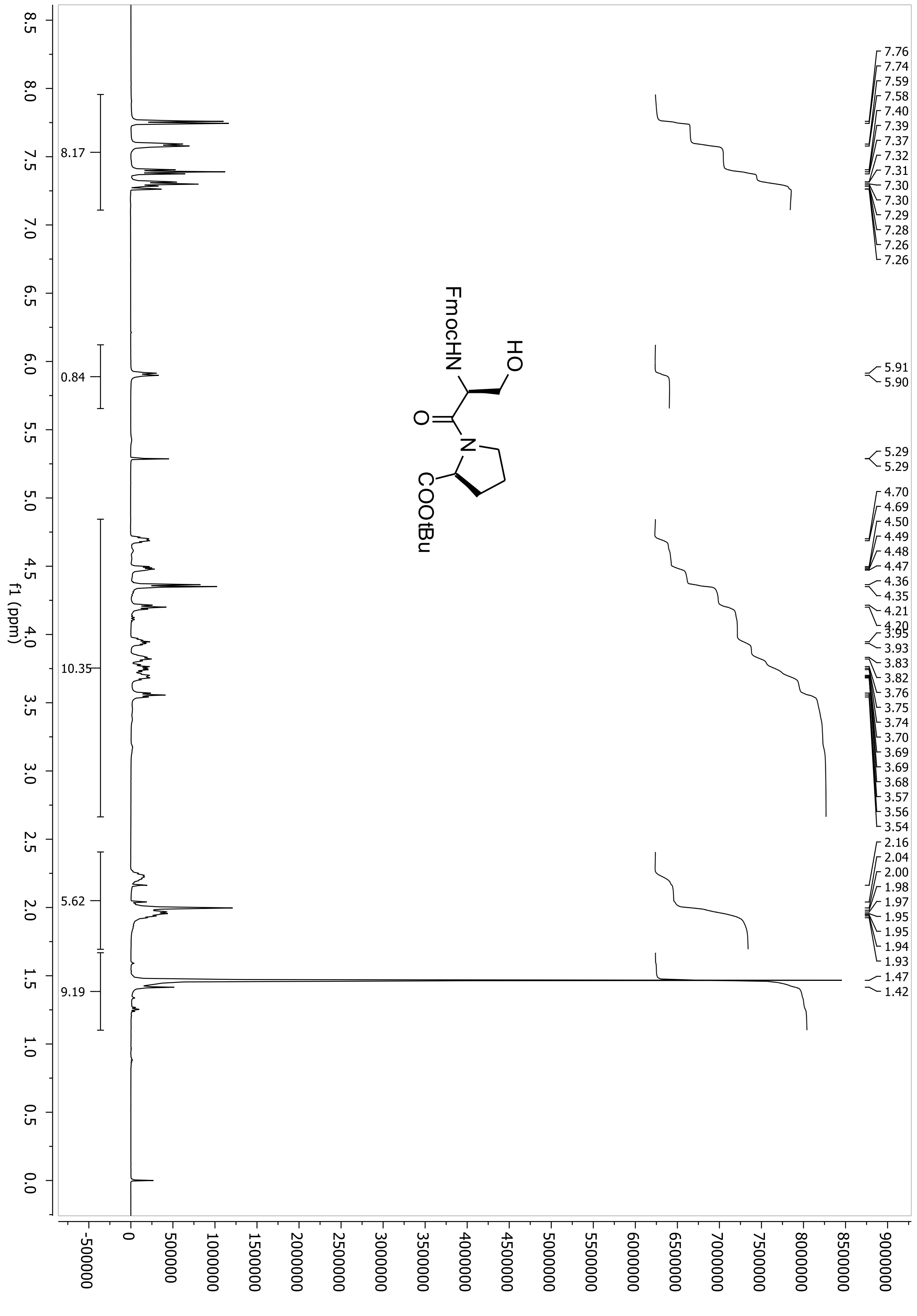

^{1}H NMR of **4** in $CDCl_3$ (500 MHz)

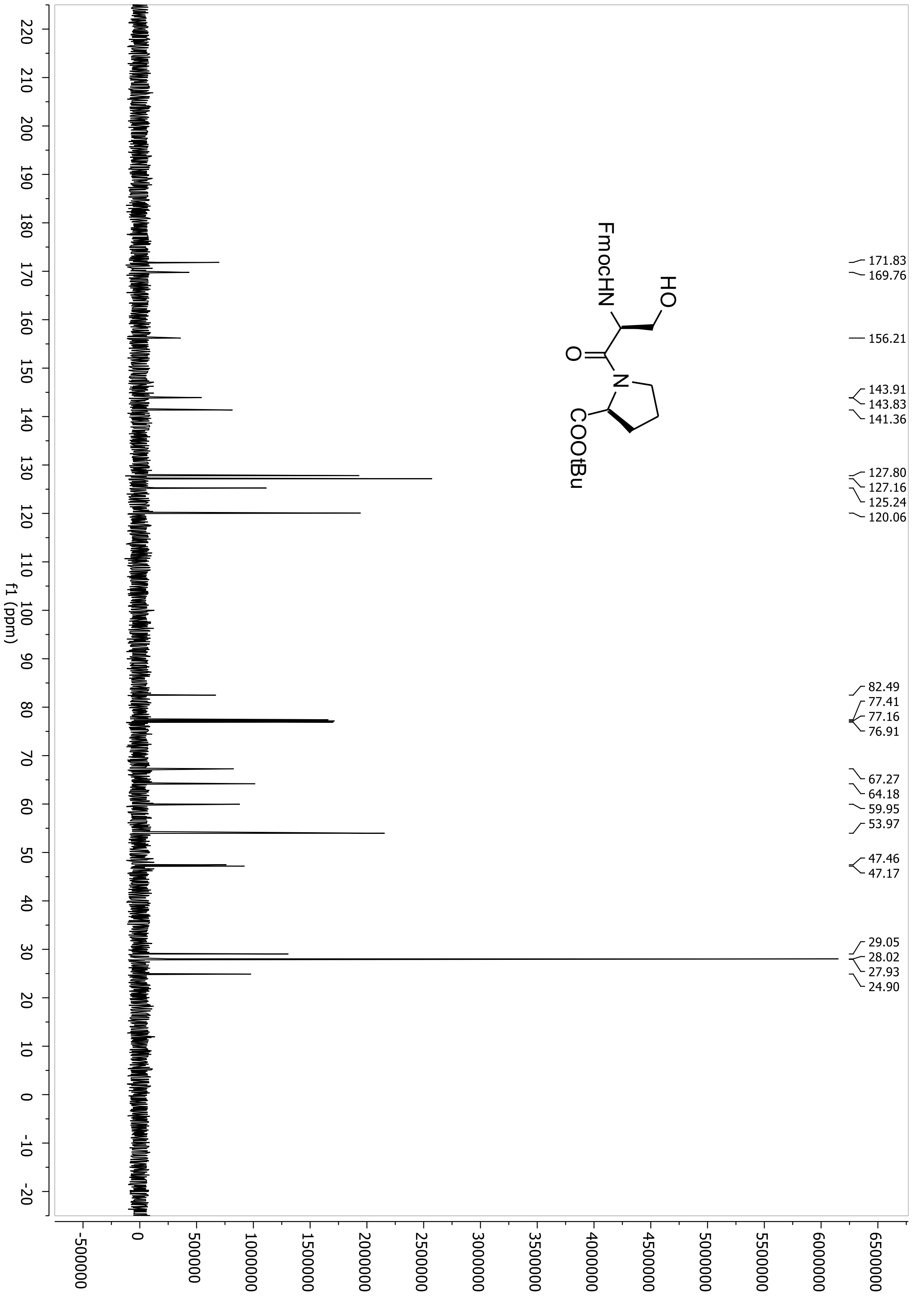

^{13}C NMR of **4** in $CDCl_3$ (125 MHz)

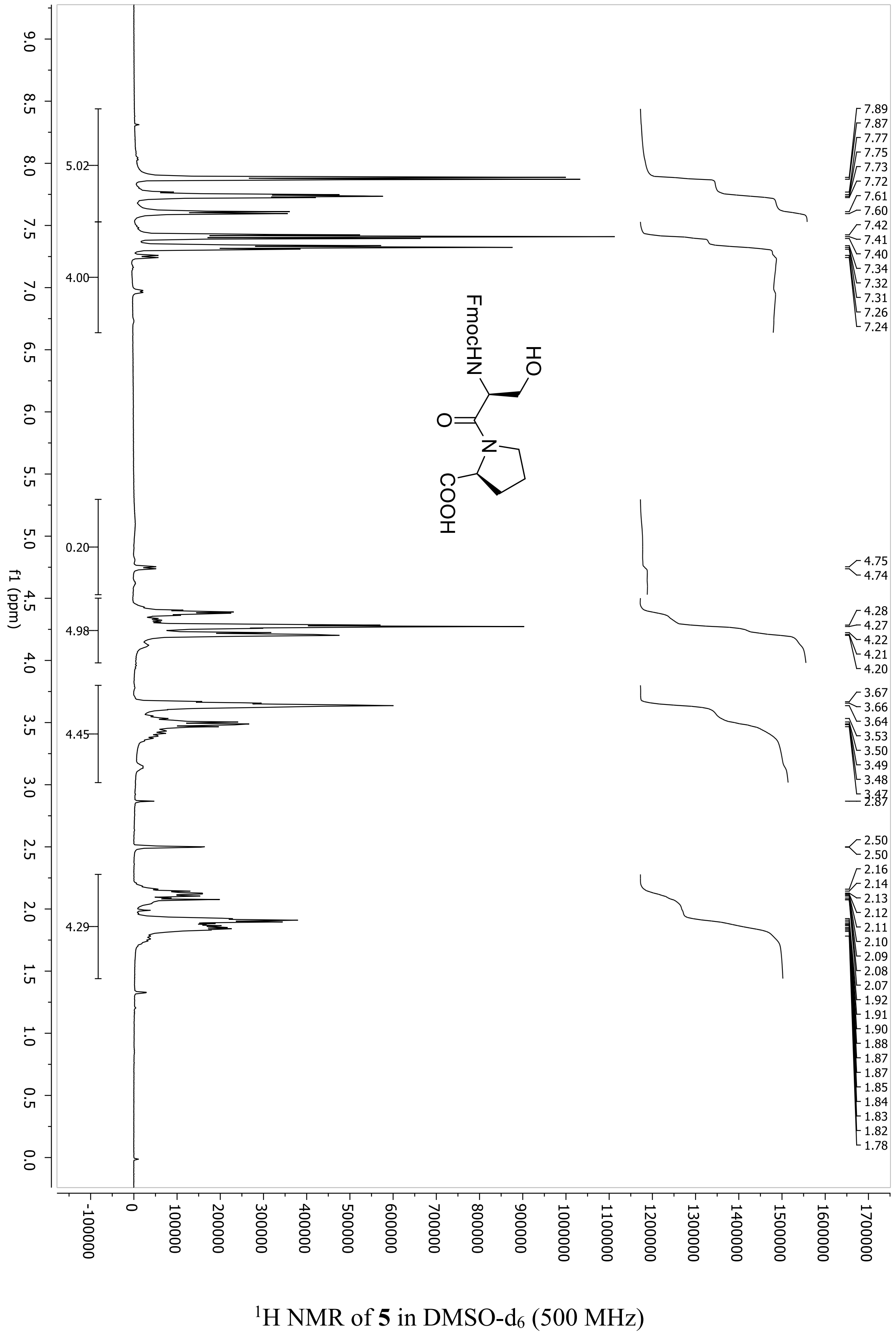

^{1}H NMR of **5** in DMSO-d$_6$ (500 MHz)

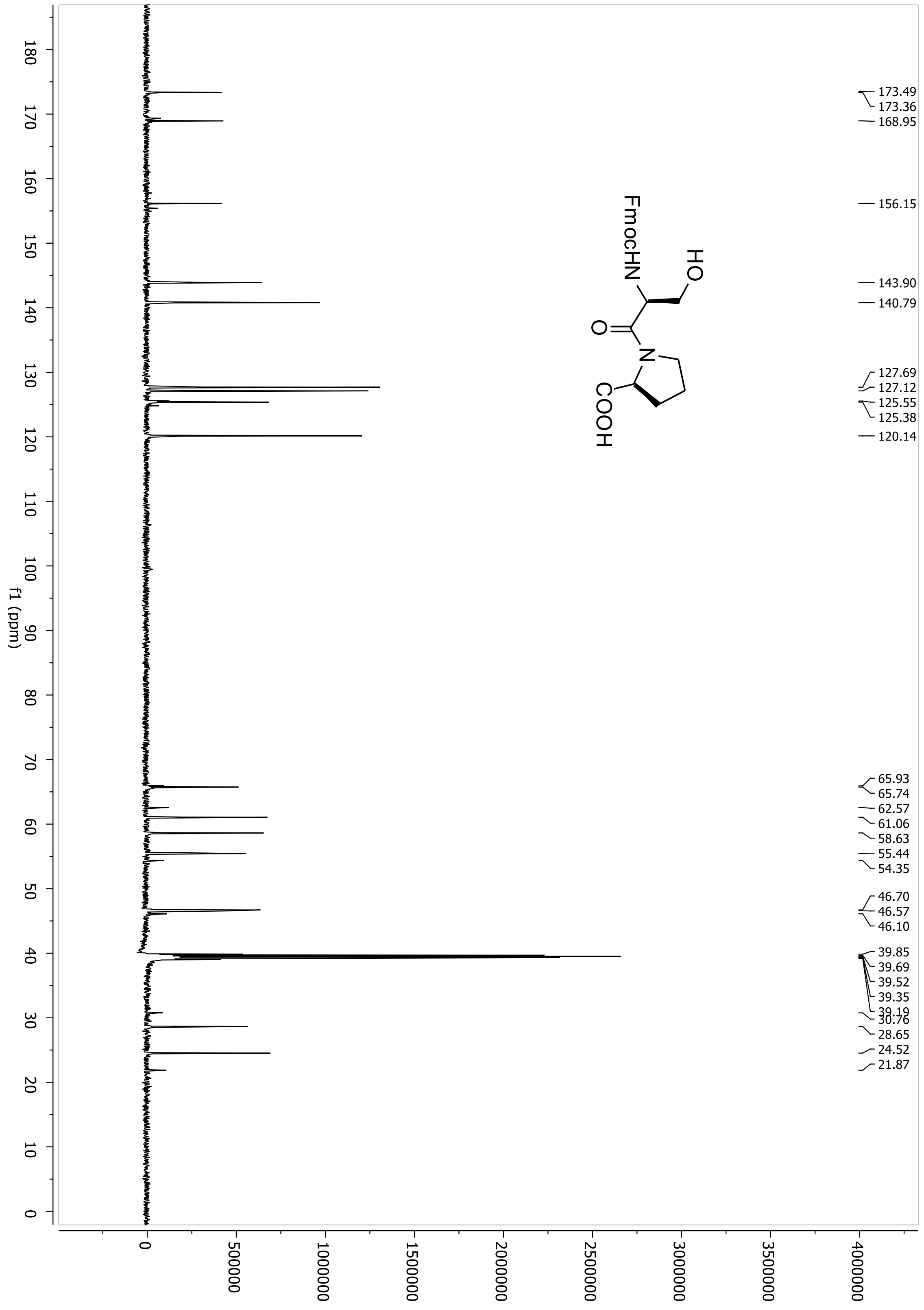

^{13}C NMR of **5** in DMSO-d$_6$ (125 MHz)

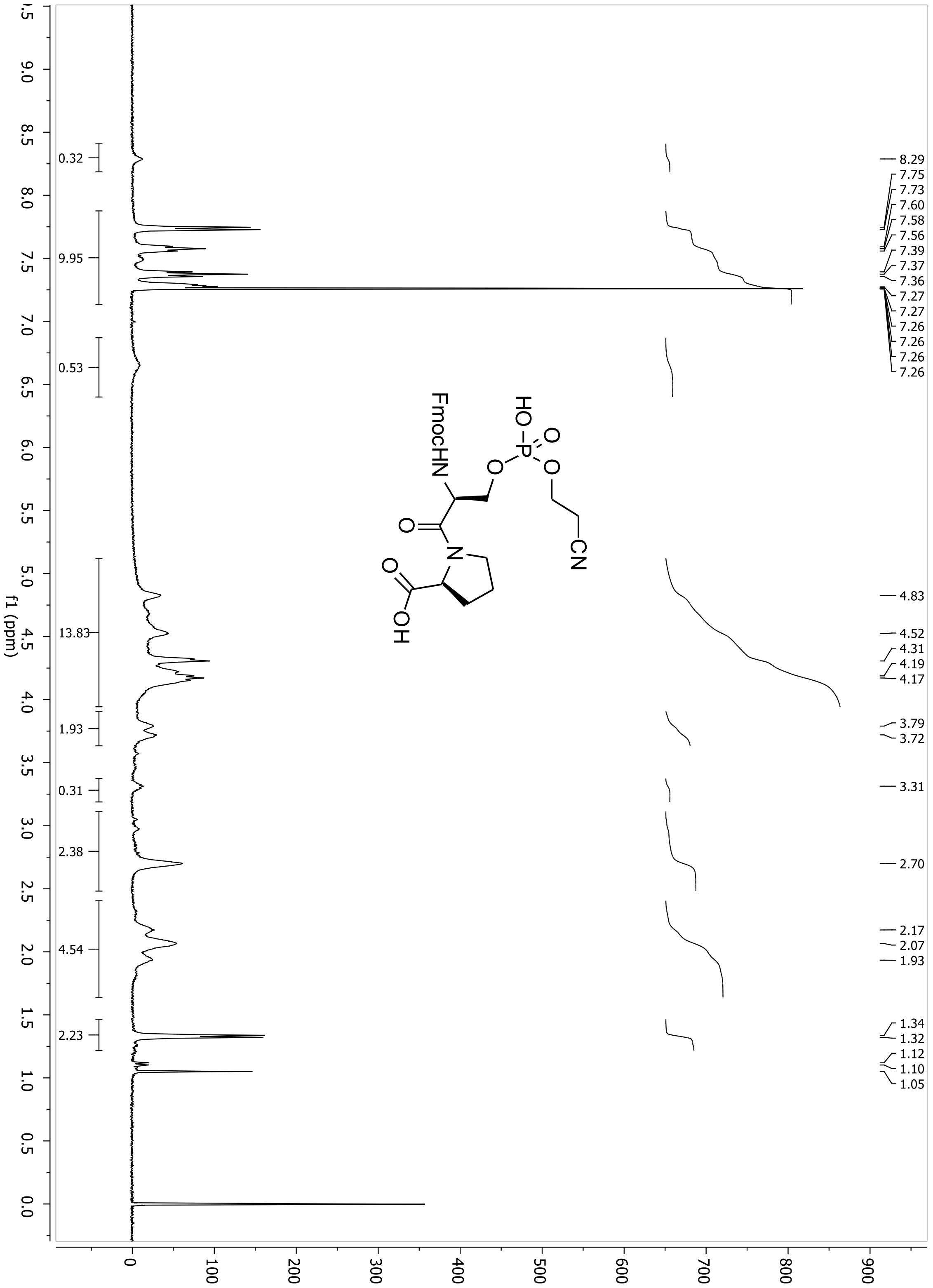

^{1}H NMR of **6** in $CDCl_3$ (500 MHz)

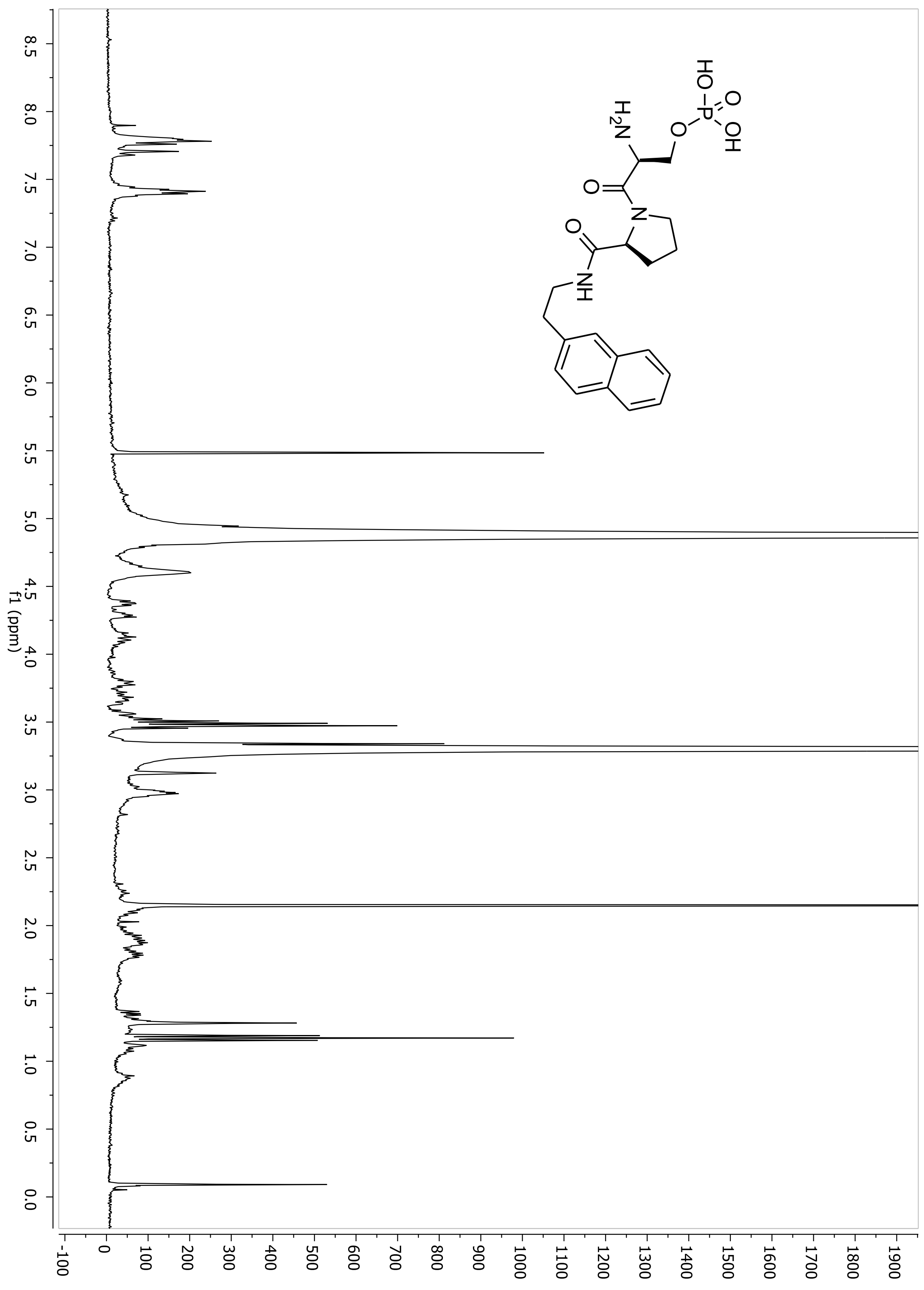

^1H NMR of pSer–Pro–(2-(2-naphthyl)ethyl)-amide in CD_3OD (500 MHz)

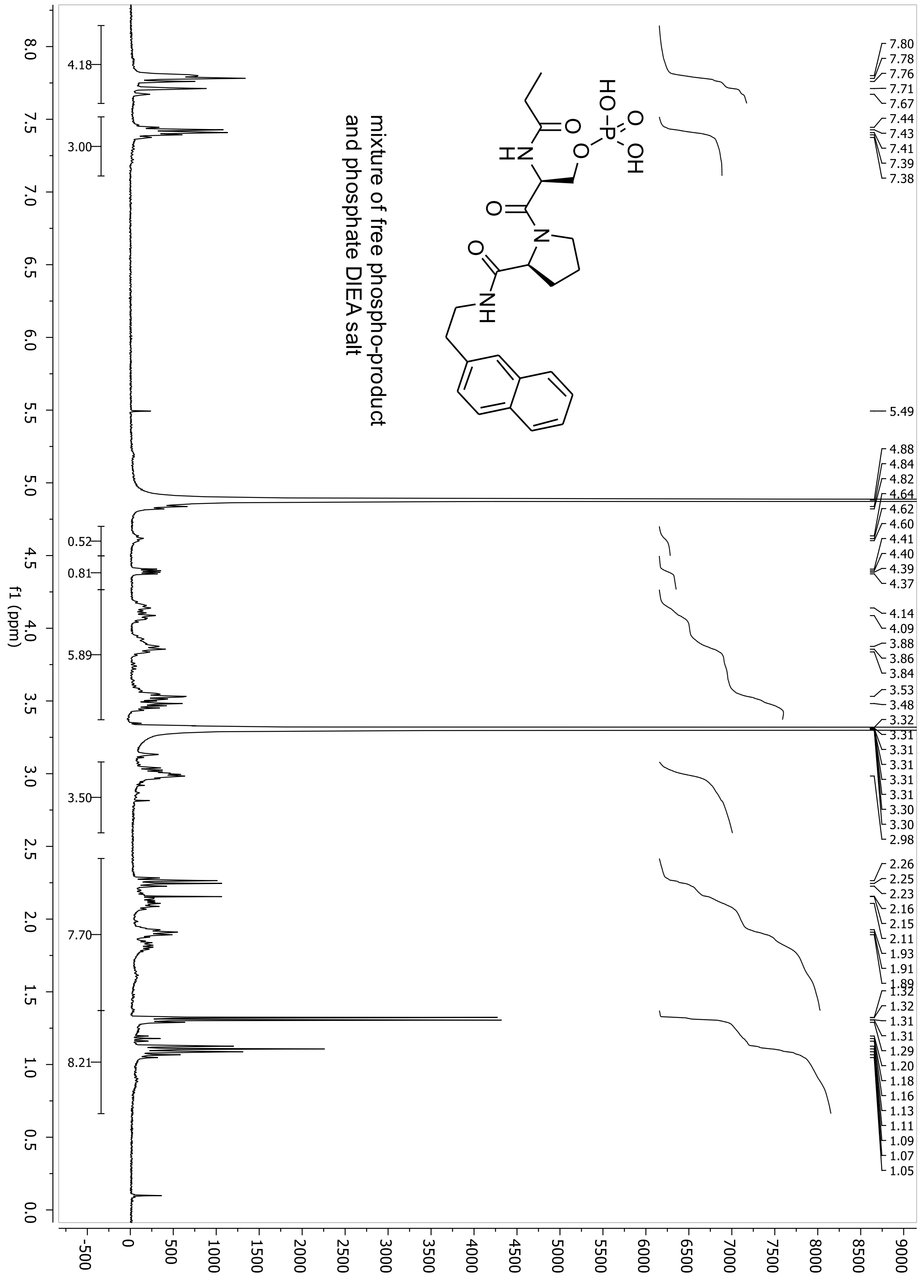

^{1}H NMR of propanoyl-pSer–Pro–(2-(2-naphthyl)ethyl)-amide in CD_3OD (500 MHz)

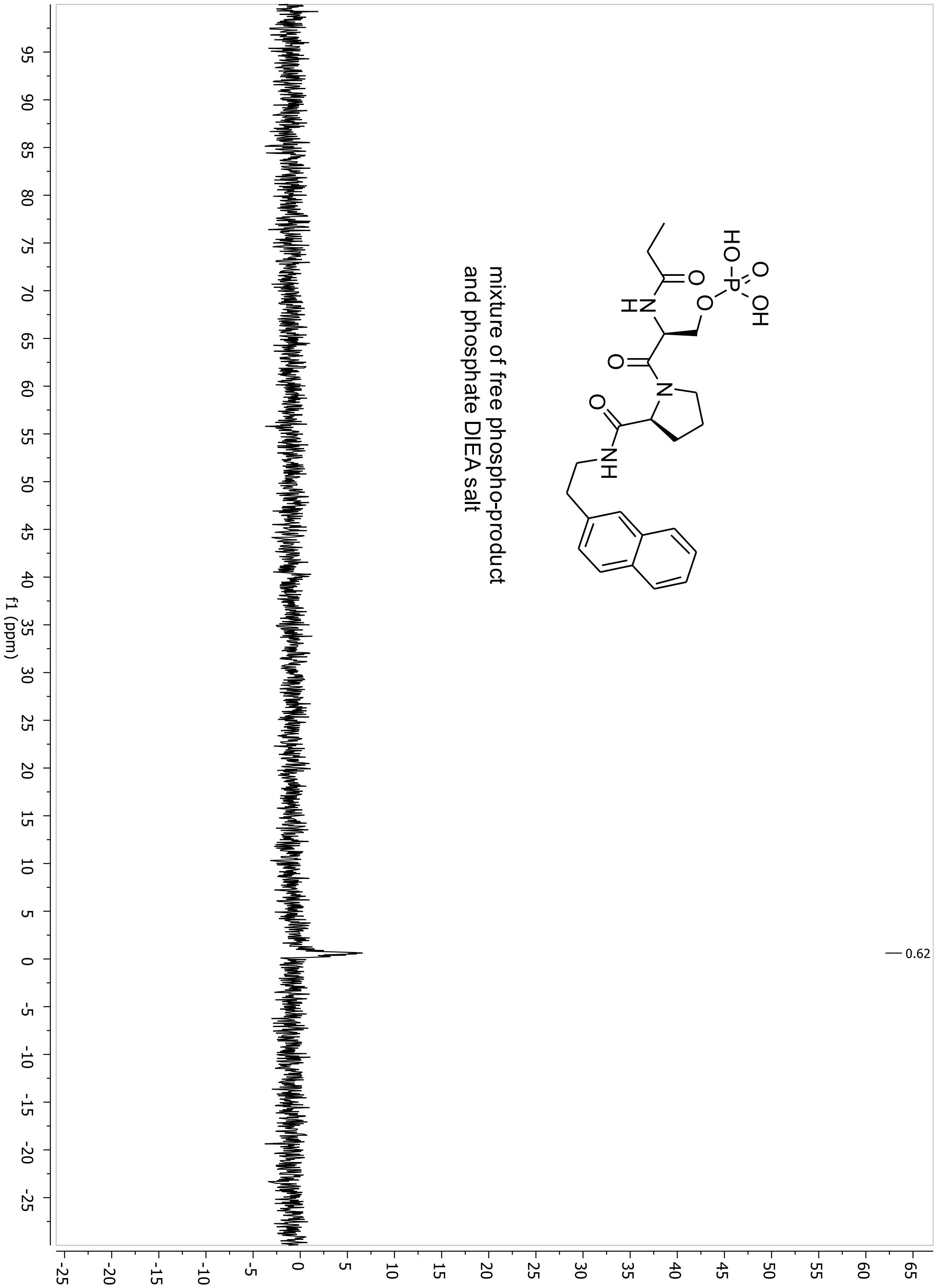

^{31}P NMR of propanoyl-pSer–Pro–(2-(2-naphthyl)ethyl)-amide in CD_3OD (202 MHz)

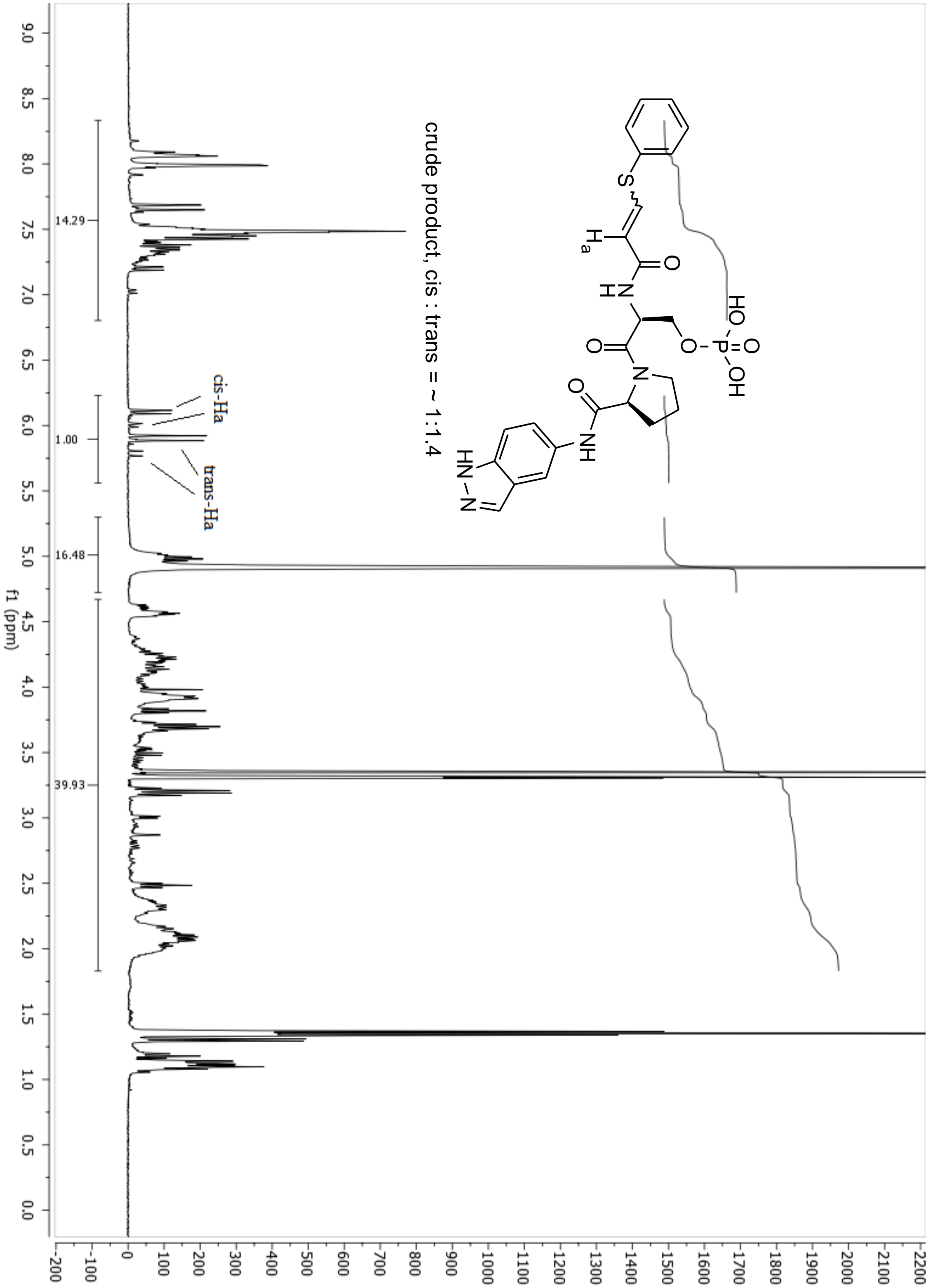

^{1}H NMR of crude product of **1**{*2*, *d*} in CD_3OD (500 MHz)

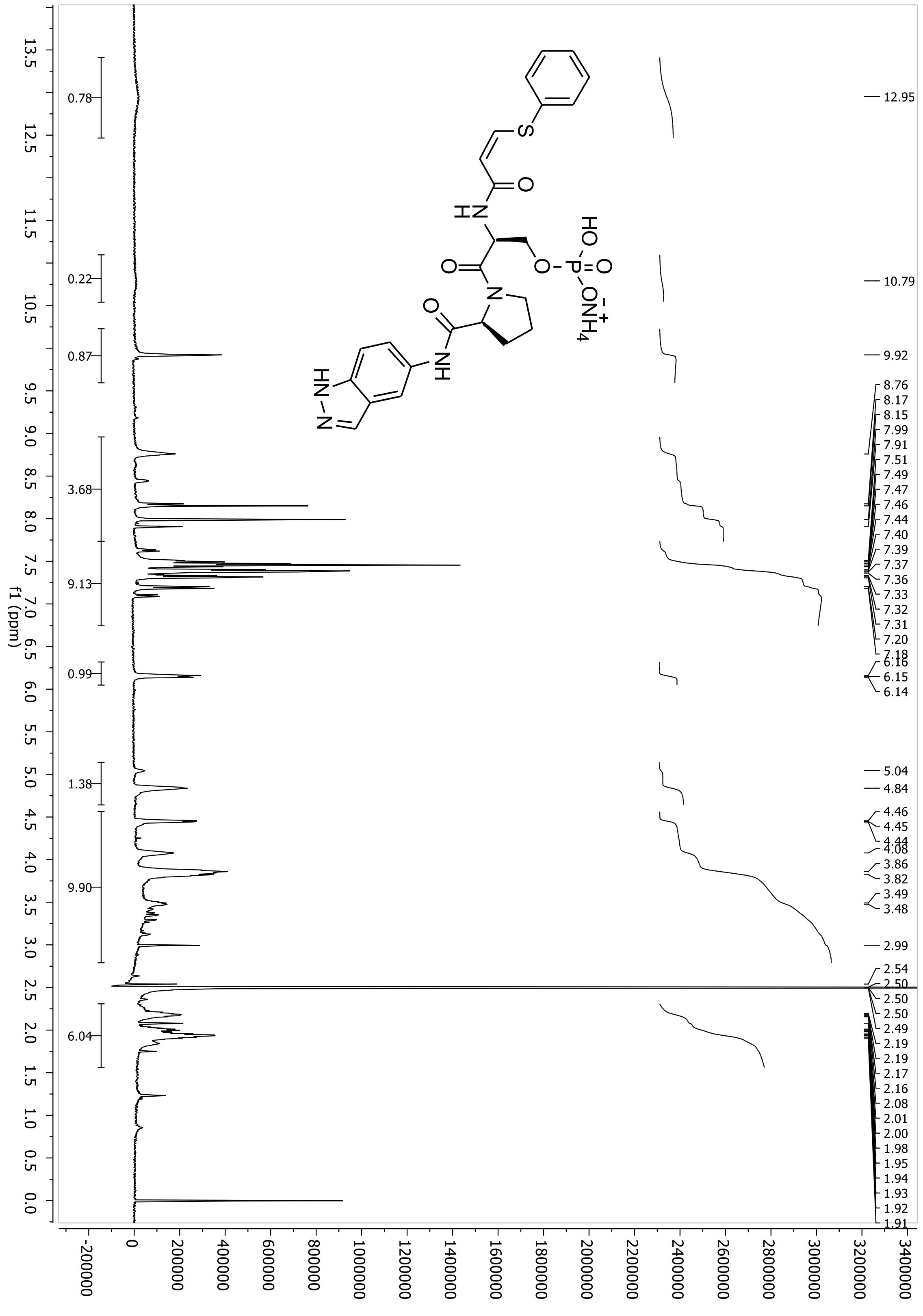

^{1}H NMR of *cis*-**1**{*2*,*d*} in DMSO-d6 (500 MHz)

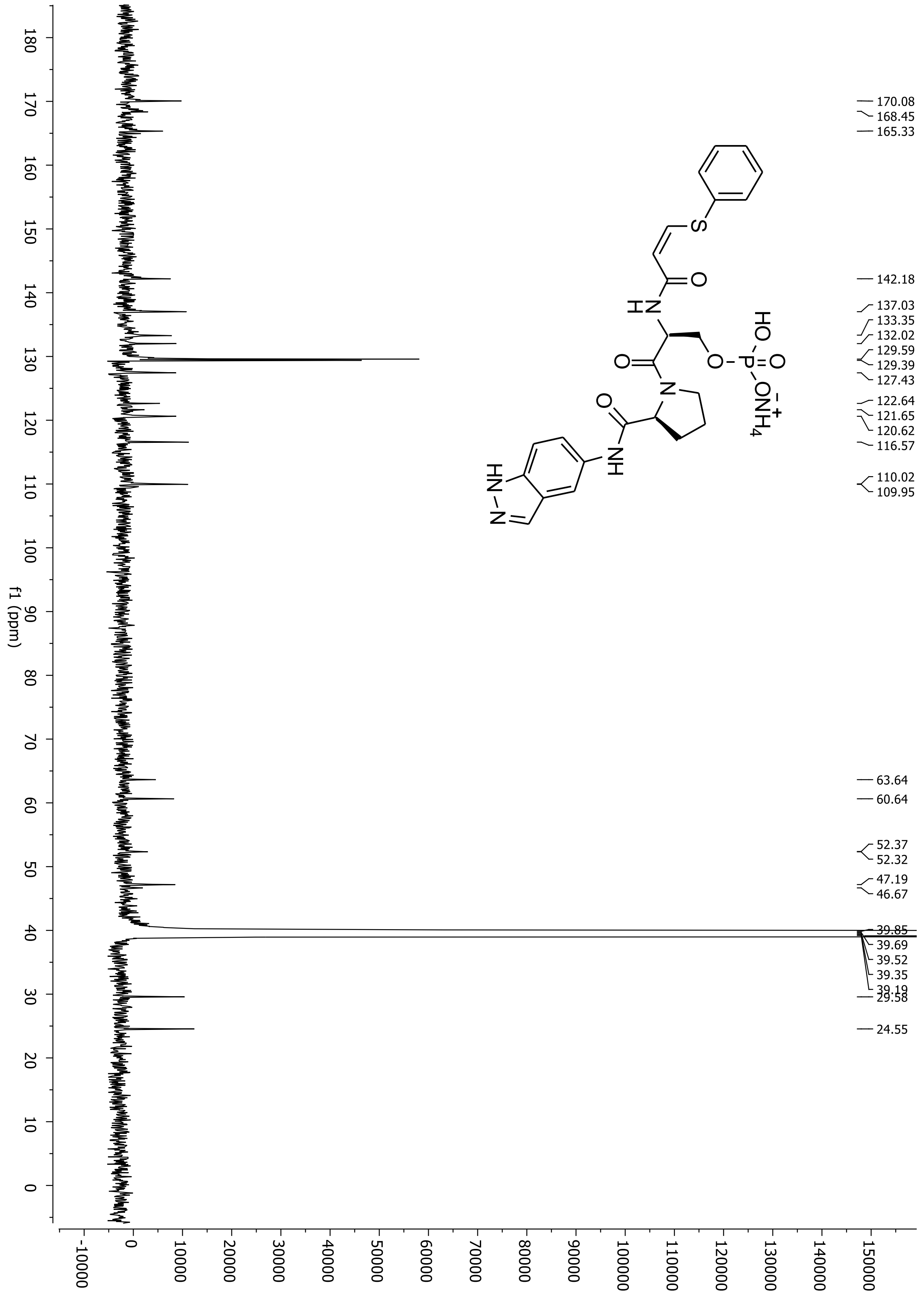

^{13}C NMR of *cis*-**1**{*2*,*d*} in DMSO-d6 (125 MHz)

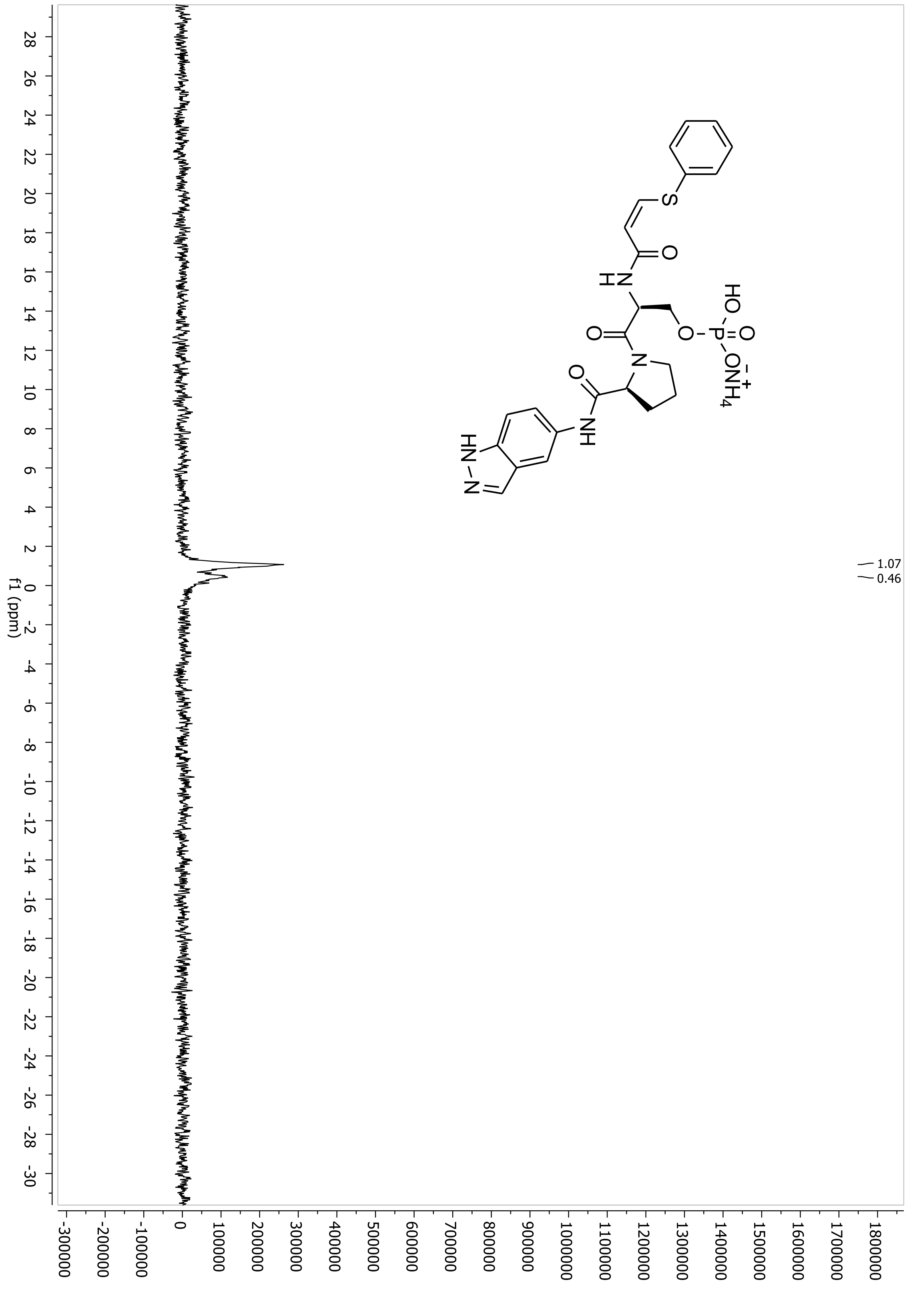

^{31}P NMR of *cis*-**1**{*2*,*d*} in DMSO-d6 (202 MHz)

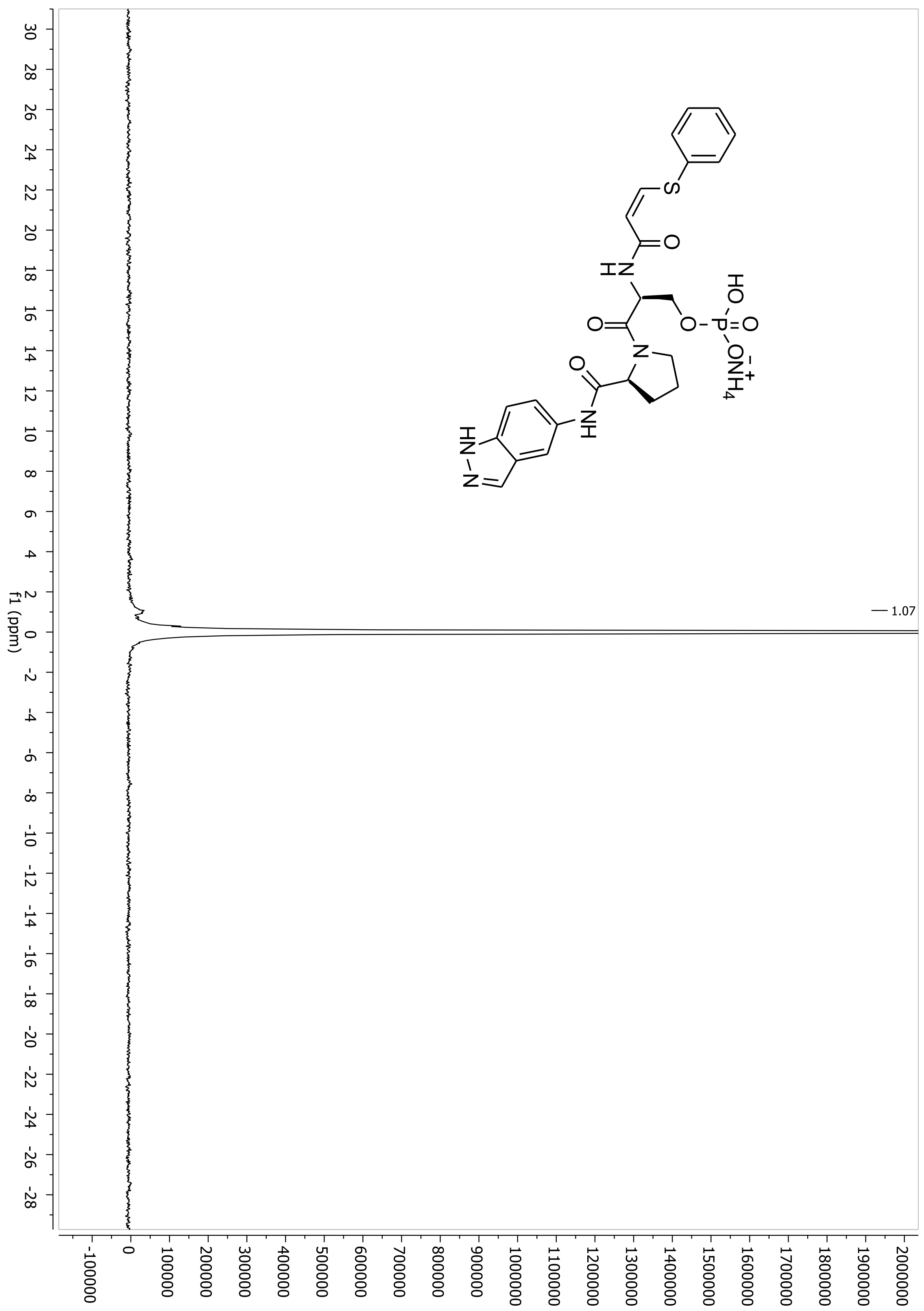

^{31}P NMR of *cis*-**1**{*2*,*d*} in DMSO-d6 (202 MHz)

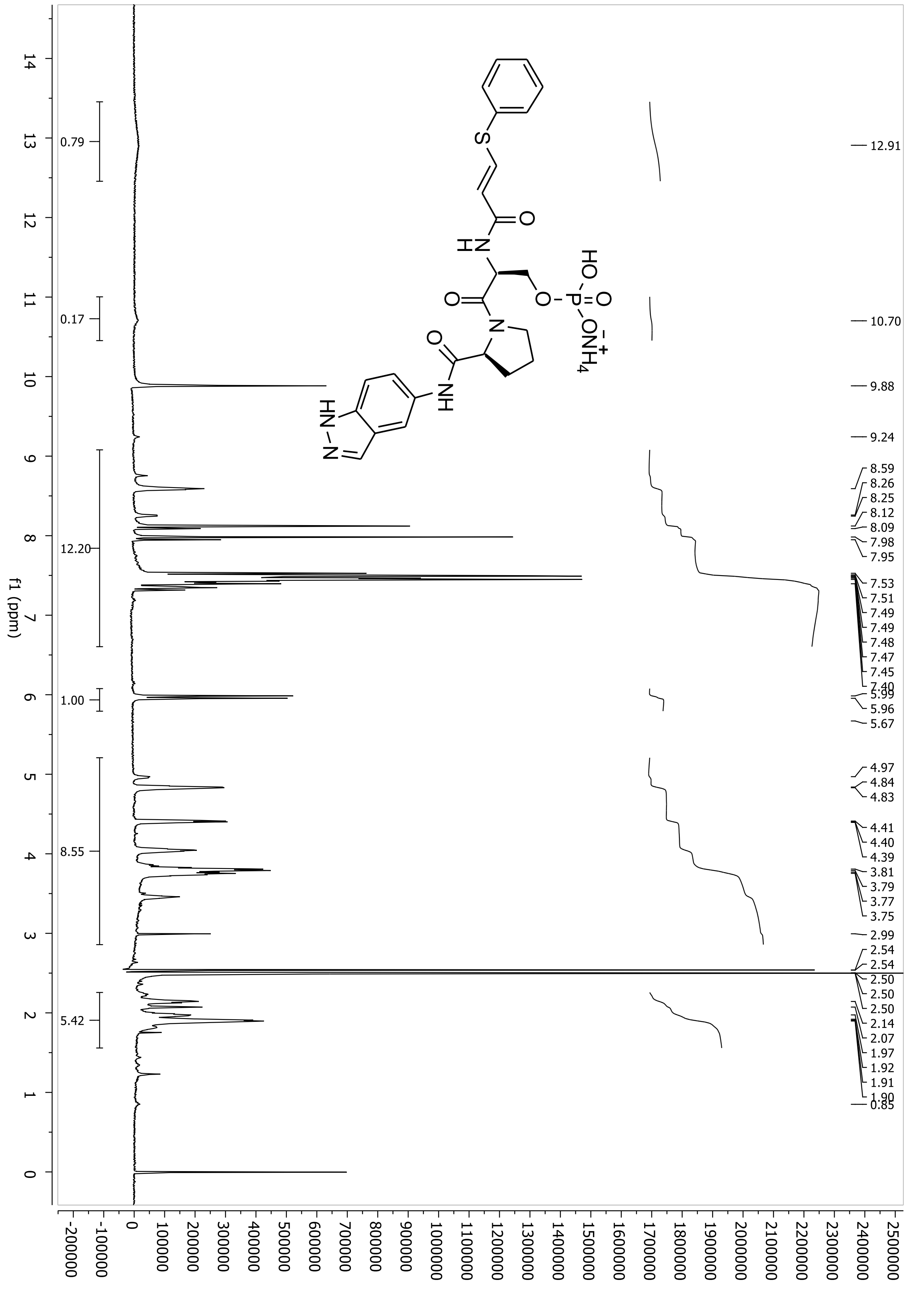

^{1}H NMR of *trans*-**1**{*2,d*} in DMSO-d6 (500 MHz)

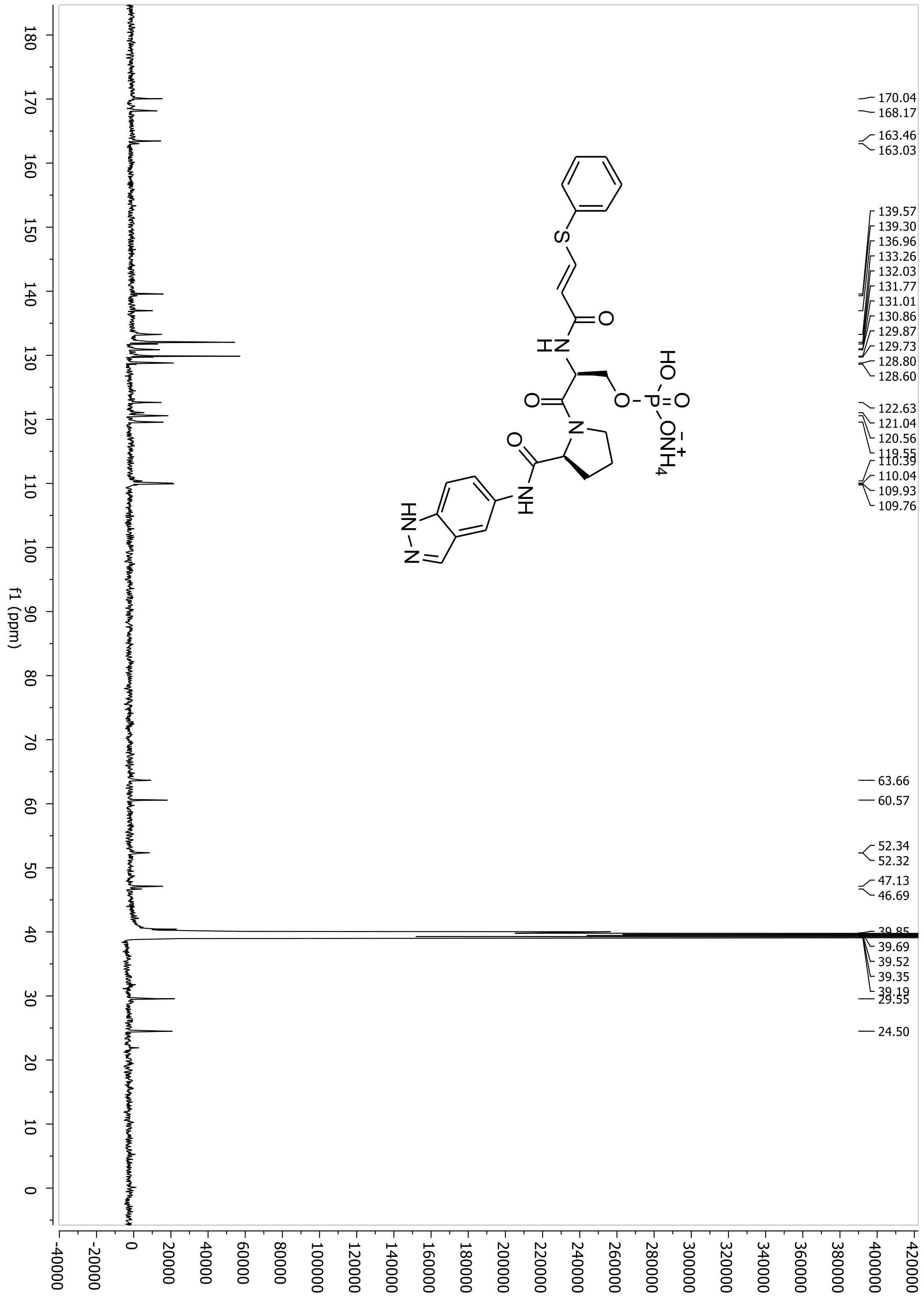

^{13}C NMR of *trans*-**1**{*2*,*d*} in DMSO-d6 (125 MHz)

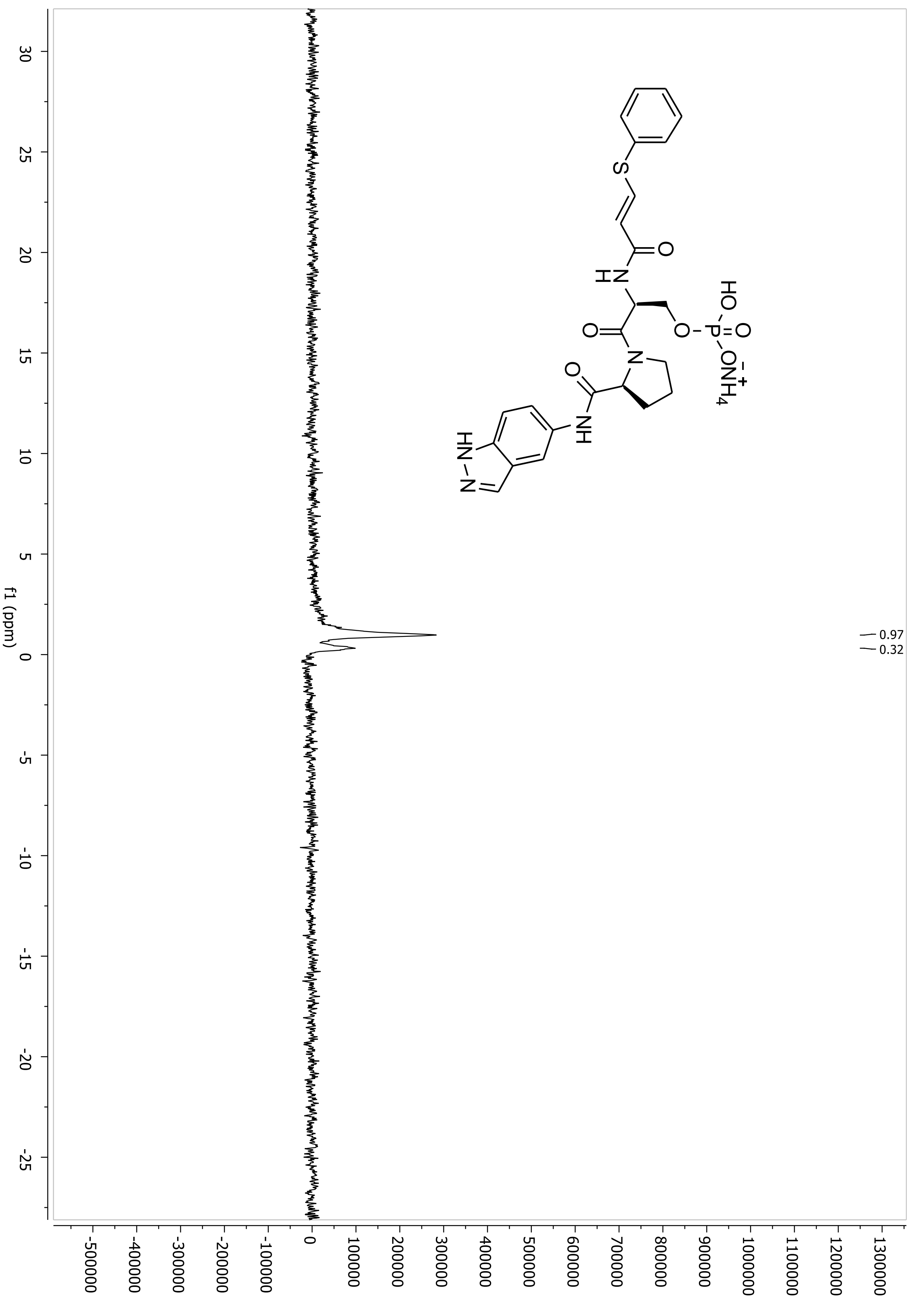

^{31}P NMR of *trans*-**1**{*2*,*d*} in DMSO-d6 (202 MHz)

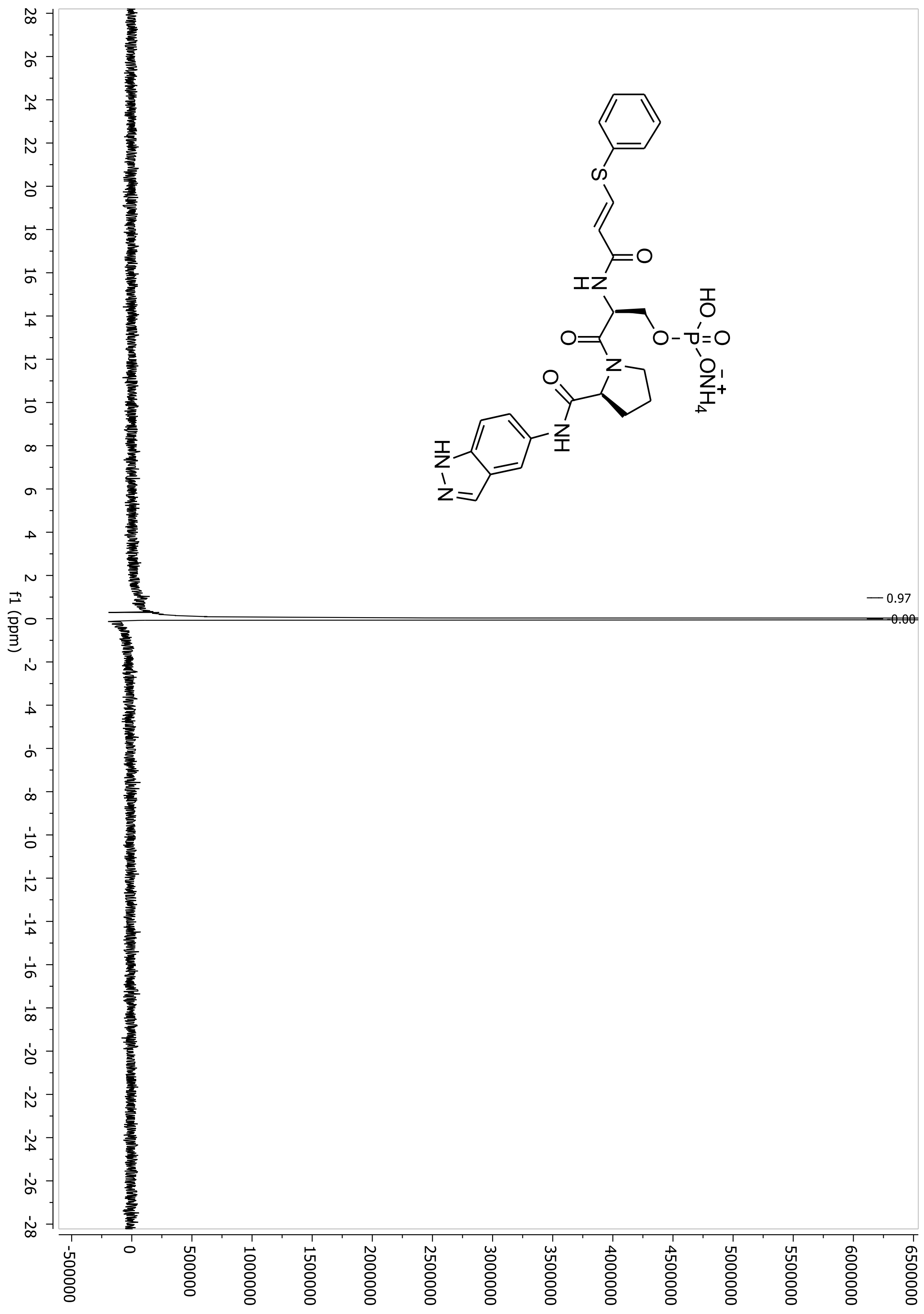

^{31}P NMR of *trans*-**1**{*2*,*d*} in DMSO-d6 (202 MHz)

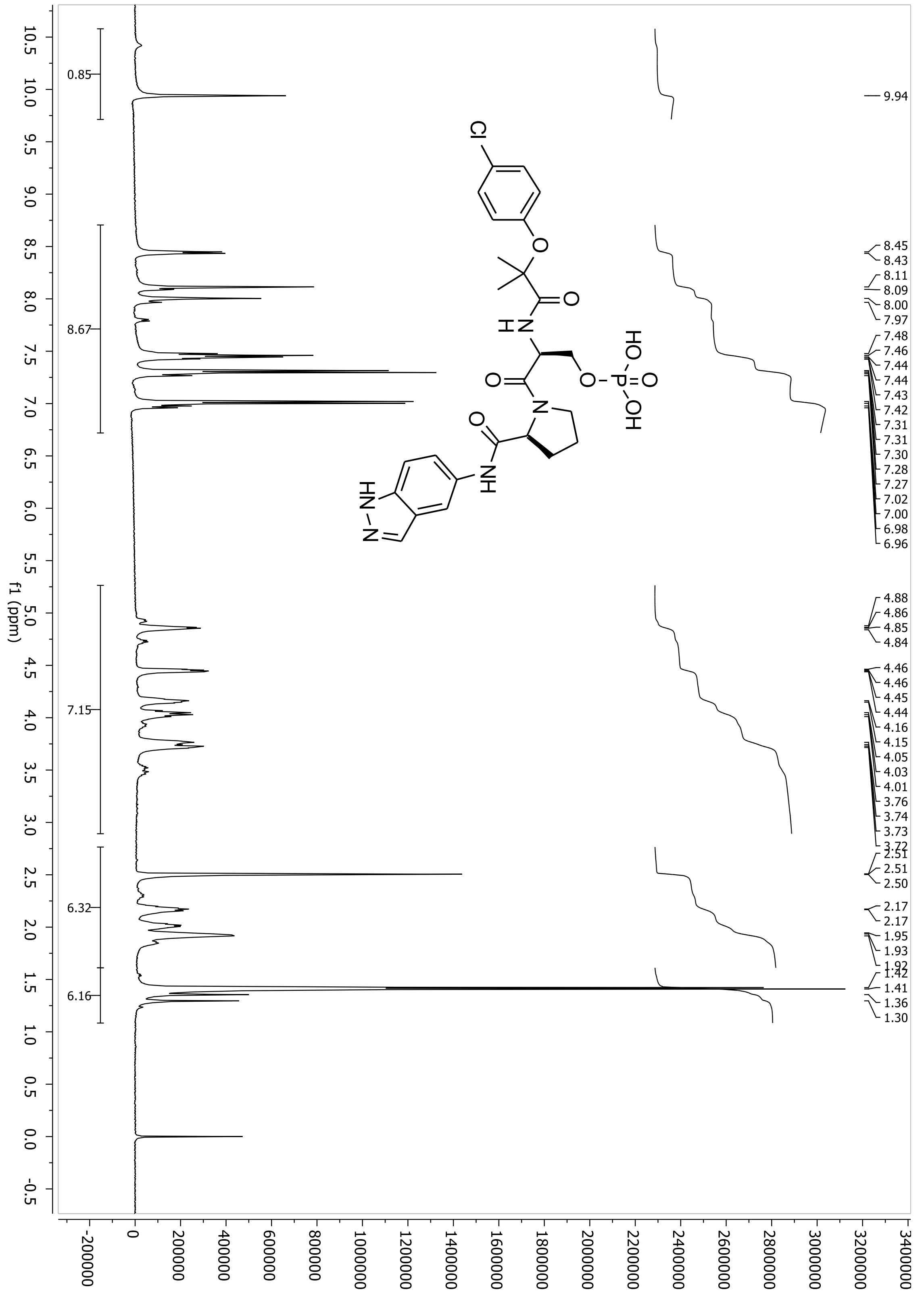

^{1}H NMR of **1**{*2*,*n*} in DMSO-d6 (500 MHz)

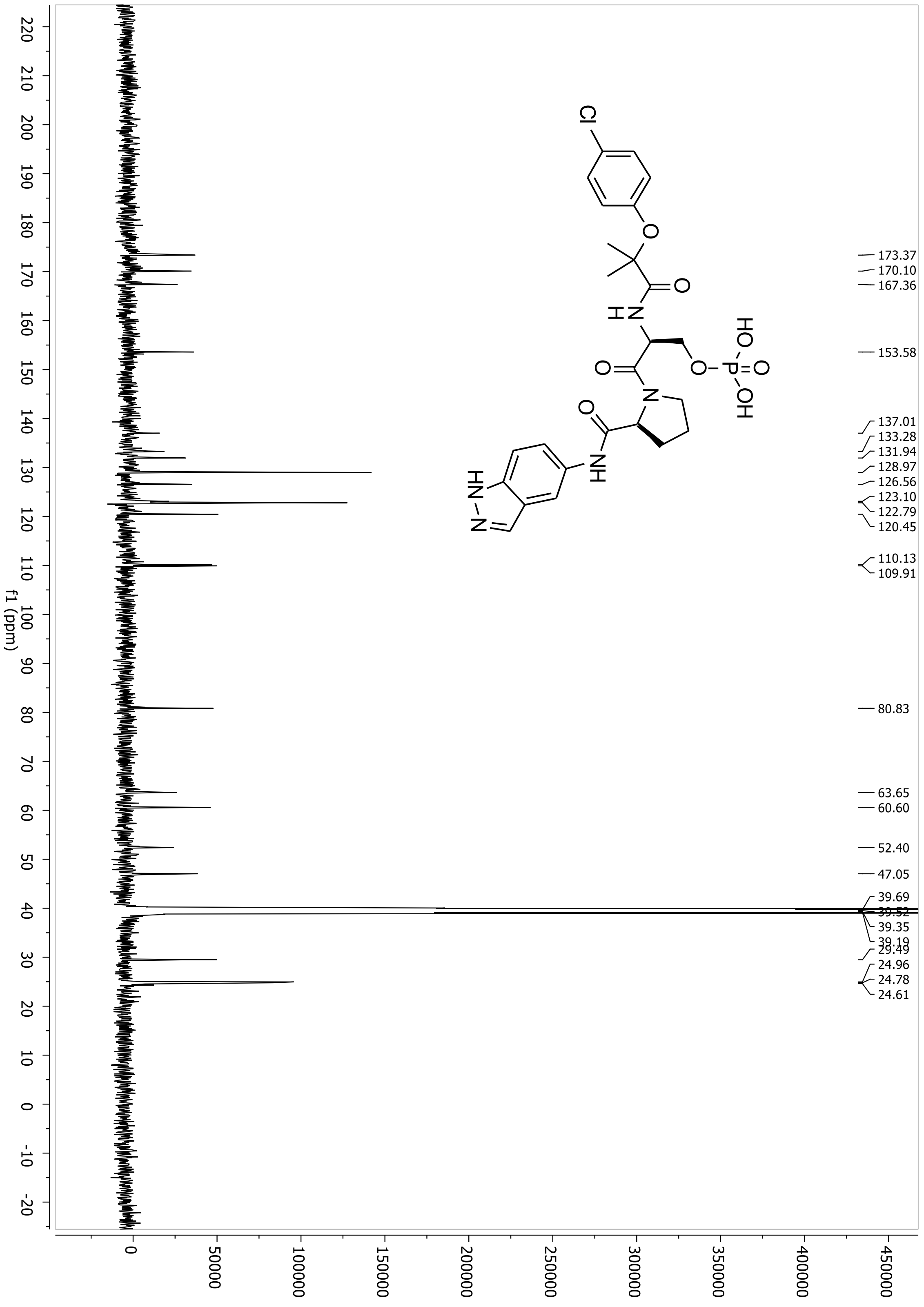

^{13}C NMR of **1**{*2*,*n*} in DMSO-d6 (125 MHz)

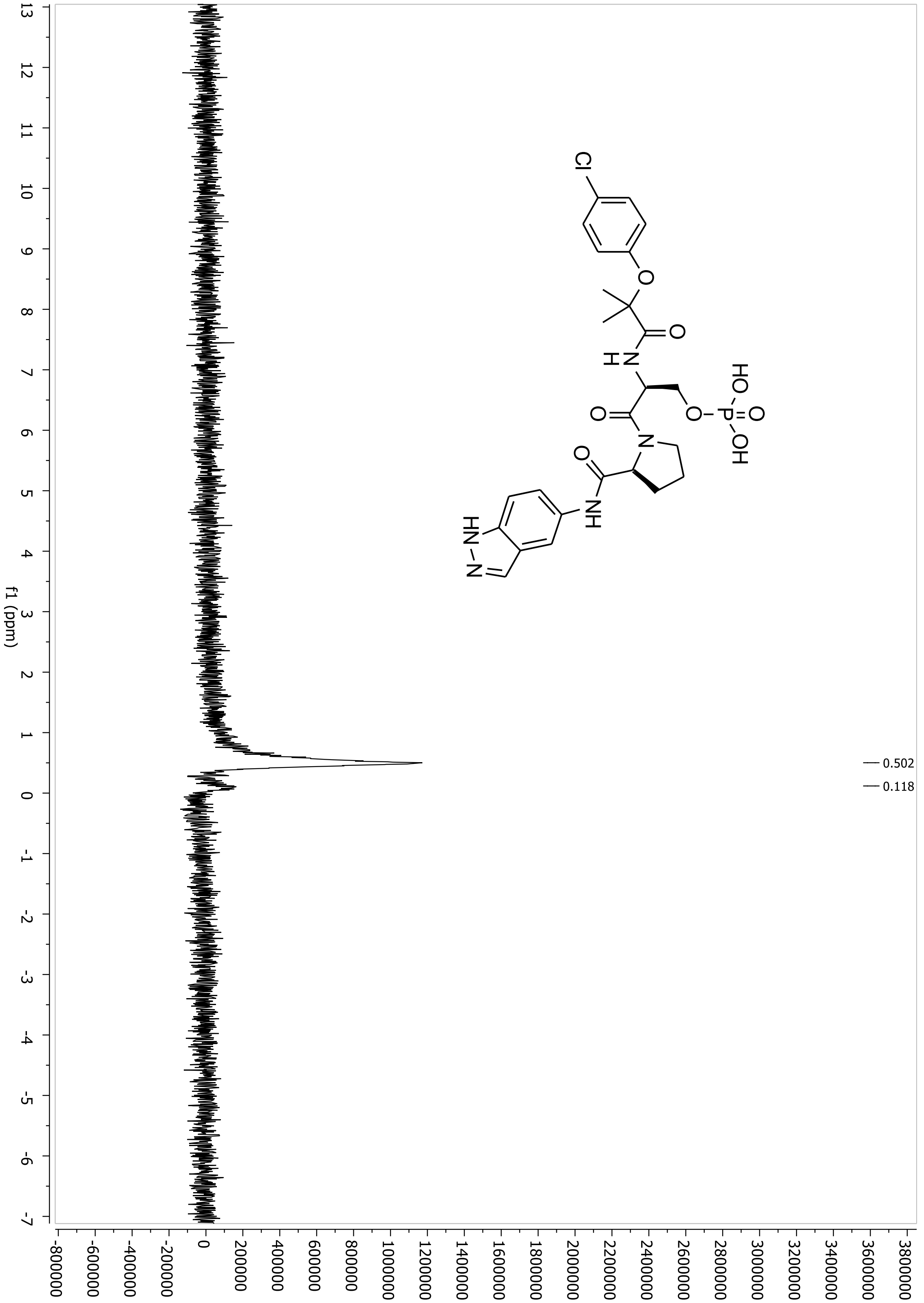

^{31}P NMR of **1**{*2*,*n*} in DMSO-d6 (202 MHz)

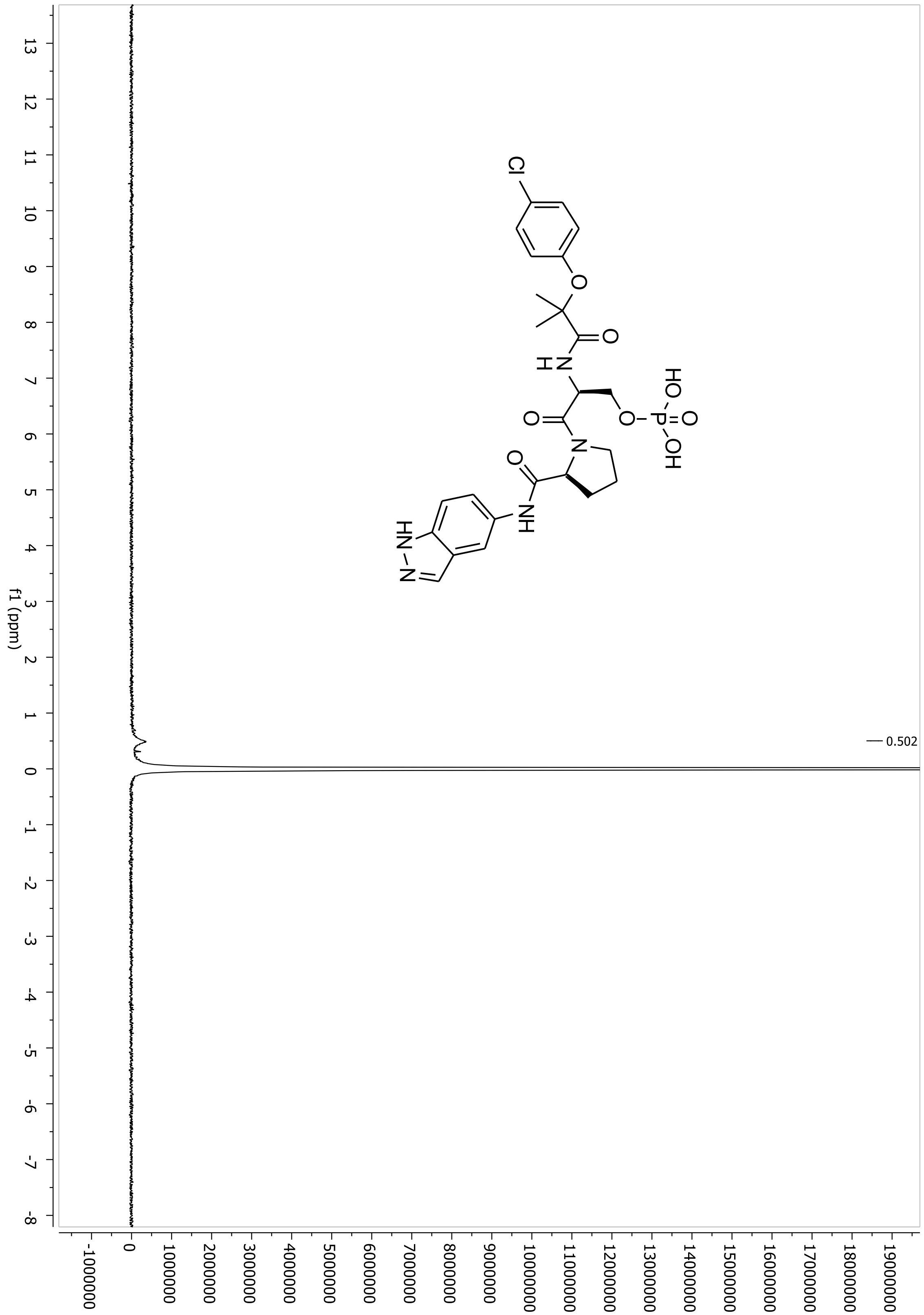

^{31}P NMR of **1**{*2*, *n*} in DMSO-d6 (202 MHz)

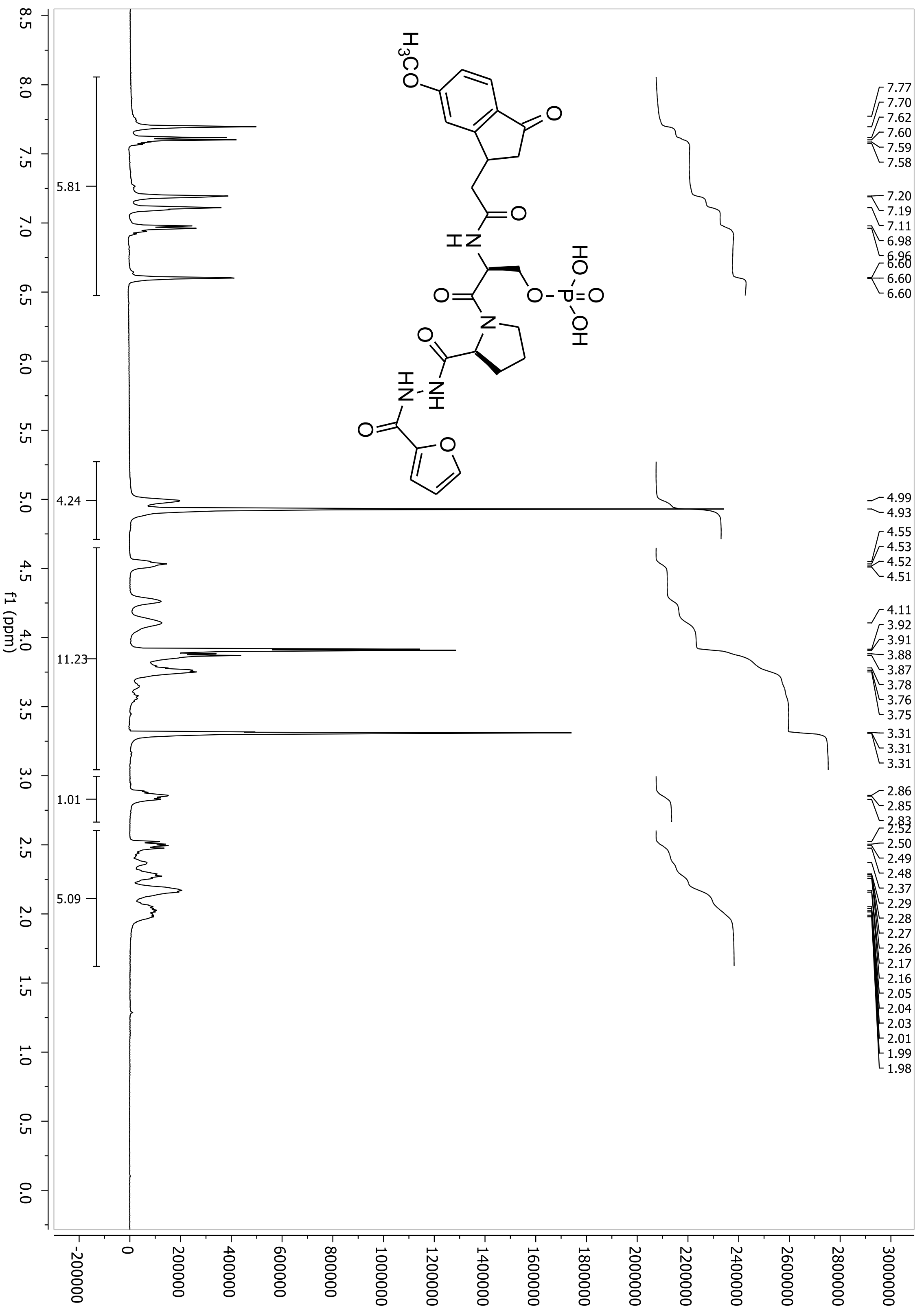

^{1}H NMR of **1**{*18,l*} in CD_3OD (500 MHz)

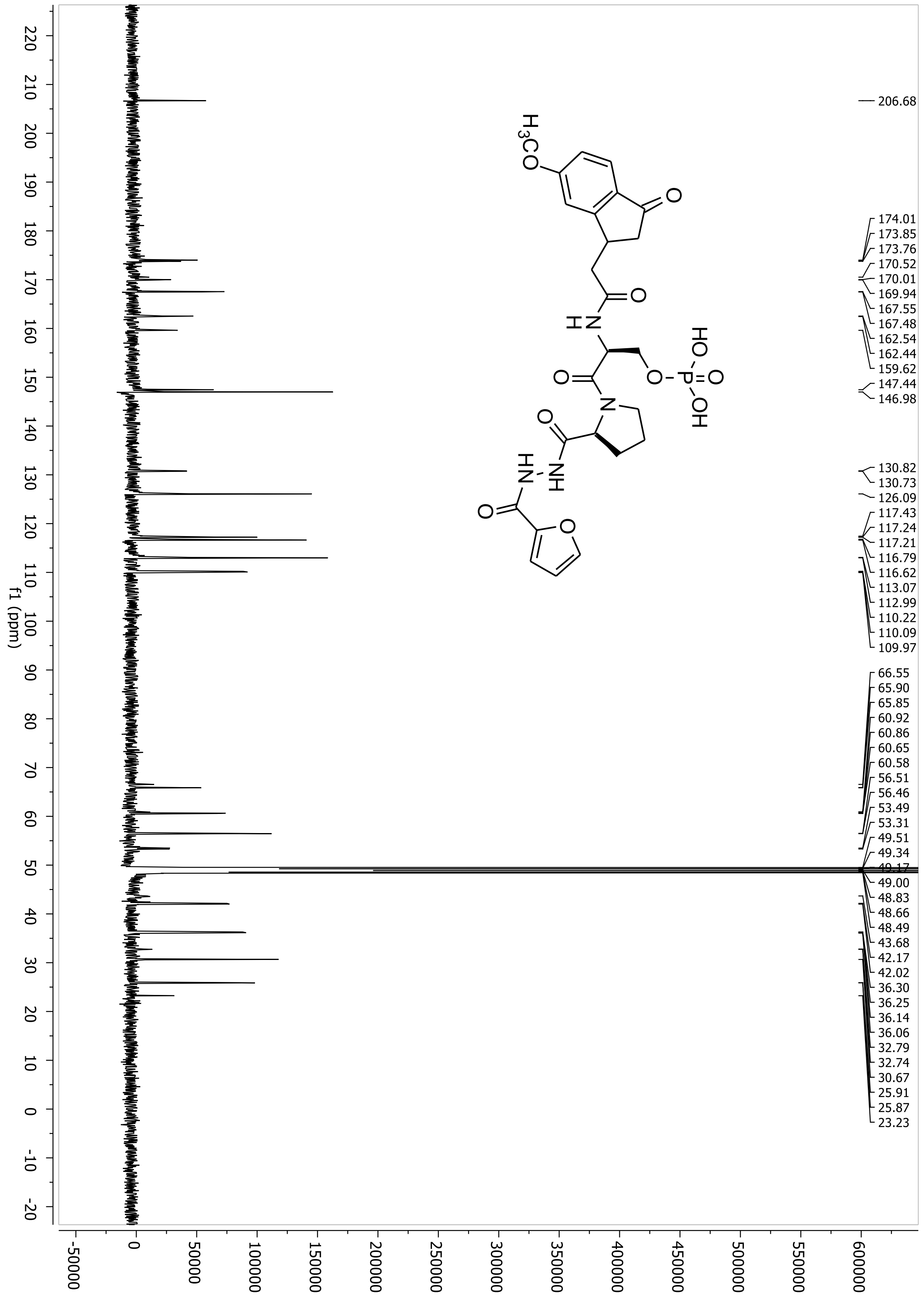

^{13}C NMR of **1**{*18,l*} in CD_3OD (125 MHz)

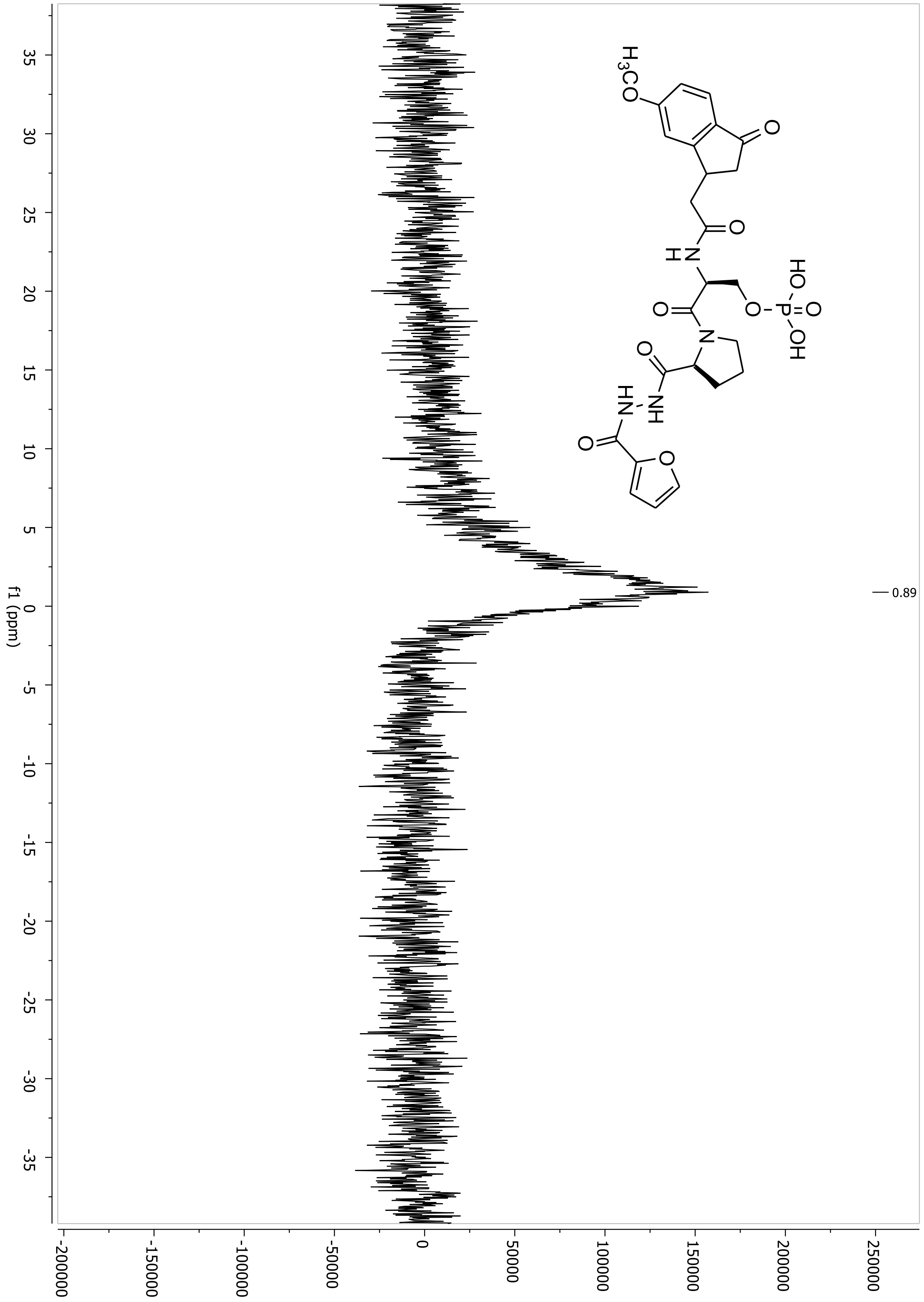

^{31}P NMR of **1**{*18*,*l*} in CD_3OD (202 MHz)

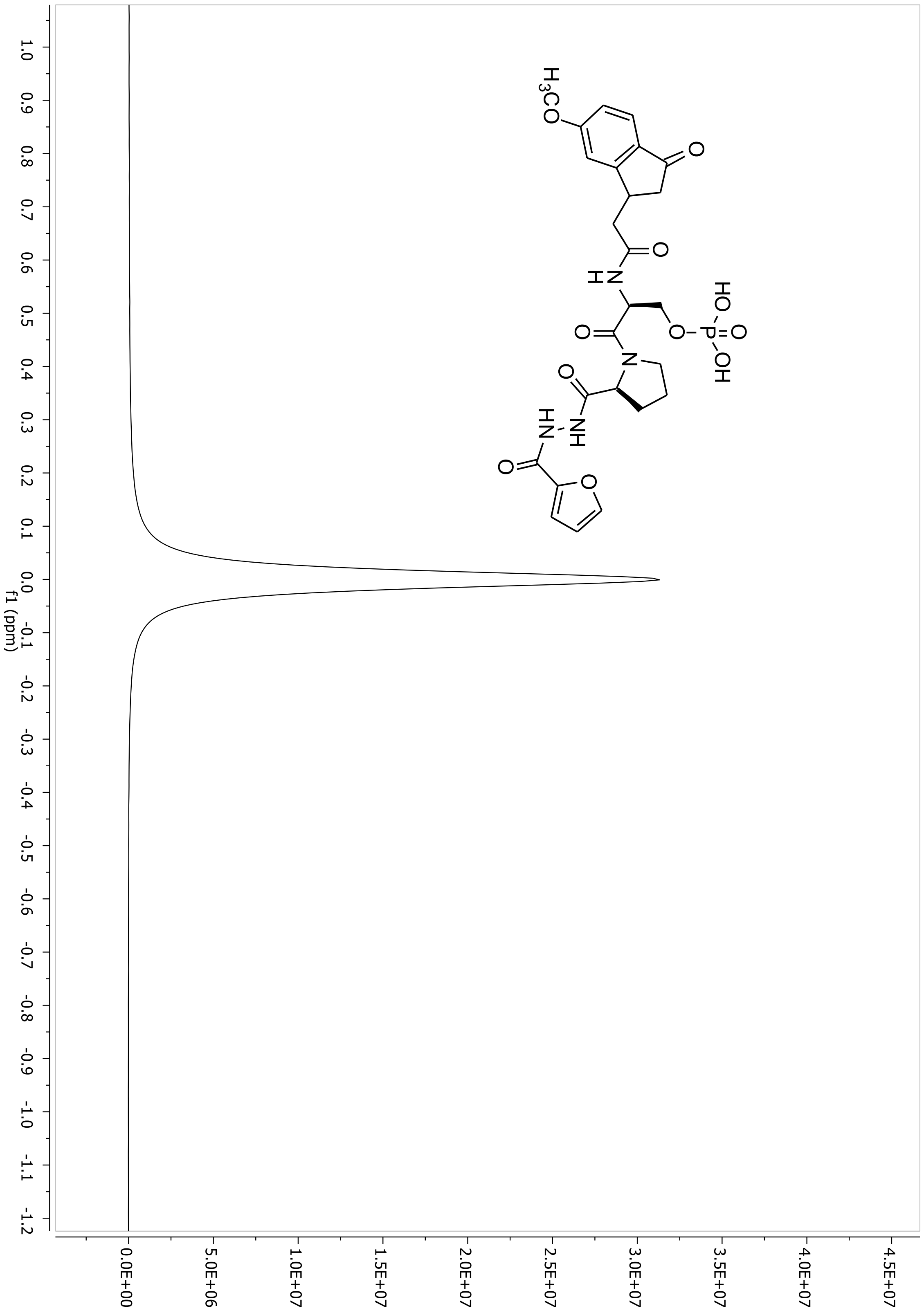

^{31}P NMR of **1**{*18,l*} in CD_3OD (202 MHz)
